# Prognostic and Clinicopathological Significance of FADD Upregulation in Head and Neck Squamous Cell Carcinoma: A Systematic Review and Meta-Analysis

**DOI:** 10.3390/cancers12092393

**Published:** 2020-08-24

**Authors:** Miguel Ángel González-Moles, Ángela Ayén, Isábel González-Ruiz, Teresa de Porras-Carrique, Lucía González-Ruiz, Isábel Ruiz-Ávila, Pablo Ramos-García

**Affiliations:** 1 School of Dentistry, University of Granada, 18071 Granada, Spain; isagonzru@gmail.com (I.G.-R.); teresadeporras@hotmail.es (T.d.P.-C.); 2 Instituto de Investigación Biosanitaria ibs.Granada, 18012 Granada, Spain; 3 Dermatology Service, San Cecilio Hospital Complex, 18016 Granada, Spain; angelaayenr@gmail.com; 4 Dermatology Service, Ciudad Real General University Hospital, 13005 Ciudad Real, Spain; gruizlucia@gmail.com; 5 Pathology Service, San Cecilio Hospital Complex, 18016 Granada, Spain; iruizavila@gmail.com

**Keywords:** FADD, Fas-associated death domain, head and neck squamous cell carcinoma, oral squamous cell carcinoma, laryngeal squamous cell carcinoma, prognosis, biomarker, systematic review, meta-analysis

## Abstract

Fas-associated death domain (FADD) upregulation, i.e., gene amplification, protein phosphorylation and/or overexpression, has shown promising prognostic implications in head and neck squamous cell carcinoma (HNSCC). This systematic review and meta-analysis aims to evaluate the clinicopathological and prognostic significance of FADD upregulation in HNSCC. We searched studies published before February 2020 through PubMed, Embase, Web of Science, Scopus and Google Scholar. We evaluated the quality of the studies included using the QUIPS tool. The impact of FADD upregulation on survival and clinicopathological variables was meta-analysed. We explored heterogeneity and their sources, conducted sensitivity analyses and investigated small-study effects. Thirteen studies (1,923 patients) met inclusion criteria. FADD immunohistochemical overexpression was statistically associated with worse overall survival (hazard ratio [HR] = 1.52, 95% confidence intervals [CI] = 1.28–1.81, *p* < 0.001), disease-specific survival (HR = 2.52, 95% CI = 1.61–3.96, *p* < 0.001), disease-free survival (HR = 1.67, 95% CI=1.29–2.15, *p* < 0.001), higher clinical stage (odds ratio [OR] = 1.72, 95% CI = 1.17–2.51, *p* = 0.005) and a large magnitude of effect with N+ status (OR = 2.36, 95% CI = 1.85–3.00, *p* < 0.001). FADD phosphorylation in ser-194 demonstrated no prognostic value, while no conclusive results can be drawn for FADD gene amplification. In conclusion, our findings indicate that immunohistochemical assessment of FADD overexpression could be incorporated into the prognostic evaluation of HNSCC.

## 1. Introduction

Head and neck cancer, the seventh most common cancer worldwide, is responsible for 890,000 new cases and 450,000 deaths per year (GLOBOCAN, IARC, WHO) [1]. Head and neck squamous cell carcinoma (HNSCC) represents >90% of these malignant neoplasms [2] and constitutes a heterogeneous group of tumours with anatomical, clinical, histopathological and molecular differences [3]. Prediction of the prognosis for individual patients is highly important in HNSCC, given its poor 5-year survival rate (around 50%) [4]. HNSCC prognostic and decision making is currently based on the tumour node metastasis staging system, and emerging biomarkers harbouring prognostic significance are attracting considerable research interest [5,6].

Fas-associated death domain (FADD) protein is encoded by FADD gene in chromosomal band 11q13 [7]. The amplification of the locus 11q13 is a relevant oncogenic mechanism in head and neck carcinogenesis due to its frequent upregulation (which is more than double the frequency found in other human cancers) and its prognostic implications (e.g., positive-N status or acquired resistance to chemotherapy and radiotherapy) [7]. FADD overexpression was initially described as an adaptor molecule for death receptor-mediated apoptosis. FADD regulates the activation of so-called active death receptors (e.g., Fas or DR4/5), which bind with procaspases, promoting the formation of death inducing signalling complexes [8]. More recently, FADD has been shown to play a key emergent role in the regulation of cell proliferation—via cell cycle control—and signalosome complexes, such as the necroptosome and the inflammasome, which could better explain its implication in cancer poor prognosis [9]. Different posttranslational modifications of FADD protein have also been reported, but the most relevant and well-studied is the phosphorylation of serine 194 residue (pFADD) [10]. The phosphorylation status of FADD does not seem to affect apoptosis and has also been related with poor prognosis in lymphomas and lung cancer via NF-kB-cyclin D1 activation [11,12]; nevertheless, its impact on HNSCC is controversial [13].

The prognostic relevant implications of FADD upregulation (gene amplification, protein phosphorylation and/or overexpression) were early reported in cancer [9]. In addition to *CCND1*/cyclin D1 and *CTTN*/cortactín [14,15], FADD has been proposed as a potential 11q13 amplicon driver (i.e., an amplified gene invariably found to be amplified and overexpressed, for which overexpression confers advantages to the host cell and contributes to the maintenance of the malignant phenotype) in human [16], larynx [17] and oral cancer [7]. Strikingly, in the last 5 years, FADD is booming, and this is due to the dataset reported by The Cancer Genome Atlas project (TCGA) [18] and subsequent massive bioinformatics analyses [19,20,21]. A comprehensive microarray data integration-based bioinformatics analysis using in silico tools (via Gene Expression Omnibus (GEO) and Array Express (EBI) public registers) identified FADD as one of the most promising biomarkers in HNSCC predicting poor prognosis [21]. The prognostic significance of FADD has also been validated in a recently published article reviewing the TCGA subsets for HNSCC, confirming once again its utility as a prognostic tool and its frequent upregulation (being considered by their authors as one of the “top five” altered genes in HNSCC [19]). Consequently, its therapeutic value in HNSCC has also started to be investigated, both as a single target [20,22] and in combination with anti-PD-L1 drugs [23,24].

Although their neighbouring codriver genes in the 11q13 chromosomal band—CCND1 and CTTN—have been recently meta-analysed by our group [25,26], showing their promising utility as prognostic tools in HNSCC, there is not enough evidence on the prognostic significance of FADD upregulation in HNSCC. With this background and mainly motivated by the dataset reported by TCGA project, we decided for first time to conduct a systematic review and meta-analysis for the qualitative and quantitative analysis of current scientific evidence on the potential translational opportunities of FADD upregulation in HNSCC. Therefore, following the PECOTS framework (population (P), exposure (E), comparator (C), outcome (O), timing (T) and setting (S)), our objective was to investigate if patients with HNSCC (P) harbouring FADD upregulation (FADD/pFADD overexpression or gene amplification) (E) compared to those patients without these alterations (C) have a worse prognosis (overall survival, disease-specific survival, disease-free survival and/or local recurrence) and/or clinicopathological characteristics (tumour size, N status, clinical stage and/or histological grade) (O); no restrictions by follow-up period or publication date (T), language or study design (S) were used.

## 2. Results

### 2.1. Results of the Literature Search

**Figure 1:**
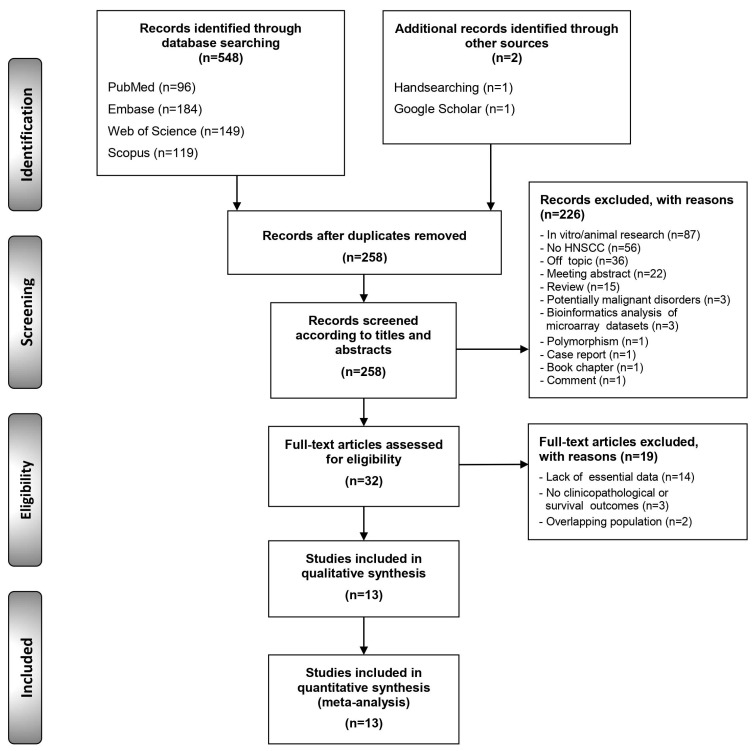
Flow diagram showing the identification and selection process of relevant studies, analysing the prognostic and clinicopathological significance of Fas-associated death domain (FADD) alterations in head and neck squamous cell carcinoma (HNSCC).

The flow diagram in Figure 1 depicts the study selection process and the results obtained. A total of 550 publications were retrieved: 96 from PubMed, 184 from Embase, 149 from the Web of Science, 119 from Scopus, 1 from the reference list screening [27] and 1 from Google Scholar [28]. After duplicate removal, 259 records were considered potentially eligible and their titles and abstracts were screened, leaving a sample of 32 studies for full-text evaluation (all the studies excluded—and their exclusion criteria—are listed in the Supplementary, Lists S3 and S4). Finally, 13 studies meeting all eligibility criteria were included for qualitative evaluation and meta-analysis (the references of the studies included are also listed in the Supplementary, List S5).

### 2.2. Study Characteristics

Table 1 summarizes their main characteristics, and Table S2 (Supplementary) exhibits in more detail the variables gathered from each study. The 13 studies included a total of 1923 patients; FADD amplification was studied by 4 studies (563 patients), pFADD overexpression was studied by 3 studies (285 patients) and FADD overexpression was studied by 11 studies (1727 patients) (please note that more than one alteration was analysed per study; Table S2). Sample sizes ranged between 30 and 339 patients. The role of FADD upregulation was explored in oral SCC by five studies, in laryngeal SCC by four studies, in mixed HNSCCs by three studies (subgroup of studies on combinations of these SCCs) and in nasopharyngeal SCC by one study. The studies were conducted in Europe (*n* = 7), Asia (*n* = 4) and North America (*n* = 2). In relation to their design, all were observational retrospective studies (*n* = 13). In immunohistochemical studies, six evaluated FADD overexpression both in the nucleus and cytoplasm (mixed pattern), although five evaluated only its cytoplasmic immunostaining; the A66-2 antibody was used by six of them, H181 was used in three and clone 556402 was used in one, while two studies did not report it. On the other hand, two and one studies evaluated pFADD nuclear and mixed immunostaining, respectively, all using a specific clone FADD phosphorylated at the Ser194 residue. The cut-off point was heterogeneous among studies (Table S2).

Table S2 (in the Supplementary) exhibits the characteristics of each study.

### 2.3. Qualitative Evaluation

The qualitative analysis was conducted using the QUIPS tool (Figures S1 and S2, List S1, Supplementary) which evaluates potential sources of bias in six domains (the risk of bias across studies for each domain was explained in more detail in List S1). According to our judgments using this tool, the domain 5 (also known as study confounding) harboured the highest risk of potential bias (Figure S2), targeting the failure to consider or measure potentially confounding factors. The overall quality of studies was acceptable, and only 3 studies harboured a higher overall risk of bias [27,28,29] (Figures S2 and S3).

### 2.4. Quantitative Evaluation (Meta-Analysis)

All variables considered for meta-analysis were graphically represented using forest plots (Supplementary), and their results are listed in Table 2.

#### 2.4.1. Association between FADD Upregulation and Prognostic Variables

Overall survival (OS): Significant results were found for FADD upregulation with poor OS (hazard ratio [HR] = 1.45, 95% confidence intervals [CI] = 1.16–1.81, *p* < 0.001), although a considerable degree of heterogeneity was present (*p* < 0.001, I2 = 74.3%), indicating that FADD alterations do not all have the same prognostic value. After the stratified analysis by type of alteration, the groups were more homogeneous. Both FADD overexpression (HR = 1.52%, 95% CI = 1.28–1.81, *p* < 0.001) and gene amplification maintained their significance (HR = 1.53%, 95% CI = 1.10–2.12, *p* = 0.01). Protein overexpression showed the most consistent result, showing no detectable heterogeneity (*p* = 0.50, I2 = 0.0), having been analysed an acceptable number of studies (*n* = 7) with a high number of patients (*n* = 1196 patients) (Table 2, Figure S3, Supplementary).

Disease-specific survival (DSS): No heterogeneity between studies was detected among FADD upregulation (*p* = 0.56, I2 = 0.0), and a significant association was found with DSS (HR = 2.63, 95% CI = 1.76–3.92, *p* < 0.001). The FADD overexpression group obtained similar results (HR = 2.52, 95% CI = 1.61–3.96, *p* < 0.001), while pFADD was only assessed by one study for this parameter (Table 2, Figure S12, Supplementary).

Disease-free survival (DFS). No interstudy heterogeneity was found among FADD alterations (*p* = 0.53, I2 = 0.0), showing a significant relationship with DFS (HR = 1.57, 95% CI = 1.28–1.94, *p* < 0.001). Only the FADD overexpression group maintained significant results (HR = 1.67, 95% CI = 1.29–2.15, *p* < 0.001), while gene amplification—only one study—was underpowered (*p* = 0.08) (Table 2, Figure S13, Supplementary).

#### 2.4.2. Association between FADD Upregulation and Clinicopathological Variables

T status: No heterogeneity was observed (*p* = 0.57, I2 = 0.0), and strikingly, as for local recurrence, tumour size was not associated with FADD upregulation (*p* = 0.17). This points out that FADD alterations could be only associated with late-stage HNSCCs (Table 2, Figure S14, Supplementary).

**Figure 2:**
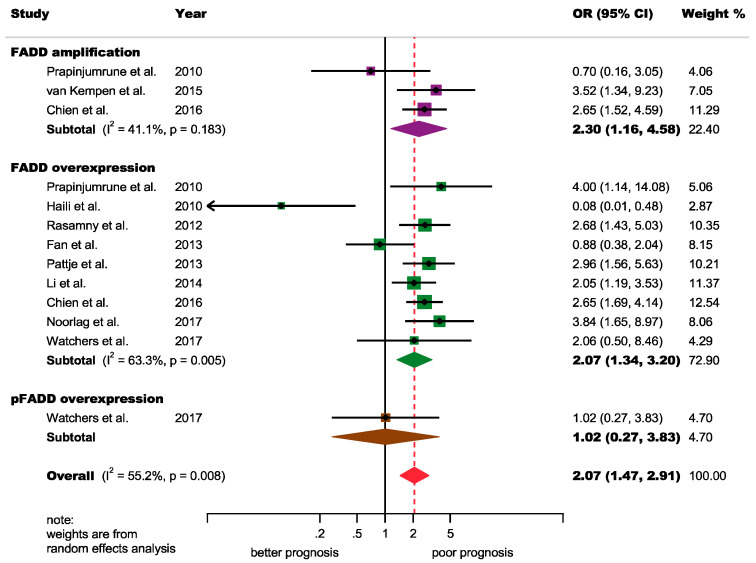
Forest plot of the association between FADD upregulation and N status in HNSCC (random-effects model and inverse-variance weighting based on the DerSimonian and Laird method): An OR > 1 suggests that FADD alterations are associated with positive-N status. Diamonds indicate overall ORs with associated 95% CIs. OR, odds ratio; CI, confidence intervals.

**Figure 3:**
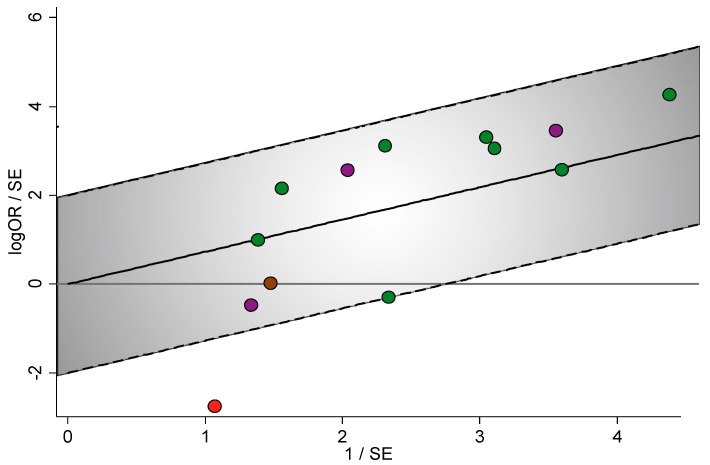
Galbraith plot of the association between FADD upregulation and N status in HNSCC, constructed to examine the contributions of individual studies to the heterogeneity metrics and identify outliers: The vertical axis represents the observed effect sizes standardized by their corresponding standard errors (y = logOR/SE[logOR]) against precision on the horizontal axis (x = 1/SE[logOR]). The regression diagonal line is projected from the origin (0,0), and the approximate 95% confidence intervals run between the two intermittent parallel lines at ±2 units above and below the regression line (grey region). The studies inside this 95% confidence region were represented as green (FADD overexpression), brown (pFADD overexpression) and purple (FADD amplification) circles. The study below the confidence limits (outside the grey region) was identified as a significant outlier (Haili et al., 2010, depicted as a red circle), contributing disproportionately to the observed heterogeneity. A copy of this plot showing additional information was included in the Supplementary (Figure S15), allowing an easier identification of studies. OR, odds ratio; SE, standard error.

N status: A significant association was found among FADD alterations and positive-lymph node metastasis (odds ratio [OR] = 2.07, 95% CI = 1.47–2.91, *p* < 0.001), although a certain degree of heterogeneity was found (*p* = 0.008, I2 = 55.2) (Figure 2). Fortunately, an outlier [28] was identified after performing visual inspection analysis of the forest plot and of the Galbraith plot (Figure 3). A subsequent sensitivity analysis (“leave-one-out” method) confirmed that, after the omission of this study, heterogeneity was markedly reduced (*p* = 0.27, I2 = 17.9 (−37.3%)) reaching nonsignificant levels (Figures S16 and S32, Table S8, Supplementary), so the main source of heterogeneity was detected. Furthermore, this outlier played great influence on the pooled estimate, since despite its low relative weight (2.9%), the meta-analytical results increased by 14% after its omission (OR = 2.36, 95% CI = 1.85–3.00, *p* < 0.001). The only salient characteristic of this study was reporting in a non-English language [28]. Furthermore, the significant association with N+ status was maintained for gene amplification (OR = 2.30, 95% CI = 1.16–4.58, *p* = 0.02) and FADD overexpression (OR = 2.42, 95% CI = 1.84–3.18, *p* < 0.001) groups (Figure S16).

Clinical stage: A significant association was for among FADD upregulation and advanced stage tumours (OR = 1.74, 95% CI = 1.26–2.41, *p* = 0.001) with no observable heterogeneity (*p* = 0.44, I2 = 0.0). Once again, only the protein expression group preserved this significant association (OR = 1.72, 95% CI = 1.17–2.51, *p* = 0.005) (Table 2, Figure S17, Supplementary).

### 2.5. Quantitative Evaluation (Variables Not Included in Meta-Analysis)

**Figure 4:**
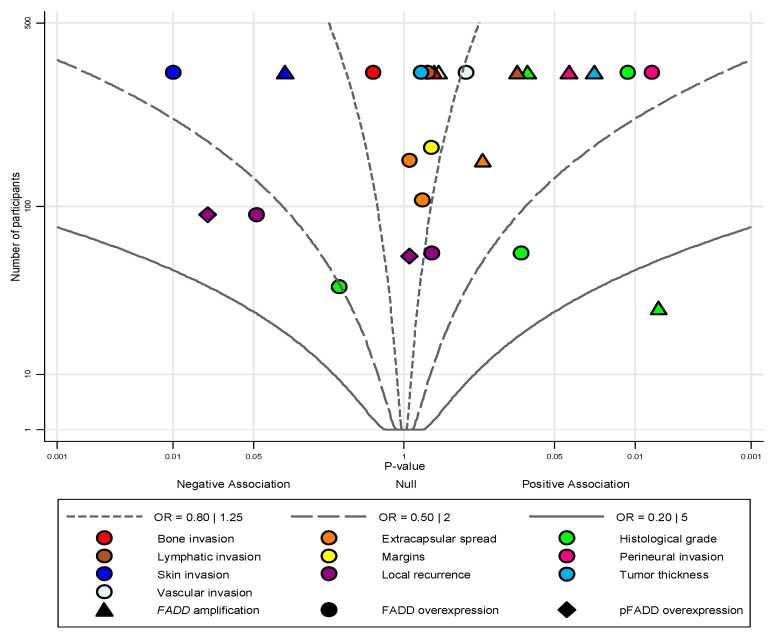
Albatross plot for studies of the association between FADD upregulation and the clinicopathological variables not included in meta-analysis: Each single study is represented by a symbol according to the alteration under study (triangle: FADD gene amplification; circle: FADD overexpression; and diamond: pFADD overexpression) and colours depicting the clinicopathological variables (see legend). Two-sided *p*-values (horizontal axis) with results separated according to positive/negative association (i.e., the observed direction of effect) were plotted against the number of subjects included within each study (vertical axis). The albatross plot allows a better interpretation of *p*-values from the variables that did not enter the meta-analysis in the context of the study sample sizes. Small studies lie toward the bottom of the plot, and large studies lie toward the top. Effect contours were drawn on the plot, showing the ranges of the magnitudes of effect for individual studies, using ORs (black continuous and intermittent lines). The effect size of contours was designed according to the Grading of Recommendations, Assessment, Development and Evaluation (GRADE) system criteria, considering an OR > 2 as large and OR > 5 as very large (see the legend). A *p*-value < 0.05 was considered significant. OR, odds ratio.

Meta-analysis was not performed for the association between FADD upregulation and the additional variables (histological grade; local recurrence; tumour thickness; margins; extracapsular spread; and bone, skin, lymphatic, vascular and perineural invasion). However, all were included in an albatross plot (Figure 4) and considered separately in the narrative synthesis. All these variables (with the exception of histological grade) were evaluated in a low number of studies, needing further investigation. Only two variables showed a significant inverse relationship with skin invasion (FADD overexpression: *p* = 0.01 [30]) and local recurrence (pFADD overexpression: *p* = 0.02 [31]; although local recurrence was defined as a time-to-event variable—estimated using HRs—it was also included in this plot in addition to clinicopathological variables, estimated with ORs). Another five variables showed a significant positive association with high histological grade (FADD amplification: *p* = 0.006 [32]; overexpression: *p* = 0.01 [30]), high tumour thickness (FADD amplification: *p* = 0.02 [30]) and perineural invasion (FADD amplification and overexpression, *p* = 0.04 and 0.007, respectively, [30]). Although the most promising results seem to derive from the histological grade variable, their results were imprecise (the only study showing a very large effect size had a small sample size [32]) and inconsistent (considerable interstudy heterogeneity degree).

### 2.6. Quantitative Evaluation (Secondary Analyses)

#### 2.6.1. Sensitivity Analysis

In general, the results were not substantively changed after the sequential repetition of meta-analyses, omitting one study in turn, and statistical significance was not lost for any study variable (Figures S27–S35, Tables S3–S11, Supplementary). The sensitivity analysis (“leave-one-out” method) confirmed the presence of an outlier and its influence on the N status parameter, underestimating the overall result (see above) (Figure 2, Figures S16 and S32, Table S8). Sensitivity analyses were also carried out to explore the potential influence of the study subsets with a lower quality or to report data from different sources (estimated from Kaplan–Meier curves and univariable or multivariable models). The general results did not substantially vary after the sequential repetition of meta-analyses, omitting each time these subsets of studies with potentially influential characteristics (Figures S36–S38, Tables S12–S14, Supplementary). This suggests that the combined estimations reported do not depend on the influence of a particular individual study or the precedent subsets of studies (with the exception of the outlier identified in the meta-analysis of N status).

#### 2.6.2. Analysis of Subgroups

The prognostic value of specific subgroups was explored for overall survival (Table 2, Figures S4–S7, Supplementary). As the type of FADD alteration under analysis was considered the main source of heterogeneity (with large prognostic differences for pFADD and FADD overexpression and gene amplification), we decided not to meta-analyse them combined in subgroups analyses. Therefore, these analyses were only performed for the FADD overexpression group because a greater number of studies and patients were investigated for this alteration. The statistically significant association was maintained for the Asian (HR = 1.66, 95% CI = 1.05–2.63, *p* = 0.03) and non-Asian subgroups (HR = 1.51, 95% CI = 1.20–1.90, *p* < 0.001) (Figure S4), for larynx SCC (HR = 1.40, 95% CI = 1.06–1.85, *p* = 0.02) and for the HNSCC mixed subgroup (HR = 1.77, 95% CI = 1.18–2.65, *p* = 0.005) (Figure S5). Although the oral and nasopharynx SCC subgroups also reached significant results, they were only analysed in two studies for this variable. Both antibodies also preserved a statistically significant association (A66-2:HR = 1.53, 95% CI = 1.18–1.97, *p* = 0.001; H181:HR = 1.54, 95% CI = 1.18–2.01, *p* = 0.002) (Figure S6) and only the subgroup including the nuclear compartment in the immunohistochemical evaluation (HR = 1.54, 95% CI = 1.25–1.91, *p* < 0.001). The cytoplasmic expression alone showed a worse prognostic value (*p* = 0.13, N.S) (Figure S7).

#### 2.6.3. Meta-Regression Analysis

Meta-regression was also performed to explore the potential effect of the study covariates sex, age, clinical stage and follow-up on the relationships of FADD overexpression on overall survival; however, no significant association was found (Table 2, Figures S8–S11, Supplementary).

#### 2.6.4. Analysis of Small-Study Effects

Visual inspection analysis of the asymmetry of the funnel plots constructed (Figures S18–S26, Supplemetary) and the statistical tests performed for the same purpose confirmed the absence of small-study effects. The prognostic and clinicopathological variables meeting the applicability conditions (i.e., enter in meta-analysis and number of studies ≥3) were overall survival (p_Egger-FADD overexpression_ = 0.167, p_Egger-pFADD overexpression_ = 0.408), disease-specific survival (p_Egger-FADD overexpression_ = 0.263), disease-free survival (p_Egger-FADD overexpression_ = 0.495), T status (p_Peters-FADD overexpression_ = 0.653), N status (p_Peters-FADD overexpression_ = 0.245, p_Peters-FADD amplification_ = 0.365) and clinical stage (p_Peters-FADD overexpression_ = 0.724, p_Peters-FADD amplification_ = 0.316). Therefore, publication bias could be ruled out for these variables.

### 2.7. Quality of Evidence

The quality of evidence was performed using GRADE [33]. According to this system, there was moderate quality of evidence for the analysis of the association between FADD overexpression and N status and low quality of evidence for FADD amplification and N status and for FADD overexpression with OS, DSS and clinical stage. The rest of the outcomes were rated as very low quality of evidence (Table S15, Supplementary).

### 2.8. Validation of Methodological Quality

The methods applied in this systematic review and meta-analysis were implemented, critically appraised and validated using AMSTAR2 [34], obtaining an overall rating of “high” (16 points) (the checklist, explanation and scoring table are included in the Supplementary).

## 3. Discussion

The results of our meta-analysis carried out on 13 studies/1923 patients demonstrate the relevance of immunohistochemical FADD overexpression as a marker of poor survival in HNSCC patients, referring to OS (HR = 1.52, 95% CI = 1.28–1.81, *p* < 0.001), DSS (HR = 2.52, 95% CI = 1.61–3.96, *p* < 0.001) and DFS (HR = 1.67, 95% CI = 1.29–2.15, *p* < 0.001). Only one study has dealt with the prognostic value of FADD gene amplification in relation to OS and DFS [30]; thus, no conclusive results can be drawn in this regard at this time. This meta-analysis has not demonstrated any prognostic value for phosphorylation of FADD in ser-194, contrary to other types of cancers, such as lung cancer and lymphomas, in which FADD phosphorylated in ser-194 is associated with a worse tumour prognosis [11,12]. From these results, it is deduced that immunohistochemistry, a simple, inexpensive and routine application technique in pathology laboratories, is the most useful tool to evaluate FADD overregulation and its influence as a prognostic marker of HNSCC. The evaluation of the immunohistochemical expression of FADD must jointly consider nuclear and cytoplasmic overexpression since these intracellular locations were exclusively those that significantly influenced the prognosis (HR = 1.54, 95% CI = 1.25–1.91, *p* < 0.001), while studies only considering FADD cytoplasmic labelling did not show a relationship with tumour prognosis. No study analyzed the prognostic value of exclusive nuclear labelling, even though it is known, as we will refer later, as important oncogenic mechanisms linked to the nuclear location of FADD, so it seems advisable to study this topic further in future researches. It should also be noted that the two monoclonal antibodies used to detect FADD overexpression (Clone A66-2 and Clone H181) yielded statistically similar results predicting a reduction in OS (*p* = 0.001 and *p* = 0.002, respectively). The results of our meta-analysis confirm what was previously reported by The Cancer Genome Atlas (TCGA) [18], which, through bioinformatics analysis of the datasets derived from 528 patients with HNSCC [21], FADD has been identified as one of the biomarkers with the highest prognostic capacity for survival; the results we present also support the findings reported by Perez-Sayans et al. (2019) [19], who performed somatic copy number alteration bioinformatics analysis in order to comprehensively describe genomic aberrations in the last extension of the HNSCC subsets from TCGA. Among a total of 3491 deregulated genes found, FADD was identified as one of the “top 5” more frequently altered HNSCC genes (*CDKN2A,* deleted in 32.03% of patients; *CDKN2B,* deleted in 28.34% of patients; *PPFIA1,* amplified in 26.02% of patients; FADD, amplified in 25.63% of patients; and *ANO1,* amplified in 25.44% of patients-) [19]. Furthermore, a comprehensive microarray data integration-based bioinformatics analysis using in silico tools (via Gene Expression Omnibus (GEO) and Array Express (EBI) public registers), reporting integrated data from microarray datasets published in public records of 15 series that included 277 HNSCC, confirmed the frequent alteration of FADD and its prognostic value [21].

The oncogenic molecular mechanisms through which FADD promotes an unfavourable evolution in HNSCCs could be related to its inhibitory capacity of transcriptional activation of the tumour suppressor gene NOTCH, acting jointly with NF-kB activating protein (NKAP) in the cell nucleus [35,36,37,38]. Through NOTCH inhibition, FADD exerts a differentiation-suppressing and proliferation-stimulating effect on tumour cells; furthermore, this pro-proliferative action of FADD is also exerted by activating the NF-kB and MAPK (Ras-Raf-MEK-Erk) pathways, both potent regulators of cyclin D1 expression, essential in the regulation of proliferative endpoints in HNSSC [9,14]. Other mechanisms that could justify the poor prognosis associated with FADD overregulation include its ability to block necroptosis, i.e., a specialized pathway of programmed necrosis [39], via caspase-8, cFLIP, and RIPK1/3 recruitment, promoting necroptosome blockade [40,41]; likewise, the complex formed by FADD, caspase-8, and RIPK1—also known as FADDosome—through the activation of NF-kB stimulates the release of proinflammatory cytokines and chemokines and the known influences of the peritumoral inflammatory infiltrate on the proliferative activity of tumour cells [42,43]; finally, it has been documented that FADD upregulation increases the metabolism of tumour cells by stimulating the glucosetransporters Gut1 [44]. Increased glucose intake by cancer cells is a well-established cancer hallmark that worsens tumour prognosis [45].

In our study, immunohistochemical overexpression of FADD was associated with a higher clinical stage (OR = 1.72, *p* = 1.17–2.51, *p* = 0.005), which was not observed with gene amplification or with ser-194 phosphorylation. FADD upregulation has also shown a significant association with N+ status, both in the analysis of gene amplification (OR = 2.30, 95% CI = 1.16–4.58, *p* = 0.02) and in the analysis of immunohistochemical protein overexpression (OR = 2.42, 95% CI = 1.84–3.18, *p* < 0.001), not finding this association for ser-194 phosphorylation (*p* = 0.98). Frequent FADD and caspase-10 mutations have also been reported in lung cancer, playing a role in the development of lymph node metastasis [46]. It has been hypothesized that the death receptor signalling pathway DR5/FADD/caspase-8 could promote the development of metastases mediated by tumour cells with acquired mechanisms of apoptosis resistance [47]. On the other hand, some publications have reported that caspase-8 [48] and FADD [49] could activate focal adhesion kinase (FAK) [50], a key molecule involved in the formation of actin-based protrusive structures [15,51], in the development of mesenchymal epithelial transition [52], in cell migration and in metastatic development [53]. The relationship of FADD upregulation with poor survival could also be due to its influence on the development of metastases, as metastases are powerful determinants of increased mortality in HNSCC.

Subgroup analysis did not show prognostic differences related to the geographic area (Asian vs. non-Asian patients) or to tumor development by anatomical sites of (oral cavity, larynx, nasopharynx, and mixed head and neck), maintaining the prognostic influence of FADD in all tumour locations (larynx: HR = 1.40, 95% CI = 1.06–1.85, *p* = 0.02; oral cavity: HR = 1.39, 95% CI = 1.03–1.87, *p* = 0.03; nasopharynx: HR = 2.27, 95% CI = 1.26–4.09, *p* = 0.006; mixed HNSCC: HR = 1.77, 95% CI = 1.18–2.65, *p* = 0.005). Finally, the relationship of FADD with important prognostic parameters (histological grade; tumour thickness; margins status; extracapsular spread; and bone, skin, lymphatic, vascular and perineural invasion) could not be meta-analysed due to the scarcity of studies offering results about them. To minimize this limitation, we made an estimation through the performance of an albatross plot, which showed an association between FADD overexpression and perineural invasion, which may constitute one more justification for the relationship between FADD and metastatic development in these tumours.

According to our qualitative evaluation using the QUIPS tool, although the studies in our meta-analysis had similar experimental and epidemiological designs, all were not conducted with same rigor. The domains harbouring a higher risk of bias were study confounding (item 5) and statistical analysis and reporting (item 6). Therefore, most potential biases were caused by the failure to consider confounding factors and by the application of inappropriate statistical analyses. According to our overall scoring system, only three studies were considered to be at high risk of bias. After applying a sensitivity analysis to assess the influence of these studies on the overall results, no substantial changes were observed. This suggests that the overall results do not depend on the influence of the subset of studies with lowest quality.

Some potential limitations of our meta-analysis should be discussed. First, a considerable grade of heterogeneity was found for the variables histological grade, local recurrence, overall survival and N status. Consequently, meta-analyses were not performed for histological grade and local recurrence (mainly due to the low number of studies analysing these parameters, insufficient to assess their sources of heterogeneity), although they were included in an albatross plot and considered separately for narrative synthesis. In relation to overall survival, a subgroup analysis by FADD alterations showed that heterogeneity was not significant after this stratification in more homogeneous subgroups. Therefore, FADD alterations (i.e., gene amplification, pFADD and FADD overexpression) were considered as the main sources of heterogeneity and subsequently analysed in an independent manner. In relation to N status, fortunately, an outlier was identified through the construction of a Galbraith plot and by performing a sensitivity analysis series. After the omission of this outlier, heterogeneity was markedly reduced, reaching nonsignificant levels, confirming again the main source of heterogeneity for this parameter. In summary, after extensive exploration of their sources, a satisfactory explanation for heterogeneity was provided, so heterogeneity should not be considered as a concerning limitation of the present work. Second, two studies did not directly report HR values in the survival analysis, although this weakness was countered by estimating HRs from the data provided by these studies, following the methods of Parmar et al. [54] and Tierney et al. [55]. Sensitivity analyses were also applied omitting this subset of studies, confirming the reliability of results. Third, an inherent limitation to some included studies may be the low amount of data available, not allowing us to conduct secondary analyses (e.g., by tobacco and alcohol consumption). Future studies should consider and measure smoking habits due to its relationship with HNSCC aetiology. Furthermore, tobacco could be a relevant confounding factor—upgrading our quality of evidence according to GRADE system [33]—modulating FADD expression levels triggered by the break at the common chromosomal fragile site, FRA11F, a mechanism involved in the amplification of the 11q13 chromosomal band [7]. Finally, all the studies were observational with a retrospective design, partially limiting the quality of evidence. Future prospective cohorts are needed to corroborate the observed associations.

Despite the above limitations, study strengths include the careful design of our systematic review and meta-analysis, conducted and validated following the robust AMSTAR2 guidelines. A comprehensive literature search strategy was performed not applying restrictions by date limits or publication language. Numerous potential subpopulations were investigated (by geographical area, anatomical site, sex, age, clinical stage, follow-up period, anti-FADD antibody and immunohistochemical pattern), reporting similarities and differences that may be useful for clinical practice and for the development of future studies. Both visual and statistical analyses confirmed the absence of small-study effects, allowing us to rule out publication bias, i.e., the tendency to publish only positive results. Finally, some meta-analyses showed powerful statistical associations (e.g., N status and FADD overexpression), as demonstrated by forest plots and sensitivity analyses.

## 4. Materials and Methods

This systematic review and meta-analysis complied with Preferred Reporting Items for Systematic Reviews and Meta-Analyses (PRISMA) and Meta-analysis Of Observational Studies in Epidemiology (MOOSE) statements [56,57], closely followed the criteria of Cochrane Prognosis Methods Group [58] and Cochrane Handbook for Systematic Reviews of Interventions [59], and was conducted and validated according to AMSTAR2 guidelines [34].

### 4.1. Protocol

In order to minimize risk of bias and to improve the transparency, precision and integrity of our systematic review and meta-analysis, a protocol on its methodology has been submitted a priori in the PROSPERO International prospective register of systematic reviews (www.crd.york.ac.uk/PROSPERO) (ID 180055 was assigned; a copy of the protocol is included in the Supplementary). The protocol followed complied with PRISMA-P statement in order to ensure rigor [60].

### 4.2. Search Strategy

We searched the PubMed, Embase, Web of Science and Scopus databases for studies published before the search date (upper limit = February 2020), with no lower date limit. Searches were conducted by combining thesaurus terms used by the databases (i.e., MeSH and EMTREE) with free terms (Table S1, Supplementary) and built to maximize sensitivity. An additional screening was performed by handsearching the reference lists of retrieved included studies and using Google Scholar. All references were managed using Mendeley v.1.19.4 (Elsevier. Amsterdam, The Netherlands); duplicate references were eliminated using this software.

### 4.3. Eligibility Criteria

Inclusion criteria: (1) original studies, without language, publication date, follow-up periods, study design, geographical area, sex or age restrictions; (2) evaluation of FADD alterations (FADD protein overexpression, FADD phosphorylation in ser-194 (pFADD) or FADD gene amplification) in human tissues from primary HNSCC; (3) analysis of the association of FADD upregulation with at least one of the following prognostic and/or clinicopathological variables: overall survival (OS), disease-specific survival (DSS), disease-free survival (DFS), local recurrence (LR), T status, N status, clinical stage and histological grade. OS was defined as the time elapsed from the date of diagnosis/surgery to the date of death by any cause. DSS was defined as the time elapsed from the date of diagnosis/surgery to the date of death by cancer. DFS was defined as the time elapsed from diagnosis/surgery to the detection of locoregional or distant recurrence or to death without recurrence. LR was defined as the time elapsed from diagnosis/surgery to the detection of recurrence at the primary tumour site. Given the lack of international consensus standards to define survival endpoints, we included studies that used the direct designation of the aforementioned terms (OS/DSS/DFS/LR) or other terms that are defined in the original studies as in the present article; and 4) when results were derived from the same study population, the reports providing more complete data were included. An interstudy overlapping population was determined by verifying the name and affiliation of authors, source of patients and recruitment period.

Exclusion criteria were (1) retractions, case reports, editorials, letters, personal opinions or comments, meeting abstracts, books, bioinformatics analyses of microarray datasets, reviews or meta-analyses; (2) in vitro or animal research; (3) no relation to HNSCC; (4) evaluations of FADD gene alterations other than gene amplification (e.g., polymorphisms) and of 11q13 chromosomal band amplification by mapping techniques, analysing the set of genes in this band without specifically discriminating those related to the FADD gene; (5) no analysis of the prognostic or clinicopathological variables of interest; and (6) lack or insufficient data for the estimation of OR/HR with 95% CI.

### 4.4. Study Selection Process

Eligibility criteria were applied independently by two authors (P.R.G. and M.A.G.M.). Any discrepancies were resolved by consensus. Articles were selected in two phases, first screening titles and abstracts for those apparently meeting inclusion criteria and then reading the full text of selected articles, excluding those that did not meet the review eligibility criteria. Evaluators were first trained and calibrated for the process of identification and selection of studies, performing a screening round (50 papers each). The inter-agreement between evaluators on study eligibility was calculated using Cohen’s kappa statistic [61]. Both reached an almost perfect agreement in the initial calibration and in the final process, obtaining initial and final kappa values of 0.852 (96% of agreement) and 1.000 (100% of agreement), respectively. Any disagreements were resolved by consensus.

### 4.5. Data Extraction

Two authors (P.R.G. and M.A.G.M.) independently extracted data from the selected articles, completing a data collection form in a standardized manner using Excel v.2015 (Microsoft. Redmond, WA). These data were additionally cross-checked by two different authors (A.A. and I.G.R.), solving discrepancies by consensus. Data were gathered on the first author, publication year, country, publication language, sample size, FADD alterations under study, methodology, the frequency of alterations, tumour location, sex and age of patients, tobacco and alcohol consumption, recruitment period, funding and potential conflict of interest, treatment modality, follow-up period and study design. In immunohistochemical studies, information was also recorded on the anti-FADD antibody, intracellular immunostaining (nuclear/cytoplasmic/mixed), cut-off point and scoring system. Finally, the data required to analyse the outcomes was also recorded for clinicopathological (T (T3/T4 vs. T1/T2) and N (N+ vs. N−) status, clinical stage (III/IV vs. I/II), histological grade (II/III vs. I)) and prognostic variables (OS, DSS, DFS and LR).

### 4.6. Evaluation of Quality and Risk of Bias of Individual Studies

Two authors (P.R.G. and M.A.G.M.) critically appraised the quality and risk of bias of studies using the Quality in Prognosis Studies (QUIPS) tool (Cochrane Prognosis Methods Group [62]). The development of this tool was based on an examination of numerous systematic reviews of prognostic studies [63], and six common areas of potential bias (domains) were identified [62]. Therefore, in the selected studies, the following six main potential bias domains were explored: (1) study participation; (2) study attrition; (3) prognostic factor measurement; (4) outcome measurement; (5) study confounding; and (6) statistical analysis/reporting. The risk of bias was considered low, moderate or high for each domain. Finally, an overall score (low/high risk of bias; based on critical domains) was assigned for each study, with the purpose of statistically analysing the influence of quality on meta-analytical results through sensitivity analyses (see below). Prognostic factor measurement (item 3) and study confound (item 5) were considered critical domains. This approach was based on the scoring system of recent high standards guidelines for systematic reviews (i.e., AMSTAR2 [34]). The critical domains (i.e., relevant weak points that most frequently harbour a high risk of bias) were chosen based on prestigious guidelines for meta-analysis of observational studies (Newcastle–Ottawa Scale [64]), reported recommendations for prognostic biomarkers in cancer (REEMARK guidelines [65]) and our results from previous meta-analyses on the prognostic implications of biomarkers in head and neck carcinogenesis [25,26,66,67]. Domains were independently evaluated in each individual study by both authors, who recorded the particularities and potential biases observed. Discrepancies were also resolved by consensus.

### 4.7. Evaluation of Quality of Evidence

Two authors (P.R.G. and M.A.G.M.) evaluated quality of evidence using the “Grading of Recommendations Assessment, Development and Evaluation” GRADE system [33]. According to GRADE, the quality of evidence is classified in one of four levels: very low, low, moderate or high. As recommended, an initial baseline overall quality of evidence (i.e., “low” for observational studies) was assigned to each outcome. Then, that overall quality rating was “downgraded” or “upgraded” based on the following domains: risk of bias, inconsistency, indirectness, imprecision, publication bias and magnitude of effect size [33] (the full explanation is listed in the Supplementary).

### 4.8. Validation of Methodological Quality

Two independent authors (P.R.G. and M.A.G.M.) critically appraised and validated the methodology followed in this systematic review and meta-analysis using “A MeaSurement Tool to Assess systematic Reviews” AMSTAR2 checklist [34], created as an instrument to develop, evaluate and validate high quality systematic reviews through 16 items (the 16-items checklist is listed in the Supplementary). An overall rating is obtained based on weaknesses in critical domains (i.e., items: 2, 4, 7, 9, 11, 13 and 15) and noncritical domains. The overall confidence on the methodology of the systematic review is rated in one of four level: “high”, “moderate”, “low” and “critically low” (the full explanation is also listed in the Supplementary

### 4.9. Statistical Analysis

FADD amplification was considered as “positive” or “negative” in agreement with the methodology assumed by the authors of each study. If different gene gain levels were reported, “high gain” was considered as amplification. FADD and/or pFADD expression was considered “high” or “low” according to the cut-off values provided by the authors of each study. When each individual study analysed more than one alteration (i.e., amplification and overexpression), both data were gathered and analysed separately. Independent meta-analyses were performed to evaluate the potential impact of FADD upregulation (FADD amplification, FADD and/or pFADD overexpression) on clinicopathological (T status, N status and clinical stage) and prognostic variables (OS, DSS and DFS). Although the meta-analyses of histological grade and local recurrence were also planned in our protocol, they were not performed due to a considerable grade of heterogeneity and low number of studies. Additional parameters were rarely reported (extracapsular spread; tumour thickness; margins; and bone, skin, lymphatic, vascular and perineural invasion), being gathered but not meta-analysed to avoid unjustified random deviations from our protocol and to preserve the internal validity of this research. Nevertheless, due to their potential prognostic implications, an albatross plot was constructed to graphically represent them [68], allowing an approximate examination of their underlying magnitudes of effect.

Odds ratios (OR) with their corresponding 95% confidence intervals (CI) were estimated and used for the meta-analyses of the clinicopathological variables. Hazard ratios (HR) and 95% CI were used for the prognostic variables due to their time-to-event nature [55]. When authors published these measures, they were directly extracted from the original articles. If HRs with 95% CI were not explicitly provided by the authors, they were calculated using the methods described by Parmar et al. [54] and by Tierney and colleagues [55]. When a study only reported survival curves, we extracted the data from Kaplan–Meier curves with Engauge Digitizer 4.1 software (open-source digitizing software developed by M. Mitchell). Only one study reported raw data for overall survival (considering it as a dichotomous variable). OR and 95% CI were calculated for this study; nevertheless, the estimate was very high (OR = 30, 95% CI = 3.15–285.70) and the event rate was not rare (>5%). Therefore, this ratio metric was not assumed as an approximation of the HR and not meta-analysed for overall survival to avoid an overestimated overall effect size [69,70]. All other studies reported HRs, or they were estimated through Kaplan–Meier curves (Table S2). When HRs were determined in both univariable and multivariable models, data were extracted from the multivariable model, which reflects a greater adjustment for potentially confounding factors.

In the meta-analyses, the individual studies were combined by association measure to obtain a single estimate. Pooled estimates were obtained using the inverse-variance method under a random-effects model (based on the DerSimonian and Laird method), which accounts for the possibility that there are different underlying results among study subpopulations (i.e., differences among head and neck subsites, linked to geographic areas or related to the inherent heterogeneity of the wide range of experimental methods). Forest plots were constructed to graphically represent the overall effect and for subsequent visual inspection analysis (*p* < 0.05 was considered significant). Heterogeneity between studies was checked applying the χ²-based Cochran’s Q test (given its low statistical power, *p* < 0.10 was considered significant) and quantified using Higgins I2 statistic (values of 50–75% were interpreted as moderate-to-high degree of inconsistency across the studies), which estimates what proportion of the variance in observed effects reflects variation in true effects, rather than sampling error [71,72].

Pre-planned stratified meta-analyses (by FADD alterations) were performed to identify potential sources of heterogeneity in all variables (see the protocol). Furthermore, additional subgroup analyses (by geographical area, HNSCC subsite, anti-FADD antibody and immunohistochemical pattern) and univariable meta-regression analyses were conducted to examine the relationships of FADD upregulation with overall survival and to explore the potential effect of study covariates (sex, age, clinical stage and follow-up period) [73]. Considering the low number of studies with data available for meta-regression analyses, the *p*-values were calculated using a permutation test based on a Monte Carlo simulation [74]. To obtain sufficient precision, the number of permutations was 10,000 [75]. For illustrative purposes, weighted bubble plots were also constructed to graphically represent the fitted meta-regression lines. Suspecting that a particular study [28] was the source responsible for heterogeneity in the meta-analysis of N status, an ad hoc Galbraith plot was constructed to identify the potential outlier [76]. In addition, sensitivity analyses were carried out to test the reliability of meta-analytical results and to explore the influence of each individual study on the final estimations for each meta-analysis performed [77]. For this, the meta-analyses were repeated sequentially, omitting one study at a time (“leave-one-out” method). Additional sensitivity analyses were performed, omitting subsets of studies (by low quality/high risk of bias and source of data, i.e., obtained from Kaplan–Meier curves and univariable or multivariable models) to test the robustness of results against potentially influential characteristics [59].

Finally, funnel plots were constructed [78] and the Egger [79] and Peters [80] regression tests were used to evaluate small-study effects, such as publication bias. The first, the gold standard test for funnel plot asymmetry, performed a linear regression of the effect estimates on their standard errors, weighting by 1/(variance of the effect estimate), and was applied for prognostic variables (pEgger < 0.10 was considered significant). The Peters test regresses the effect estimate on 1/n with weights dh/n, where n is the total sample size, d is the number experiencing the event and h is the number not experiencing the event. It was applied for clinicopathological variables (pPeters < 0.10) due to its better performance for dichotomous outcomes measured as odds ratios (preserving the statistical power of the Egger test, with a lower type 1 error rate, i.e., false positives). Stata version 14.1 (Stata Corp, College Station, TX, USA) was employed for all tests, manually typing the commands syntax (PRG) [81].

## 5. Conclusions

In conclusion, our systematic review and meta-analysis demonstrates that FADD upregulation, especially with regard to immunohistochemical protein overexpression, behaves as a powerful prognostic predictor in HNSCC as a consequence of its association with higher clinical stage and N positive status. All of this suggests including the routine immunohistochemical analysis of FADD overexpression in the prognostic evaluation of HNSCC. Further research on the utility of FADD as a therapeutic target is also advised, as although early work on the subject reported promising results [20,22,23,24], there are still few published studies.

## Figures and Tables

**Table 1:** Summarized characteristics of reviewed studies.

Total	13 Studies
Year of publication	2007–2017
Number of patients	
Total	1923 patients
Sample size, range	30–339 patients
FADD alterations analysed *	
FADD overexpression	11 studies (1727 patients)
pFADD overexpression	3 studies (285 patients)
FADD gene amplification	4 studies (563 patients)
Affected sites	
Oral SCC	5 studies (747 patients)
Laryngeal SCC	4 studies (332 patients)
Nasopharyngeal SCC	1 study (248 patients)
HNSCC mixed	3 studies (596 patients)
Study design	
Retrospective cohort	13 studies
Geographical region	
Europe	7 studies (663 patients)
Asia	4 studies (687 patients)
North America	2 studies (419 patients)

*—More than one alteration was analysed per study.

**Table 2:** Meta-analyses of prognostic and clinicopathological significance of FADD upregulation inNSCC.

					Pooled Data		Heterogeneity		
Meta-Analyses	No. of Studies	No. of Patients	Stat. Model	Wt	ES (95% CI)	*p-Value*	*P_het_*	*I^2^* (%)	*Appendix ^a^*
**SURVIVAL PARAMETERS**									
**Overall survival**									
All ^b^	7 *	1198 *	REM	D-L	HR = 1.45 (1.16–1.81)	0.001	<0.001	74.3	FigureS3, p10
Subgroup analysis by alteration ^c^									
FADD amplification	1	339	──	──	HR = 1.53 (1.10–2.12)	0.01	──	──	FigureS3, p10
pFADD overexpression	3	285	REM	D-L	HR = 1.14 (0.82–1.56)	0.44	0.15	46.9	FigureS3, p10
FADD overexpression	7	1196	REM	D-L	HR = 1.52 (1.28–1.81)	<0.001	0.50	0.0	FigureS3, p10
Subgroup analysis by geographical area (FADD overexpression group) ^c^									
Asian	2	587	REM	D-L	HR = 1.66 (1.05–2.63)	0.03	0.15	52.9	FigureS4, p11
Non-Asian	5	609	REM	D-L	HR = 1.51 (1.20–1.90)	<0.001	0.52	0.0	FigureS4, p11
Subgroup analysis by affected site (FADD overexpression group) ^c^									
LSCC	3	290	REM	D-L	HR = 1.40 (1.06–1.85)	0.02	0.39	0.0	FigureS5, p12
OSCC	1	339	──	──	HR = 1.39 (1.03–1.87)	0.03	──	──	FigureS5, p12
NPSCC	1	248	──	──	HR = 2.27 (1.26–4.09)	0.006	──	──	FigureS5, p12
HNSCC mixed	2	319	REM	D-L	HR = 1.77 (1.18–2.65)	0.005	0.50	0.0	FigureS5, p12
Subgroup analysis by anti-FADD antibody (FADD overexpression group) ^c^									
A66-2	4	512	REM	D-L	HR = 1.53 (1.18–1.97)	0.001	0.36	7.3	FigureS6, p13
H181	3	684	REM	D-L	HR = 1.54 (1.18–2.01)	0.002	0.34	6.9	FigureS6, p13
Subgroup analysis by immunohistochemical pattern (FADD overexpression group) ^c^									
Cytoplasmic	2	189	REM	D-L	HR = 1.58 (0.87–2.88)	0.13	0.65	0.0	FigureS7, p14
Nuclear and cytoplasmic	5	1007	REM	D-L	HR = 1.54 (1.25–1.91)	<0.001	0.27	22.5	FigureS7, p14
Univariable meta-regression ^d^									
Sex (% of males)	7	1196	random-effects meta-regression		Coef = −0.003 (−0.027 to 0.021)	0.729 ± 0.004 ^e^	──	──	FigureS8, p15
Age (mean age of patients)	7	1196	random-effects meta-regression		Coef = −0.008 (−0.052 to 0.035)	0.704 ±0.004 ^e^	──	──	FigureS9, p16
Stage (% of stage-III/IV patients)	7	1196	random-effects meta-regression		Coef = 0.003 (−0.007 to 0.013)	0.558 ± 0.005 ^e^	──	──	FigureS10, p17
Follow up period (months)	7	1196	random-effects meta-regression		Coef = −0.001 (−0.005 to 0.004)	0.769 ± 0.004 ^e^	──	──	FigureS11, p18
**Disease-specific survival**									
All ^b^	3 *	422 *	REM	D-L	HR=2.63 (1.76–3.92)	<0.001	0.56	0.0	FigureS12, p19
Subgroup analysis by alteration ^c^									
pFADD overexpression	1	133	──	──	HR = 3.05 (1.29–7.22)	0.01	──	──	FigureS12, p19
FADD overexpression	3	422	REM	D-L	HR = 2.52 (1.61–3.96)	<0.001	0.73	0.0	FigureS12, p19
**Disease-free survival**									
All ^b^	3 *	658 *	REM	D-L	HR = 1.57 (1.28–1.94)	<0.001	0.53	0.0	FigureS13, p20
Subgroup analysis by alteration ^c^									
FADD amplification	1	339	──	──	HR = 1.39 (0.96–2.02)	0.08	──	──	FigureS13, p20
FADD overexpression	3	658	REM	D-L	HR = 1.67 (1.29–2.15)	<0.001	0.45	0.0	FigureS13, p20
**Local recurrence**									
All ^b^	2 *	152 *	REM	D-L	Data not pooled	0.21	0.03	67.3	──
Subgroup analysis by alteration ^c^									
pFADD overexpression	2	152	REM	D-L	Data not pooled	0.41	0.02	81.6	Manuscript, Figure 4
FADD overexpression	2	150	REM	D-L	Data not pooled	0.45	0.06	70.7	
**CLINICO-PATHOLOGICAL CHARACTERISTICS**									
**T status**									
All ^b^	3*	727 *	REM	D-L	OR = 0.83 (0.63–1.08)	0.17	0.57	0.0	FigureS14, p21
Subgroup analysis by alteration ^c^									
FADD amplification	1	339	──	──	OR = 1.07 (0.63–1.82)	0.79	──	──	FigureS14, p21
FADD overexpression	3	727	REM	D-L	OR = 0.76 (0.55–1.03)	0.08	0.67	0.0	FigureS14, p21
**N status**									
All ^b^	10*	1649 *	REM	D-L	OR = 2.07 (1.47–2.91)	<0.001	0.008	55.2	
Subgroup analysis by alteration ^c^									
FADD amplification	3	533	REM	D-L	OR = 2.30 (1.16–4.58)	0.02	0.18	41.1	Manuscript, Figure 2
pFADD overexpression	1	59	──	──	OR = 1.02 (0.27–3.83)	0.98	──	──	
FADD overexpression	9	1483	REM	D-L	OR = 2.07 (1.34–3.20)	0.001	0.005	63.3	
Sensitivity analysis									
All ^f^	9 *	1609 *	REM	D-L	OR = 2.36 (1.85–3.00)	<0.001	0.27	17.9	FigureS16, p23
Sensitivity analysis stratified by alteration ^f^									
FADD amplification	3	533	REM	D-L	OR = 2.30 (1.16–4.58)	0.02	0.18	41.1	FigureS16, p23
pFADD overexpression	1	59	──	──	OR = 1.02 (0.27–3.83)	0.98	──	──	FigureS16, p23
FADD overexpression	8	1443	REM	D-L	OR = 2.42 (1.84–3.18)	<0.001	0.30	16.3	FigureS16, p23
**Clinical stage**									
All ^b^	7 *	812 *	REM	D-L	OR = 1.74 (1.26–2.41)	0.001	0.44	0.0	FigureS17, p24
Subgroup analysis by alteration ^c^									
FADD amplification	3	224	REM	D-L	OR = 1.92 (0.73–5.06)	0.18	0.20	38.3	FigureS17, p24
pFADD overexpression	1	59	──	──	OR = 1.02 (0.27–3.83)	0.98	──	──	FigureS17, p24
FADD overexpression	5	616	REM	D-L	OR = 1.72 (1.17–2.51)	0.005	0.44	0.0	FigureS17, p24
**Histological grade**									
All ^b^	3 *	439 *	REM	D-L	Data not pooled	0.02	0.10	48.7	──
Subgroup analysis by alteration ^c^									
FADD amplification	2	369	REM	D-L	Data not pooled	0.17	0.04	76.2	Manuscript, Figure 4
FADD overexpression	3	439	REM	D-L	Data not pooled	0.22	0.18	41.0	

Abbreviations: Stat., statistical; Wt, method of weighting; ES, estimation; CI, confidence intervals; REM, random-effects model; D-L, DerSimonian and Laird method; LSCC, laryngeal squamous cell carcinoma; OSCC, oral squamous cell carcinoma; NPSCC, nasopharyngeal squamous cell carcinoma; HNSCC, head and neck squamous cell carcinomas; OR, odds ratio; HR, hazard ratio. * More than one alteration was analyzed per study. ^a^—More information in the appendix, ^b^—Prognosis meta-analyses, ^c^—Prognosis meta-analyses (Subgroup analyses), ^d^—Effect of study covariates on overall survival and FADD overexpression among patients with HNSCC, ^e^—*p*-value ± standard error after 10,000 permutations based on Montecarlo simulations, ^f^—“Leave-one-out” method. Haili et al. 2010—identified as an outlier and main source of heterogeneity- was omitted from N status meta-analysis (see also Figure 3).

## Supplementary Materials

The following are available online at https://www.mdpi.com/2072-6694/12/9/2393/s1. **Figure S1**. Graphic representation of the risk of bias (QUIPS tool), **Figure S2**. Porcentual quantification of the risk of bias, **Figure S3**. Forest plot graphically representing the stratified analysis of the association between FADD alterations and overall survival in patients with HNSCC, **Figure S4**. Forest plot graphically representing the subgroup meta-analysis by geographical area (Asian vs Non Asian) of the association between FADD overexpression and overall survival in patients with HNSCC, **Figure S5**. Forest plot graphically representing the subgroup meta-analysis by affected site (larynx, oral cavity, nasopharynx and head and neck mixed squamous cell carcinomas) of the association between FADD overexpression and overall survival in patients with HNSCC, **Figure S6**. Forest plot graphically representing the subgroup meta-analysis by anti-FADD antibody (A66-2 vs. H181 clones) of the association between FADD overexpression and overall survival in patients with HNSCC, **Figure S7**. Forest plot graphically representing the subgroup meta-analysis by immunostaining pattern (mixed nuclear-cytoplasmic vs. cytoplasmic) of the association between FADD overexpression and overall survival in patients with HNSCC, **Figure S8**. Bubble plot graphically representing the univariable meta-regression analysis of the potential effect of sex (% of males) on the association between FADD and overall survival among patients with HNSCC, **Figure S9**. Bubble plot graphically representing the univariable meta-regression analysis of the potential effect of age (mean age of patients, expressed in years) on the association between FADD and overall survival among patients with HNSCC, **Figure S10**. Bubble plot graphically representing the univariable meta-regression analysis of the potential effect of clinical stage (% of stage III/IV patients) on the association between FADD and overall survival among patients with HNSCC, **Figure S11**. Bubble plot graphically representing the univariable meta-regression analysis of the potential effect of follow up period (expressed In months) on the association between FADD and overall survival among patients with HNSCC, **Figure S12**. Forest plot graphically representing the stratified analysis of the association between FADD alterations and disease-specific survival in patients with HNSCC, **Figure S13**. Forest plot graphically representing the stratified analysis of the association between FADD alterations and disease-free survival in patients with HNSCC, **Figure S14**. Forest plot graphically representing the stratified analysis of the association between FADD alterations and T status (T3/T4 vs. T1/T2) in patients with HNSCC, **Figure S15**. Galbraith plot of the association between FADD alterations and N status in HNSCC, constructed to examine the contributions of individual studies to the heterogeneity metrics and identify outliers. It contains additional information, allowing the identification of studies (data not showed in Figure 3 due to graphic purposes), **Figure S16**. Forest plot graphically representing the stratified analysis of the association between FADD alterations and N status (positive vs. negative) in patients with HNSCC, with the omission of the outlier (Haili et al. 2010) identified in the previous figure (S14), **Figure S17**. Forest plot graphically representing the stratified analysis of the association between FADD alterations and clinical stage (III/IV vs. II) in patients with HNSCC, **Figure S18**. A funnel plot of estimated logHR against its standard error, graphically representing the analysis of small-study effects on Overall Survival in HNSCC, **Figure S19**. A funnel plot of estimated logHR against its standard error, graphically representing the analysis of small-study effects on Overall Survival in HNSCC, **Figure S20**. A funnel plot of estimated logHR against its standard error, graphically representing the analysis of small-study effects on Disease-Specific Survival in HNSCC. **Figure S21**. A funnel plot of estimated logHR against its standard error, graphically representing the analysis of small-study effects on Disease-Free Survival in HNSCC, **Figure S22**. A funnel plot of estimated logOR against its standard error, graphically representing the analysis of small-study effects on T status in HNSCC, **Figure S23**. A funnel plot of estimated logOR against its standard error, graphically representing the analysis of small-study effects on N status in HNSCC, **Figure S24**. A funnel plot of estimated logOR against its standard error, graphically representing the analysis of small-study effects on N status in HNSCC, **Figure S25**. A funnel plot of estimated logOR against its standard error, graphically representing the analysis of small-study effects on Clinical Stage in HNSCC, **Figure S26**. A funnel plot of estimated logOR against its standard error, graphically representing the analysis of small-study effects on Clinical Stage in HNSCC, **Table S1**. Search strategy for each database, number of results, and execution date, **Table S2**. Characteristics of the analyzed studies, **Table S3**. Sensitivity analysis of the studies pooled in the meta-analysis on the association between FADD overexpression and overall survival in HNSCC, **Figure S27**. Interval plot graphically representing the sensitivity analysis from Table S3, **Table S4**. Sensitivity analysis of the studies pooled in the meta-analysis on the association between pFADD overexpression and overall survival in HNSCC, **Figure S28**. Interval plot graphically representing the sensitivity analysis from Table S4, **Table S5**. Sensitivity analysis of the studies pooled in the meta-analysis on the association between FADD overexpression and disease-specific survival in HNSCC, **Figure S29**. Interval plot graphically representing the sensitivity analysis from Table S5, **Table S6**. Sensitivity analysis of the studies pooled in the meta-analysis on the association between FADD overexpression and disease-free survival in HNSCC, **Figure S30**. Interval plot graphically representing the sensitivity analysis from Table S6, **Table S7**. Sensitivity analysis of the studies pooled in the meta-analysis on the association between FADD overexpression and T status in HNSCC, **Figure S31**. Interval plot graphically representing the sensitivity analysis from Table S7, **Table S8**. Sensitivity analysis of the studies pooled in the meta-analysis on the association between FADD overexpression and N status in HNSCC, **Figure S32**. Interval plot graphically representing the sensitivity analysis from Table S8, **Table S9**. Sensitivity analysis of the studies pooled in the meta-analysis on the association between FADD amplification and N status in HNSCC, **Figure S33**. Interval plot graphically representing the sensitivity analysis from Table S9, **Table S10**. Sensitivity analysis of the studies pooled in the meta-analysis on the association between FADD overexpression and clinical stage in HNSCC, **Figure S34**. Interval plot graphically representing the sensitivity analysis from Table S10, **Table S11**. Sensitivity analysis of the studies pooled in the meta-analysis on the association between FADD amplification and clinical stage in HNSCC, **Figure S35**. Interval plot graphically representing the sensitivity analysis from Table S11, **Table S12**. Sensitivity analysis of the study subsets by overall quality, pooled in the meta-analysis on the association between FADD overexpression and overall survival in HNSCC, **Figure S36**. Interval plot graphically representing the sensitivity analysis from Table S12, **Table S13**. Sensitivity analysis of the study subsets by source of data, pooled in the meta-analysis on the association between FADD overexpression and overall survival in HNSCC, **Figure S37**. Interval plot graphically representing the sensitivity analysis from Table S13, **Table S14**. Sensitivity analysis of the study subsets by overall quality, pooled in the meta-analysis on the association between FADD overexpression and N status in HNSCC, **Figure S38**. Interval plot graphically representing the sensitivity analysis from Table S14, **Table S15**. Grading of Recommendations Assessment, Development and Evaluation (GRADE) system, **List S1**. Explanation of risk of bias across studies for each domain, **List S2**. AMSTAR2 checklist, **Table S16**. AMSTAR2 scoring system, **List S3**. Records screened and excluded according to titles and abstracts, **List S4**. Full-text articles excluded, **List S5**. List of studies included in this systematic review and meta-analysis, and copy of **protocol**.
