# Supplementary material for: Prognostic and Clinicopathological Significance of FADD Upregulation in Head and Neck Squamous Cell Carcinoma: A Systematic Review and Meta-Analysis
Source: Cancers (Basel). 2020 Aug 24;12(9):2393. doi: 10.3390/cancers12092393 (PMC7563729; doi:10.3390/cancers12092393)
Supplement: Supplementary file 1 [file cancers-12-02393-s001.pdf]

Appendix to the manuscript

Prognostic and clinicopathological significance of FADD upregulation in head and neck squamous cell carcinoma: a systematic review and meta-analysis.

Miguel Ángel González-Moles[1,2,*], Ángela Ayén[3], Isábel González-Ruiz[1,2], Teresa de Porras-Carrique[1,2], Lucía González-Ruiz[4], Isábel Ruiz-Ávila[5] and Pablo Ramos-García[1,2,*]

1-School of Dentistry, University of Granada, Granada, Spain

2-Instituto de Investigación Biosanitaria ibs.GRANADA, Granada, Spain

3-Dermatology Service, San Cecilio Hospital Complex, Granada, Spain.

4-Dermatology Service, Ciudad Real General University Hospital, Ciudad Real, Spain.

5-Pathology Service, San Cecilio Hospital Complex, Granada, Spain.

*Correspondence: magonzal@ugr.es (MAGM); pramos@correo.ugr.es (PRG).

***Corresponding Authors:**

Miguel Ángel González Moles and Pablo Ramos-García

Oral Medicine Department, School of Dentistry, University of Granada,

Granada, Paseo de Cartuja s/n, 18071 Granada, Spain.

Tel.:+34958243804; fax:+34958240908.E-mail: magonzal@ugr.es

Table of contents

1. Search strategy. Table S1. 4

2. Characteristics of analyzed studies. Table S2 5

3. Qualitative analysis 7

3.1 Graphic representation of the risk of bias (QUIPS tool). Figure S1. 7

3.2 Porcentual quantification of the risk of bias. Figure S2....................................8

3.3 Explanation of risk of bias across studies for each domain. List S1..................9

4. Meta-analysis on the association between FADD and overall survival.......................10

4.1 FADD alterations and overall survival 10

4.2 FADD overexpression and overall survival by geographical area...................11

4.3 FADD overexpression and overall survival by affected site 12

4.4 FADD overexpression and overall survival by anti-FADD antibody.............13

4.5 FADD overexpression and overall survival by immunostaining pattern.........14

4.6 Effect of sex on the association between FADD and overall survival............15

4.7 Effect of age on the association between FADD and overall survival 16

4.8 Effect of clinical stage on the association between FADD and overall survival..17

4.9 Effect of follow up period on the association between FADD and overall survival..18

5. Meta-analysis on the association between FADD and disease-specific survival........19

6. Meta-analysis on the association between FADD and disease-free survival..............20

7. Meta-analysis on the association between FADD and T status 21

8. Meta-analysis on the association between FADD and N status.................................22

8.1 Galbraith plot of the association between FADD alterations and N status......22

8.2 Sensitivity analysis with the omission of the identified outlier. 23

9. Meta-analysis on the association between FADD and clinical stage 24

10. Analysis of small-study effects...25

10.1 FADD overexpression and Overall Survival in HNSCC..............................25

10.2 pFADD overexpression and Overall Survival in HNSCC............................26

10.3 FADD overexpression and Disease-Specific Survival in HNSCC................27

10.4 FADD overexpression and Disease-Free Survival in HNSCC......................28

10.5 FADD overexpression and T status in HNSCC..29

10.6 FADD overexpression and N status in HNSCC 30

10.7 *FADD* amplification and N status in HNSCC 31

10.8 FADD overexpression and Clinical Stage in HNSCC..................................32

10.9 *FADD* amplification and Clinical Stage in HNSCC.....................................33

11. Sensitivity analysis (“leave-one-out method” by individual studies)........................34

11.1 FADD overexpression and Overall Survival in HNSCC 34
11.2 pFADD overexpression and Overall Survival in HNSCC 35
11.3 FADD overexpression and Disease-Specific Survival in HNSCC 36
11.4 FADD overexpression and Disease-Free Survival in HNSCC 37
11.5 FADD overexpression and T status in HNSCC 38
11.6 FADD overexpression and N status in HNSCC 39
11.7 *FADD* amplification and N status in HNSCC 40
11.8 FADD overexpression and Clinical Stage in HNSCC 41
11.9 *FADD* amplification and Clinical Stage in HNSCC 42
12. Sensitivity analysis (by study subsets). 43
12.1 FADD overexpression and Overall Survival in HNSCC by Quality 43
12.2 FADD overexpression and Overall Survival in HNSCC by Source of Data .44
12.3 FADD overexpression and N status in HNSCC by Quality 45
13. Evaluation of quality of evidence. Table S15. GRADE system...............................46
14. Validation of methodological quality 47
14.1 List S2. AMSTAR2 checklist 47
14.2 Table S16. AMSTAR2 scoring system 51
15. List of excluded studies with reasons 52
15.1 List S3. Records screened and excluded according to titles and abstracts 52
15.2 List S4. Full-text articles excluded...66
14. List of studies included in this systematic review and meta-analysis. List S5..........68
15. Protocol...69

1. Search strategy

Table S1. Search strategy for each database, number of results, and execution date.

Database	Query	Results	Upper limit
PubMed	("Fas-Associated Death Domain Protein"[MH] OR ("Fas-Associated Death Domain Protein"[ALL]) OR "FADD"[ALL]) AND (“head”[MH] OR “head”[ALL] OR “neck”[MH] OR “neck”[ALL] OR "mouth"[MH] OR "mouth"[ALL] OR "oral"[ALL] OR "pharynx”[MH] OR pharyn*[ALL] OR oropharyn*[ALL] OR nasopharyn*[ALL] OR hypopharyn*[ALL] OR "larynx”[MH] OR laryn*[ALL] OR "nose”[MH] OR “nose”[ALL] OR “nasal”[ALL]) AND ("carcinoma, squamous cell"[MH] OR "carcinoma"[ALL] OR "Head and Neck Neoplasms"[MH] OR neoplas*[ALL] OR “cancer”[ALL])	96	February, 2020
Embase	('Fas associated death domain protein'/exp OR ('fass' N4 'associated' N4 'death' N4 'domain') OR 'FADD') AND ('head'/exp OR 'head' OR 'neck'/exp OR 'neck' OR 'mouth'/exp OR 'mouth' OR 'oral' OR 'pharynx'/exp OR 'pharyn*' OR 'oropharyn*' OR 'nasopharyn*' OR 'hypopharyn*' OR 'larynx'/exp OR 'laryn*' OR 'nose'/exp OR 'nose' OR 'nasal') AND ('squamous cell carcinoma'/exp OR 'carcinoma' OR 'head and neck cancer'/exp OR 'cancer' OR 'neoplas*')	184	February, 2020
Web of Science	TS=(FADD OR “Fas associated death domain protein”) AND TS=(head OR neck OR mouth OR oral OR pharyn* OR oropharyn* OR nasopharyn* OR hypopharyn* OR laryn* OR nose OR nasal) AND TS=(“squamous cell carcinoma” OR neoplas* or cancer)	149	February, 2020
Scopus	TITLE-ABS-KEY((“FADD” OR “Fas associated death domain protein”) AND ("head" OR "neck" OR "mouth" OR "oral" OR "pharyn*" OR "oropharyn*" OR "nasopharyn*" OR "hypopharyn*" OR "laryn*" OR "nose" OR "nasal") AND (“squamous cell carcinoma” OR “neoplas*” or “cancer”))	119	February, 2020
Total			548

Table S2. Characteristics of the analyzed studies (n=13).

Study	Year	Country	Language	Alteration analyzed (sample size)	Tumor site (n, subsites)	Sex, M/F	Age. years	Tobacco	Alcohol	recruitmen t period	therapy	Follow-up, months	Study design	Outcome (estimate)	Data Source	Funding	Conflict of interest	Methods	Anti-FADD antibody	IHC pattern	IHQ Cutoff, %	FADD+, %
Gibcus *et al.*	2007	Multicentric:<br>Netherlands<br>(3 Centers)<br>Spain<br>(1 Center)	English	FADD overexpression<br>(140 cases; missing 34)<br>pFADD overexpression<br>(133 cases; missing 41)	Larynx<br>(NR)	135/ 39	Median: 64<br>Range: 34-89	NR	NR	NR	Sx, Rt	<60	O,R,L	OS<br>DSS<br>(HR)	survival analysis: univariate Cox regression analysis	Government and University grants	NR	IHQ	(o)<br>clone A66-2, monoclonal 1:100<br><br>(p)<br>Ser194 pFADD Polyclonal 1:25	mixed nuclear cytoplasmic	NR	(o)<br>44.28<br><br>(p)<br>45.86
Haili et al.	2010	China	Chinese	FADD overexpression<br>(40 cases)	Larynx<br>(glottis 30, supraglottis 10)	37/3	Mean: 61.7<br>Range: 41-85	NR	NR	2004-2005	Sx	≤60	O,R,L	OS<br>N status<br>stage<br>histological grade<br>(OR)	Clinocpathological characteristics: raw	NR	NR	IHQ	NR, Polyclonal	cytoplasmic	NR	80.00
Paprinjumrune et al.	2010	Japan	English	*FADD* amplification<br>(30 cases)<br>FADD overexpression<br>(60 cases)	Oral cavity<br>(tongue60)	(a)<br>20/10<br><br>(o)<br>39/21	(a)<br>Mean: 55.56<br>Range: 26-84<br><br>(o)<br>Mean: 56.78<br>Range: 23-84	NR	NR	NR	Sx	<60	O,R,L	DDS<br>(HR)<br>N status<br>Stage<br>histological grade<br>(OR)	survival analysis: estimated from Kaplan-Meier curves<br><br>Clinocpathological characteristics: raw	NR	NR	(a)<br>rt-PCR<br><br>(o)<br>IHQ	NR, Monoclonal 1:40	mixed nuclear cytoplasmic	29.2<br>(based on tertiles)	(a):<br>43.33<br><br>(o):<br>66.66
Schrijvers et al.	2012	Multicentric:<br>Netherlands<br>(>10 Centers)	English	FADD overexpression<br>(92 cases)<br>pFADD overexpression<br>(92 cases)	Larynx<br>(glottis 92)	82/10	Median: 65.0<br><br>Range: 40-86	+65<br>-4<br>missing 23	+50<br>-25<br>missing 17	1997-2004	Rt	60	O,R,L	OS<br>LR<br>(HR)	survival analysis: LR:multivariate Cox regression Analysis (adjousted for FADD and pFADD overexpression)<br><br>OS:univariate Cox regression analysis	NR	None	IHQ	(o):<br>clone A66-2, monoclonal 1:100<br><br>(p):<br>NR, 1:25	(o)<br>cytoplasmic<br><br>(p)<br>nuclear	(o)<br>NR<br><br>(p)<br>71.0<br>(based on curve analyses)	(o)<br>22.82<br><br>(p):<br>67.39
Rasamny et al.	2012	USA	English	FADD overexpression<br>(222 cases)	HNSCC<br>(Oral cavity 82, Orophatynx 33, Hypopharynx16 Supraglottis 57, Glottis 27, sinus 7)	168/ 54	Mean: 57.9	+185<br>-37	+124<br>-72	1990-1999	Sx	<240	O,R,L	OS<br>DSS<br>DFS<br>(HR)<br>T status<br>N status<br>stage<br>(OR)	survival analysis: multivariate Cox regression Analysis<br>(OS adjusted for alcohol, site, N status, stage and cyclinD1;<br>DDS: alcohol, site, TN status, stage and cyclinD1;<br>DFS: site, Nstatus, stage and cyclinD1)<br><br>Clinicopathological characteristics: raw	None	None	IHQ	clone A66-2, monoclonal 1:100	mixed nuclear cytoplasmic	based on intensity	26.12
Fan et al.	2013	USA	English	FADD Overexpression<br>(197 cases, missing 7; survival data derived from N+ patients, 97 cases)	HNSCC<br>(oral cavity, larynx, oropharynx, n=NR)	130/ 74	NR	NR	NR	NR	Sx	≤96	O,R,L	OS<br>DFS<br>(HR)<br>N status<br>(OR)	Survival analysis: estimated from Kaplan-Meier curves<br><br>Clinocpathological characteristics: raw	Government and University grants	None	IHQ	clone H181, polyclonal 1:500	cytoplasmic	N status: ≥10<br><br>OS, DFS:<br>based on cell count and intensity	Nstatus:<br>87.31<br><br>OS,DFS:<br>52.57

Table S2. Characteristics of analyzed studies (n=13) (continuation).

Pattje et al.	2013	Netherlands	English	FADD Overexpression<br>(177 cases)	HNSCC<br>(Oral cavity 100,<br>Oropharynx 30,<br>Hypopharynx 8,<br>Larynx 39)	114/<br>63	Median:<br>59<br>Range:<br>24-90	NR	NR	1993-2003	Sx, Rt	36-60	O,R,L	T status<br>N status<br>margins<br>extracapsu-lar spread<br>(OR)	Clinocpathological characteristics: raw	NR	NR	IHQ	clone<br>clone A66-2,<br>monoclonal<br>1:100	cytoplasmic	based on intensity	55.93
Ribeiro *et al.*	2014	Portugal	English	*FADD* amplification<br>(30 cases)	Oral cavity<br>(Tongue 13,<br>floor of mouth12,<br>buccal mucosa 4,<br>palate 1)	26/4	Median:<br>63<br>Range:<br>37-84	+20<br>-10	NR	2010-2012	Sx, Rt,Ct	NR	O,R	stage<br>(OR)	Clinocpathological characteristics: raw	University grants	Two authors served as manufacturers of MLPA probemixes	MLPA	_	_	_	66.66
Li *et al.*	2014	China	English	FADD overexpression<br>(248 cases)	Nasopharynx<br>248	187/<br>61	Median:<br>47.5<br>Range:<br>17-83	NR	NR	NR	Sx	10-125	O,R,L	OS<br>(HR)<br>N status<br>stage<br>(OR)	Survival analysis: multivariate Cox regression Analysis<br>(OS adjusted for N status, stage, histologic grade, age, sex and treatment)<br><br>Clinocpathological characteristics: raw	Government grants	None	IHQ	clone H181,<br>polyclonal<br>1:500	mixed<br>nuclear<br>cytoplasmic	based on cell count and intensity	63.7
van Kempen et al.	2015	Netherlands	English	*FADD*<br>amplification<br>(164 cases)	Oral cavity 164	98/66	Median:<br>61.0<br>Range:<br>23-90	+83<br>-81	+88<br>-76	1997-2011	Sx, Rt	≤60	O,R,L	N status<br>stage<br>(OR)	Clinocpathological characteristics: raw	Government grants	Two authors served as developpers of the MLPA system	MLPA	_	_	_	12.19
Chien et al.	2016	Taiwan	English	*FADD* amplification<br>(339 cases)<br>FADD overexpression<br>(339 cases)	Oral cavity<br>(Buccal mucosa 155,<br>Tongue 104,<br>other 80)	339/0	Mean:<br>50.38<br>Range:<br>26-82	+290<br>-49	+181<br>-158	1999-2011	Sx,Rt,Ct	<168	O,R,L	OS<br>DFS<br>(HR)<br>T status<br>N status<br>histological grade<br>extracapsu-lar spread<br>skin, bone, perineural, vascular and lymphatic invasion.<br>(ORs)	Survival analysis: univariate Cox regression analysis<br><br>Clinocpathological characteristics: raw	Government and Hospital grants	None	(a)<br>rt-PCR<br><br>(o)<br>IHQ	clone H181,<br>polyclonal<br>1:500	mixed<br>nuclear<br>cytoplasmic	based on Intensity	(a)<br>20.35<br><br>(o)<br>56.93
Watchers et al.	2017	Netherlands	English	FADD overexpression<br>(58 cases; missing 2)<br>pFADD overexpression<br>(60 cases)	Larynx<br>(supraglottis 60)	44/16	Median:<br>62.0<br>Range:<br>33-96	NR	NR	1990-2008	Rt	≤169	O, R, L	OS<br>LR<br>N status<br>Stage<br>Sex	survival analysis: univariate Cox regression analysis<br><br>Clinocpathological characteristics: raw	Government grants	None	IHQ	(o)<br>clone A66-2,<br>monoclonal<br>1:100<br><br>(p)<br>Ser194 pFADD,<br>Polyclonal<br>1:25	(o)<br>mixed<br>nuclear<br>cytoplasmic<br><br>(p)<br>nuclear	(o):<br>Intensity<br><br>(p)<br>based on cell count and intensity	(o)<br>33.33<br><br>(p)<br>71.66
Noorlag et al.	2017	Netherlands	English	FADD overexpression<br>(154 cases; missing 4)	Oral cavity<br>(tongue 93,<br>floor of mouth 65)	97/61	Mean:<br>62.8<br>Range:<br>23-90	+83<br>-75	+76<br>-82	2004-2010	Sx, Rt,Ct	12	O,R,L	N status	Clinocpathological characteristics: raw	Government grants	NR	IHQ	clone556402,<br>monoclonal<br>1:100	cytoplasmic	based on intensity	19.48

IHC, immunohistochemistry; rt-PCR, real-time polymerase chain reaction; CGH, comparative genomic hybridization; MLPA, multiplex ligation probe amplification; Sx, surgery; Rt, radiotherapy; CT, chemotherapy; (a), *FADD* gene amplification; (o) FADD overexpression; (p) pFADD overexpression; N/A, not available.

3. Qualitative analysis

3.1. Figure S1. Graphic representation of the risk of bias (QUIPS tool)

	Study participation	Study attrition	Prognosis factor measurement*	Outcome measurement	Study confounding*	Statistical analysis and reporting	Overall risk of bias
Gibcus et al. 2007	−	+	?	+	−	+	+
Haili et al. 2010	?	+	−	+	−	−	−
Paprinjumrune et al. 2010	?	+	+	+	−	−	+
Schrijvers et al. 2012	+	+	−	+	?	?	+
Rasamny et al. 2012	+	+	+	+	+	−	+
Fan et al. 2013	−	?	−	−	−	−	−
Pattje et al. 2013	?	+	+	−	−	?	+
Ribeiro et al. 2014	?	+	+	+	−	−	+
Li et al. 2014	?	+	+	+	−	+	+
van Kempen et al. 2015	+	+	+	+	−	?	+
Chien et al. 2016	+	+	+	−	?	+	+
Watchers et al. 2017	?	+	+	+	−	+	+
Noorlag et al. 2017	+	+	−	−	−	?	−

A summary table of review authors' judgements for each risk of bias domain for each study using Quality in Prognosis Studies (QUIPS) tool. An overall rating was obtained based on weaknesses in critical domains(*). Green, low risk of potential bias; yellow, moderate; red, high.

3.2. Figure S2. Porcentual quantification of the risk of bias

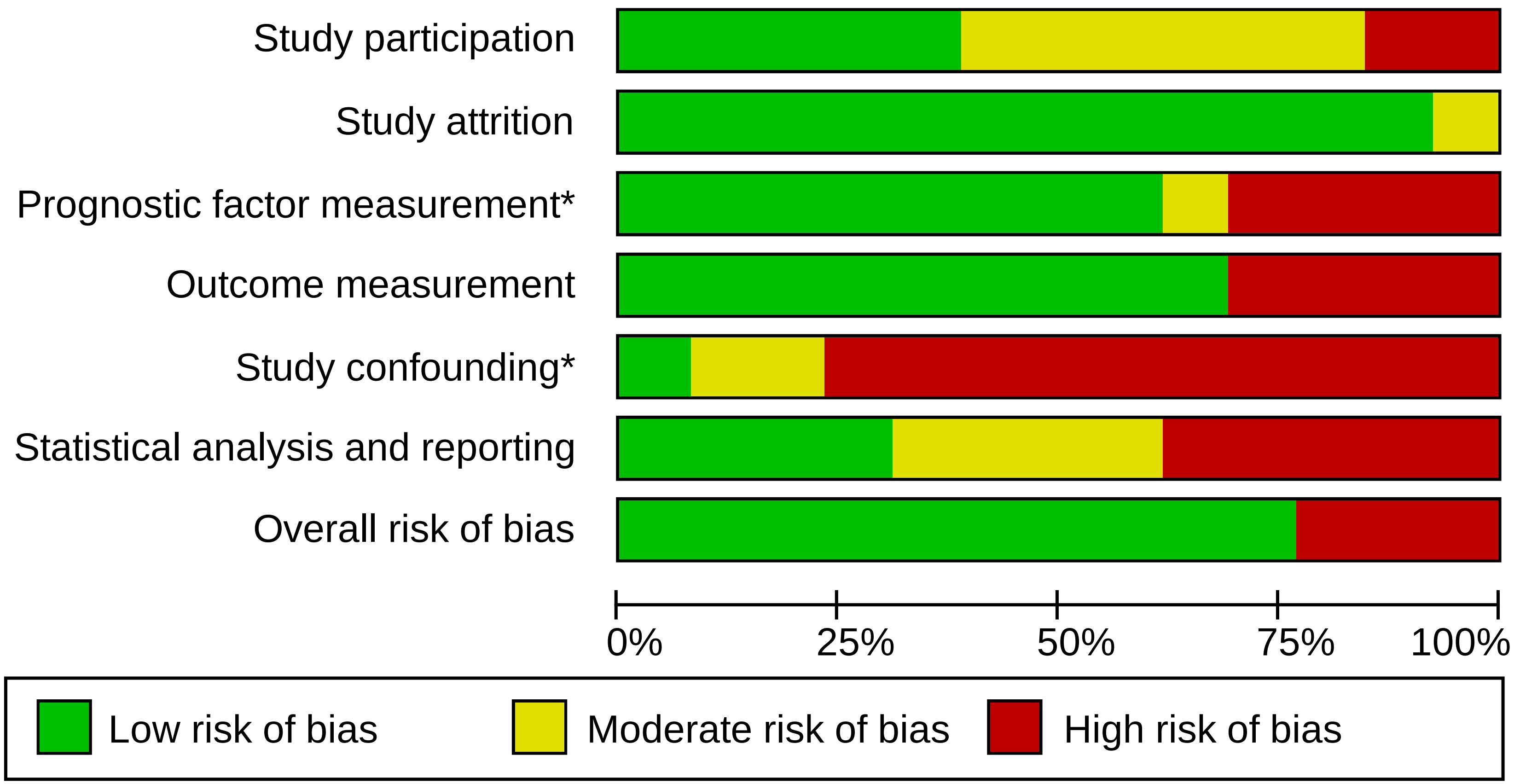

A plot of the porcentual quantification of the risk of potential bias across studies for each risk of bias domain, assessed with the QUIPS tool. An overall rating was obtained based on weaknesses in critical domains(*). Green, low risk of potential bias; yellow, moderate; red, high.

3.3 List S1. Explanation of risk of bias across studies for each domain

Domain 1 - Study participation. The risk of this bias was high in 15.39% of the reviewed studies, moderate in 46.15%, and low in 35.46% (Fig. S2,3). The potential biases were the inadequate description of patient characteristics (age, sex, anatomical subsites, tobacco and/or alcohol consumption).

Domain 2 - Study attrition. The risk of this bias was moderate in 7.69% of the studies, and low in 92.31% (Fig. S2,3), most frequently due to insufficient or no data on the follow-up period. Although the overall score for this domain was considered optimal for all studies, none described any attempt to gather information on patients who dropped out or on their characteristics. This is essential to ensure that the participants not lost to follow-up adequately represent the study sample.

Domain 3 - Prognostic factor measurement. The bias risk was high in 30.77% of the studies, moderate in 7.69%, and low in 61.54% (Fig. S2,3). The most frequent biases were due to insufficient information on the immunohistochemical technique or on scoring system for measuring FADD expression levels. More important limitations such as FADD expression not measured in a similar way for all outcomes or the application of inappropriate cutoff points (e.g., use of optimized cutoff points based on data analysis, which can introduce strong biases in research studies) were also present.

Domain 4 - Outcome measurement. The risk of this bias was high in 30.77% of the studies, and low in 62.23% (Fig. S2,3). The potential biases were the non-definition of survival endpoints (essential due to the lack of international consensus on survival endpoints) and the failure to report the classification system used (e.g., the AJCC/UICC TNM staging systems and/or editions, subject to periodic changes).

Domain 5 - Study confounding. The risk of this bias was high in 76.92% of the studies, moderate in 15.39%, and low in 7.69% (Fig. S2,3), finding the highest risk of potential bias in this domain. The most frequent potential biases were the failure to consider confounders in the study design or to measure all potential confounders (essentially tobacco and alcohol use). Although in some cases multivariable analyses were performed adjusting for potential confounders, no study provided *a priori* clear definitions of these factors considered or subsequently discussed them or the biological mechanisms by which they might influence the impact of FADD alterations on study variables.

Domain 6 - Statistical analysis and reporting. The risk of this bias was considered to be high in 38.46% of the reviewed articles, moderate in 30.77%, and low in 30.77% (Fig. S2,3). The most frequent biases were selective outcome reporting and the lack of essential information to determine whether analyses (e.g., Kaplan-Meier curves, confidence intervals and number of events in each arm). Most serious potential biases detected were inappropriate statistical analyses, erroneous data reporting and the use of odds ratios for analyzing time-to-event outcomes.

Overall quality. It was acceptable and varied among the domains under consideration. According to the scoring system - based on weaknesses in critical domains- only 3 studies harbored a higher overall risk of bias (Haili et al. 2010; Fan et al. 2013; van Kempen et al. 2015) (Fig. S2,3).

4. Meta-analysis on the association between FADD and overall survival

4.1 FADD alterations and overall survival

Figure S3. Forest plot graphically representing the stratified analysis of the association between FADD alterations and overall survival in patients with HNSCC.

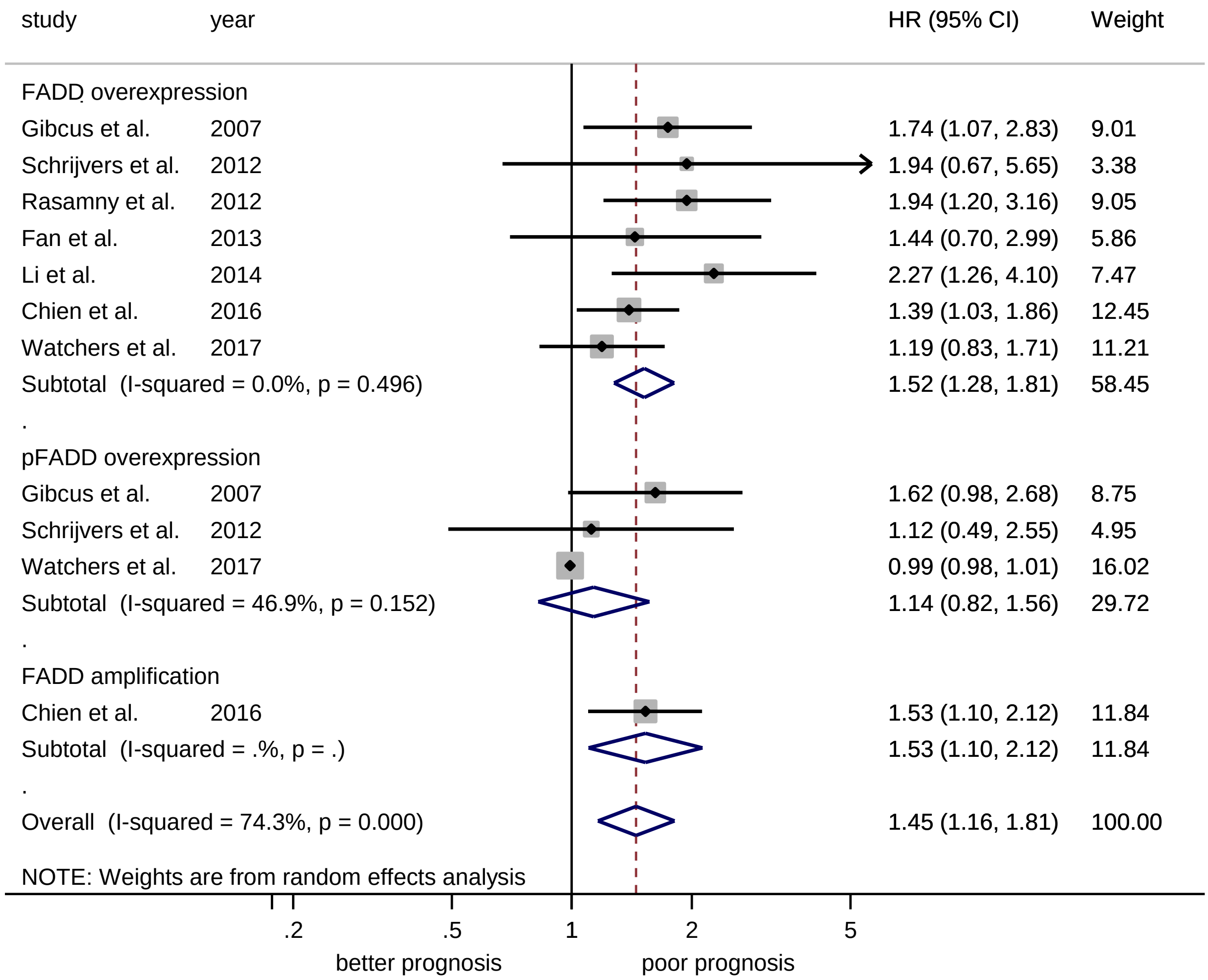

HNSCC, head and neck squamous cell carcinoma; HR, hazard ratio; CI, confidence intervals. Random-effects model, inverse-variance weighting (based on the DerSimonian and Laird method). A HR > 1 suggests that FADD alterations are associated with poor overall survival. Diamonds indicate the pooled HRs with their corresponding 95% CIs.

4.2 FADD overexpression and overall survival by geographical area

Figure S4. Forest plot graphically representing the subgroup meta-analysis by geographical area (Asian vs Non Asian) of the association between FADD overexpression and overall survival in patients with HNSCC.

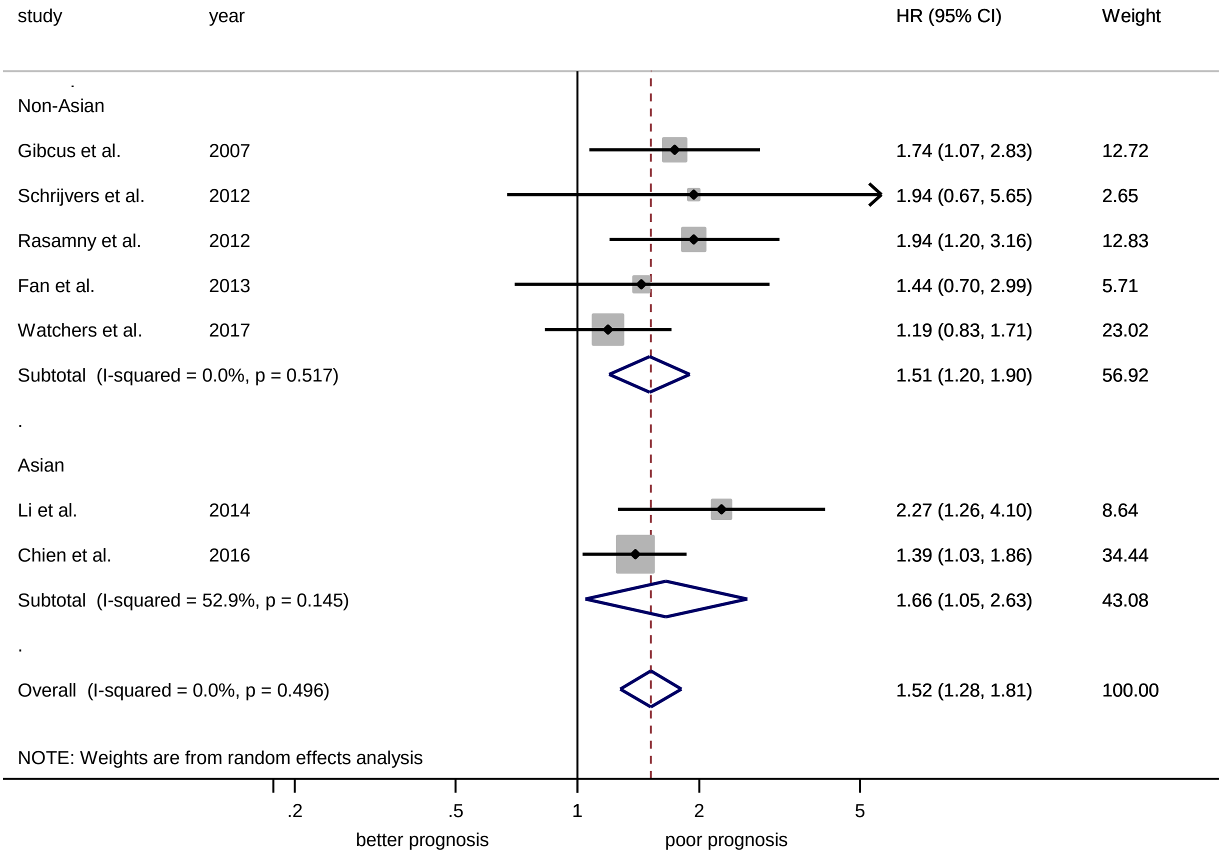

HNSCC, head and neck squamous cell carcinoma; HR, hazard ratio; CI, confidence intervals. Random-effects model, inverse-variance weighting (based on the DerSimonian and Laird method). A HR > 1 suggests that FADD alterations are associated with poor overall survival. Diamonds indicate the pooled HRs with their corresponding 95% CIs.

4.3 FADD overexpression and overall survival by affected site

Figure S5. Forest plot graphically representing the subgroup meta-analysis by affected site (larynx, oral cavity, nasopharynx and head and neck mixed squamous cell carcinomas) of the association between FADD overexpression and overall survival in patients with HNSCC.

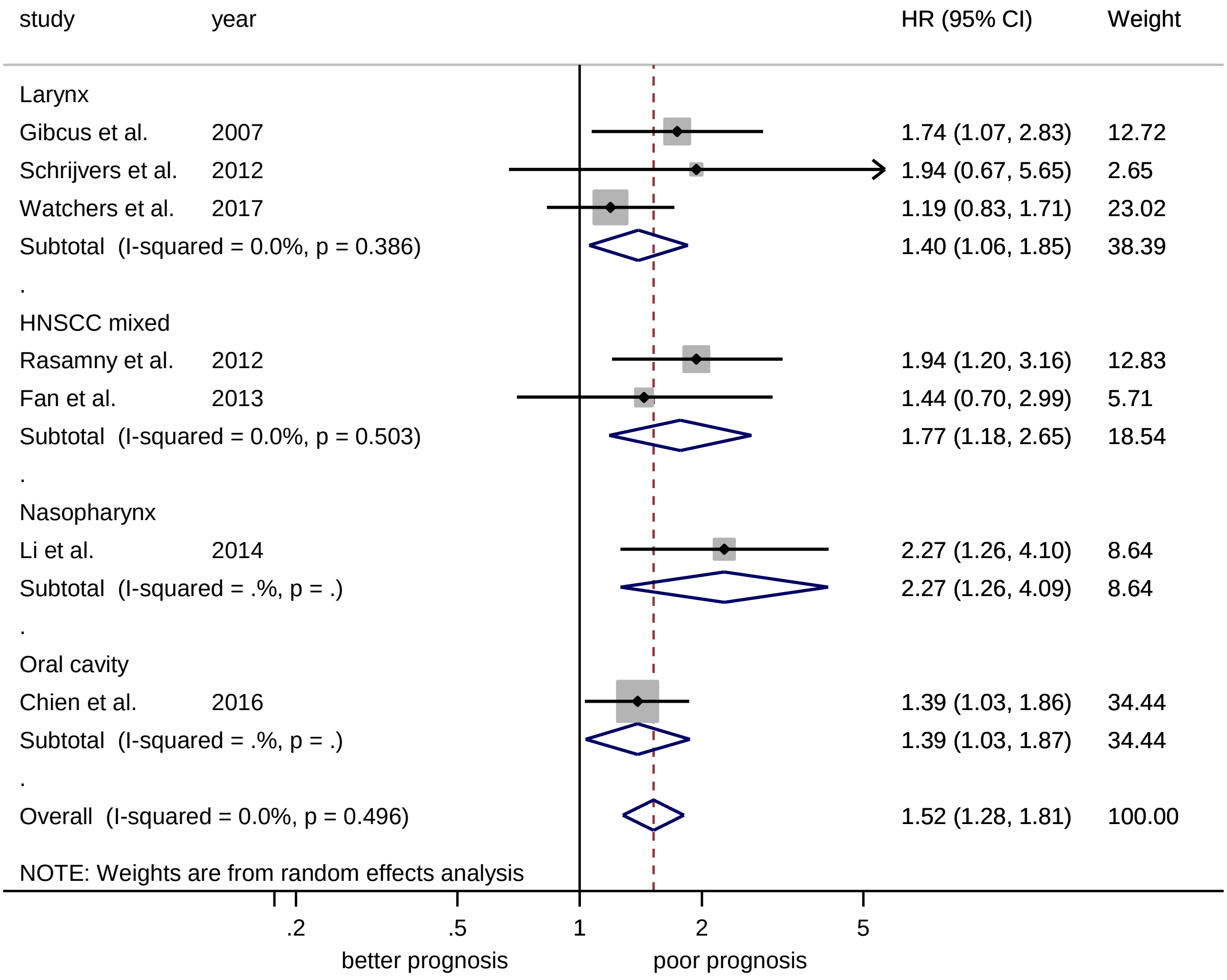

HNSCC, head and neck squamous cell carcinoma; HR, hazard ratio; CI, confidence intervals. Random-effects model, inverse-variance weighting (based on the DerSimonian and Laird method). A HR > 1 suggests that FADD alterations are associated with poor overall survival. Diamonds indicate the pooled HRs with their corresponding 95% CIs.

4.4 FADD overexpression and overall survival by anti-FADD antibody

Figure S6. Forest plot graphically representing the subgroup meta-analysis by anti-FADD antibody (A66-2 *vs.* H181 clones) of the association between FADD overexpression and overall survival in patients with HNSCC.

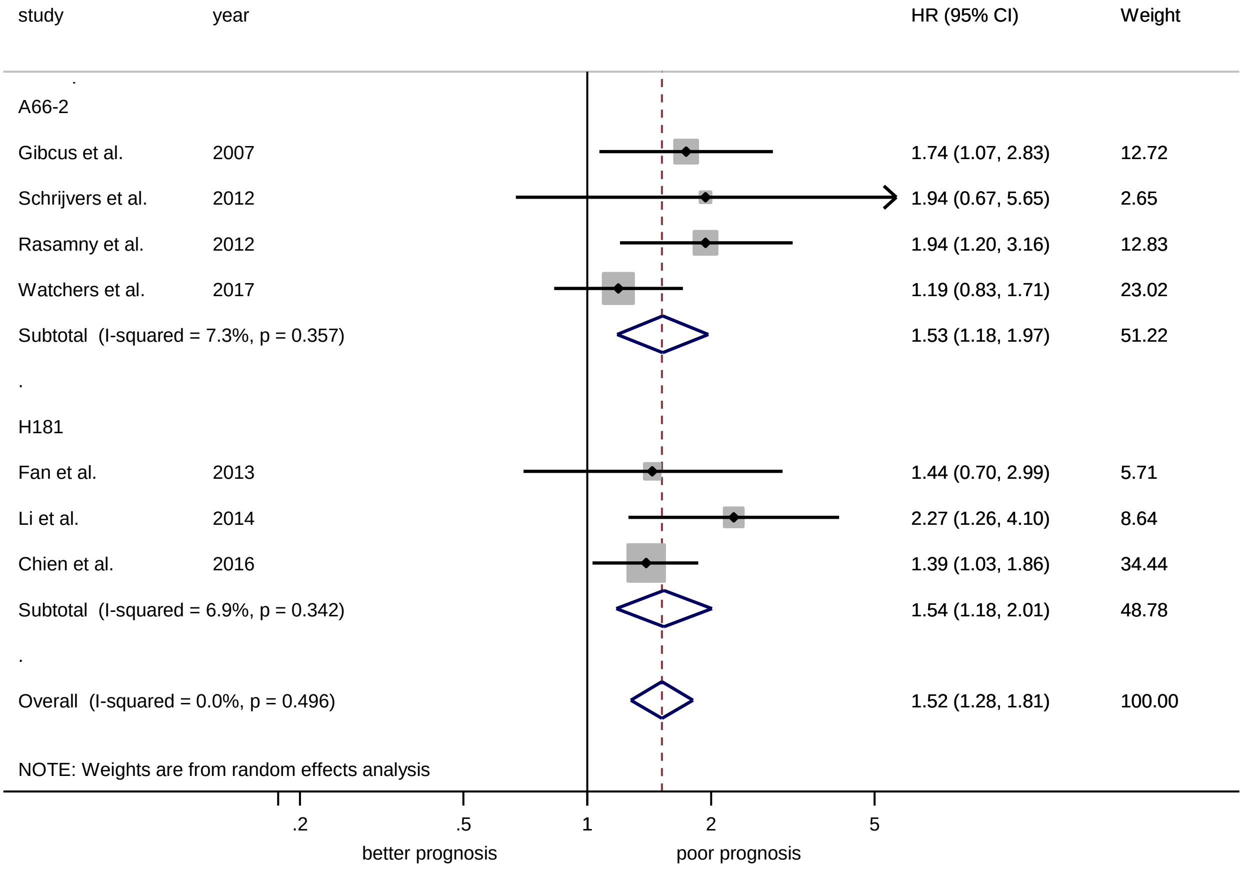

HNSCC, head and neck squamous cell carcinoma; HR, hazard ratio; CI, confidence intervals. Random-effects model, inverse-variance weighting (based on the DerSimonian and Laird method). A HR > 1 suggests that FADD alterations are associated with poor overall survival. Diamonds indicate the pooled HRs with their corresponding 95% CIs.

4.5 FADD overexpression and overall survival by immunostaining pattern

Figure S7. Forest plot graphically representing the subgroup meta-analysis by immunostaining pattern (mixed nuclear-cytoplasmic *vs.* cytoplasmic) of the association between FADD overexpression and overall survival in patients with HNSCC.

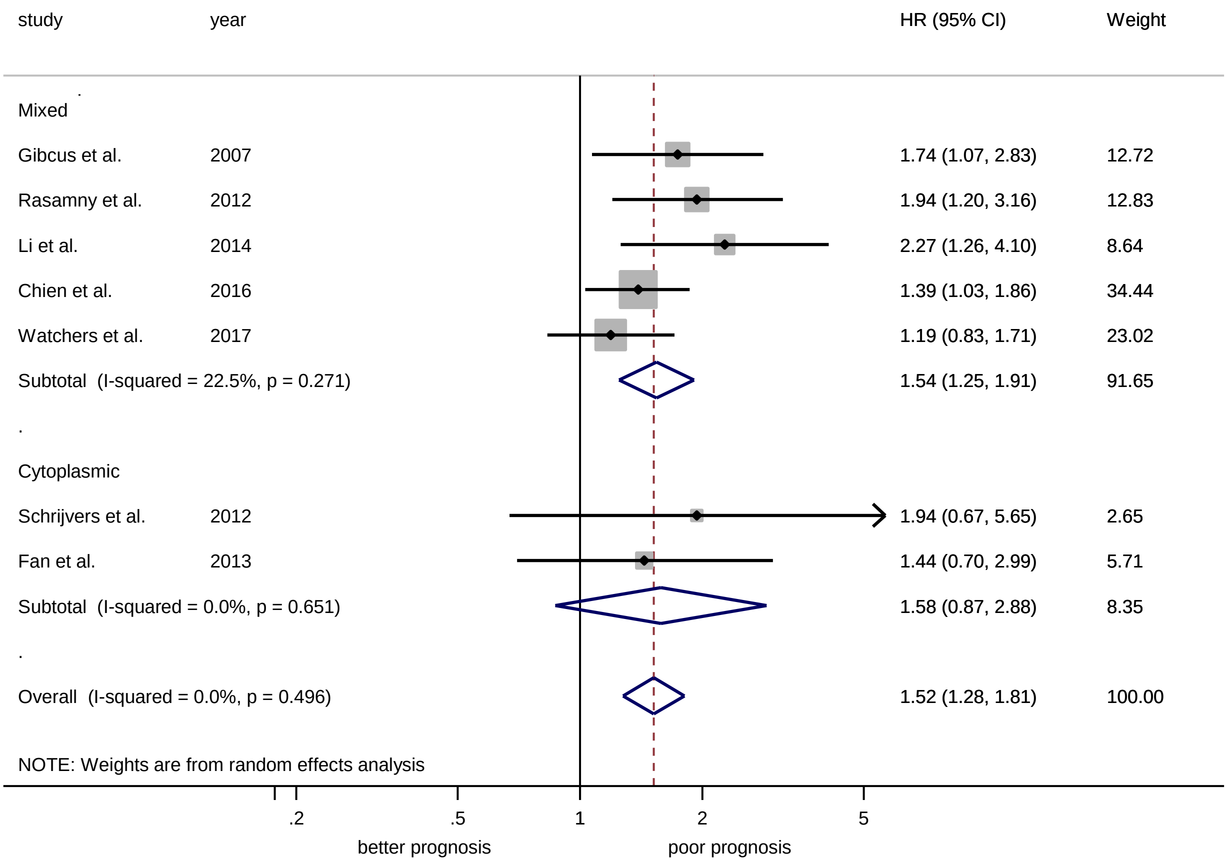

HNSCC, head and neck squamous cell carcinoma; HR, hazard ratio; CI, confidence intervals. Random-effects model, inverse-variance weighting (based on the DerSimonian and Laird method). A HR > 1 suggests that FADD alterations are associated with poor overall survival. Diamonds indicate the pooled HRs with their corresponding 95% CIs.

4.6 Effect of sex on the association between FADD and overall survival.

Figure S8. Bubble plot graphically representing the univariable meta-regression analysis of the potential effect of sex (% of males) on the association between FADD and overall survival among patients with HNSCC.

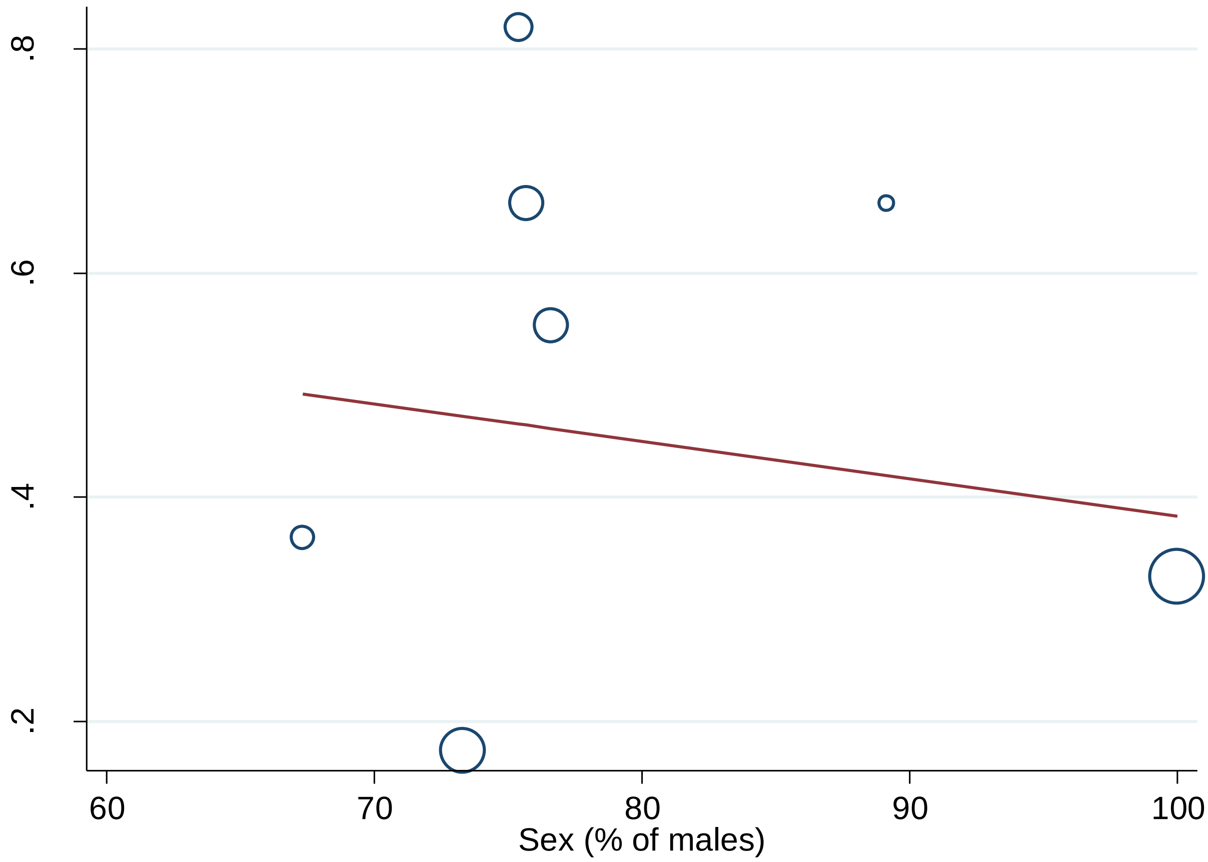

HNSCC, head and neck squamous cell carcinoma; HR, hazard ratio. The red line exhibits the fitted regression line together with blue circles representing the estimates from each individual study, sized according to the precision of each estimate (the inverse of its within-study variance).

4.7 Effect of age on the association between FADD and overall survival

Figure S9. Bubble plot graphically representing the univariable meta-regression analysis of the potential effect of age (mean age of patients, expressed in years) on the association between FADD and overall survival among patients with HNSCC.

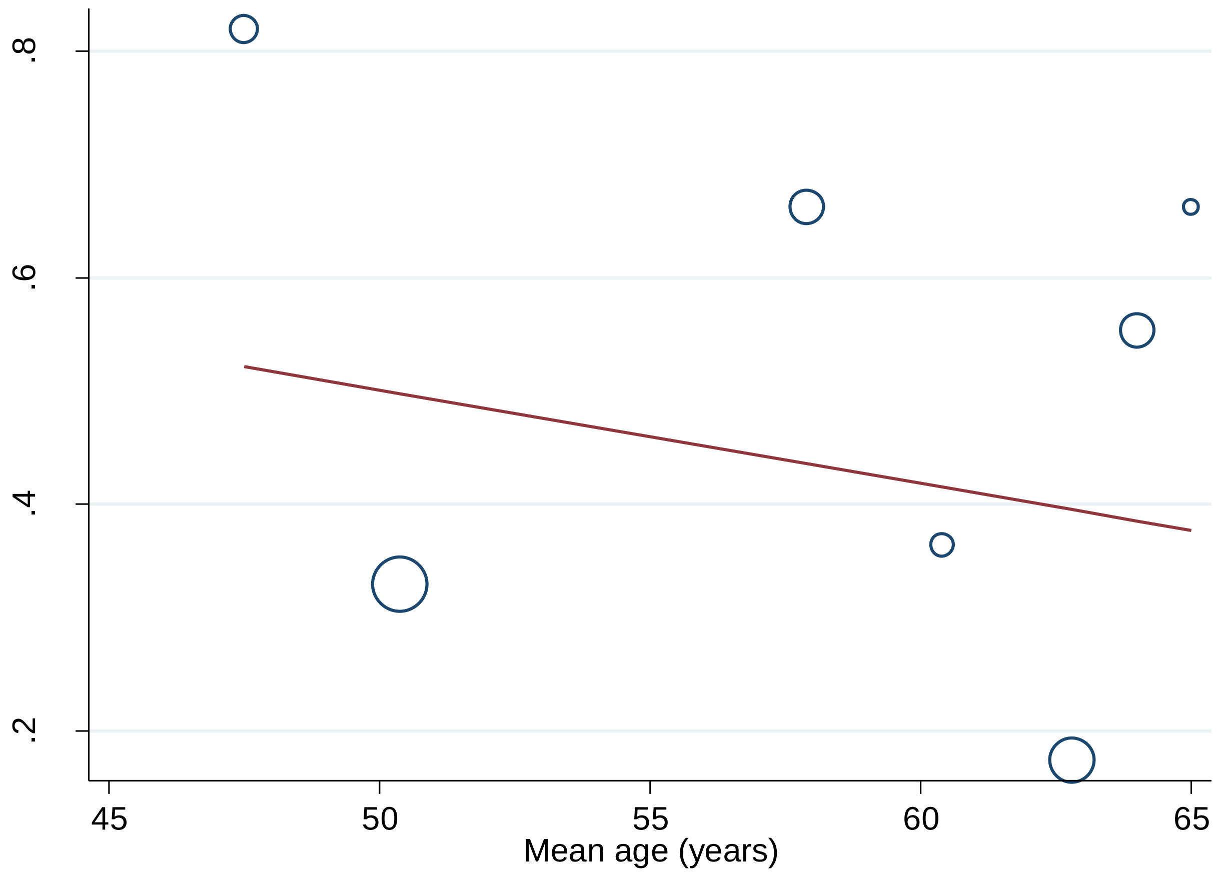

HNSCC, head and neck squamous cell carcinoma; HR, hazard ratio. The red line exhibits the fitted regression line together with blue circles representing the estimates from each individual study, sized according to the precision of each estimate (the inverse of its within-study variance).

4.8 Effect of clinical stage on the association between FADD and overall survival

Figure S10. Bubble plot graphically representing the univariable meta-regression analysis of the potential effect of clinical stage (% of stage III/IV patients) on the association between FADD and overall survival among patients with HNSCC.

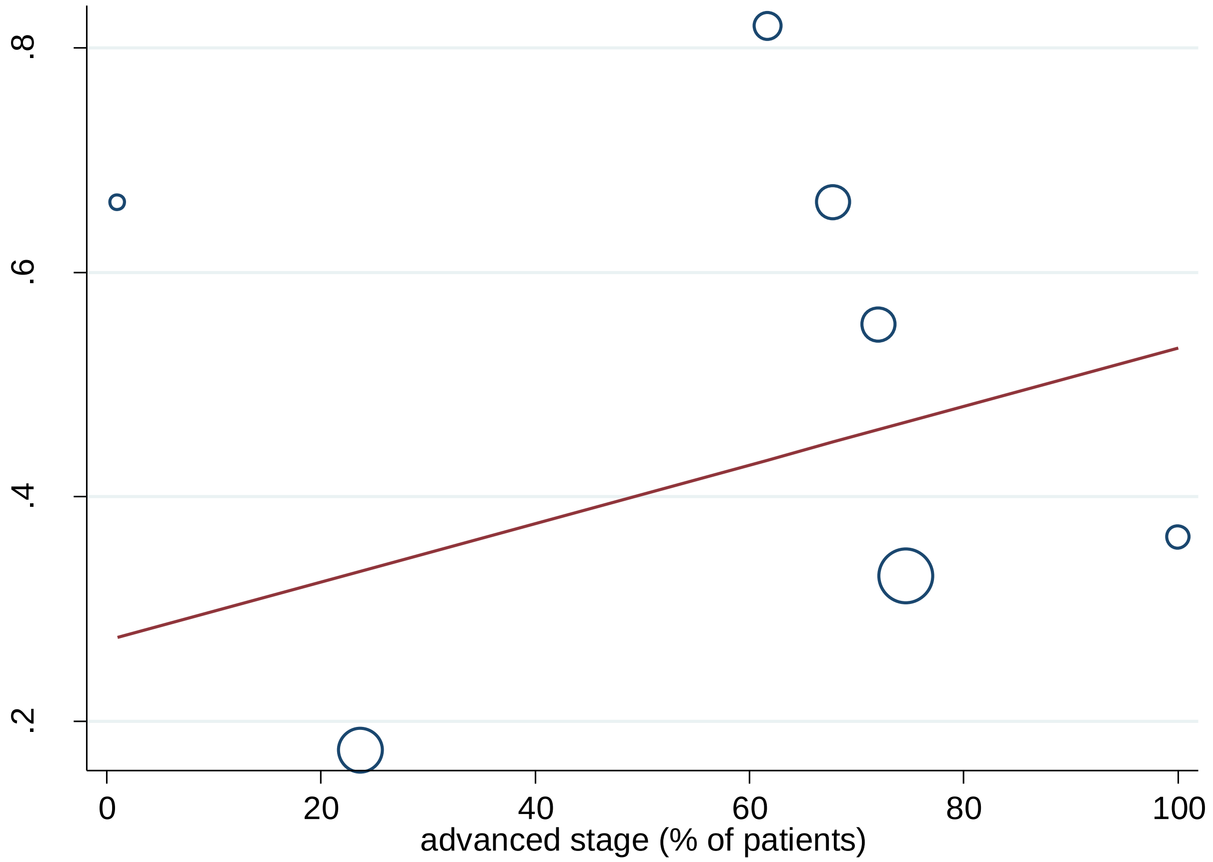

HNSCC, head and neck squamous cell carcinoma; HR, hazard ratio. The red line exhibits the fitted regression line together with blue circles representing the estimates from each individual study, sized according to the precision of each estimate (the inverse of its within-study variance).

4.9 Effect of follow up period on the association between FADD and overall survival

Figure S11. Bubble plot graphically representing the univariable meta-regression analysis of the potential effect of follow up period (expressed In months) on the association between FADD and overall survival among patients with HNSCC.

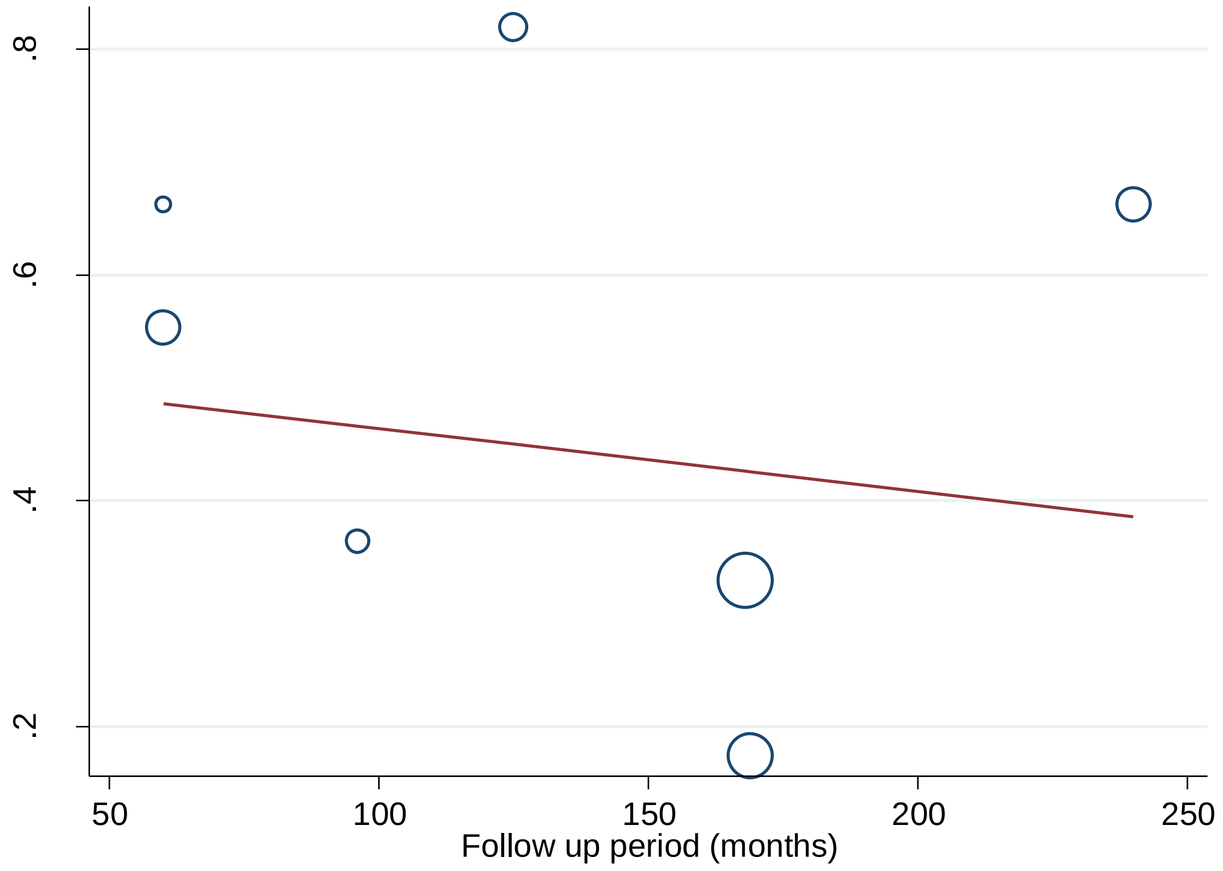

HNSCC, head and neck squamous cell carcinoma; HR, hazard ratio. The red line exhibits the fitted regression line together with blue circles representing the estimates from each individual study, sized according to the precision of each estimate (the inverse of its within-study variance).

5. Meta-analysis on the association between FADD alterations and disease-specific survival

Figure S12. Forest plot graphically representing the stratified analysis of the association between FADD alterations and disease-specific survival in patients with HNSCC.

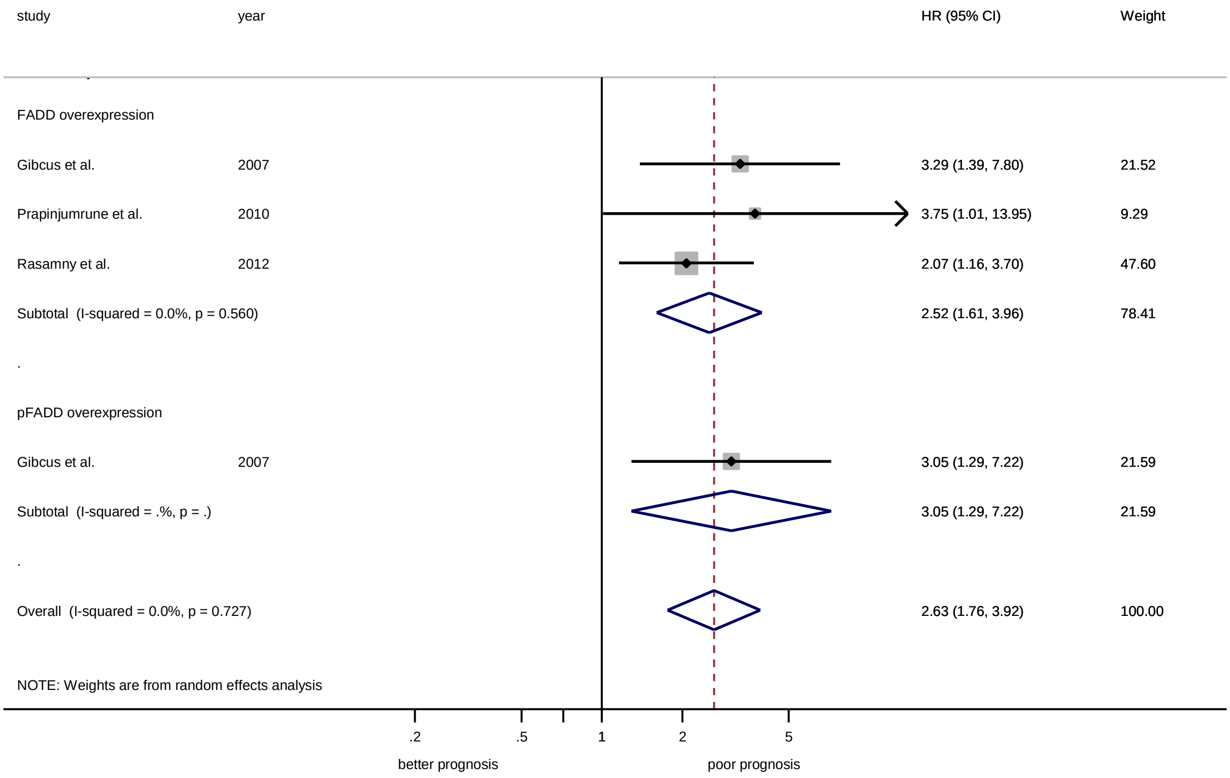

HNSCC, head and neck squamous cell carcinoma; HR, hazard ratio; CI, confidence intervals. Random-effects model, inverse-variance weighting (based on the DerSimonian and Laird method). A HR > 1 suggests that FADD alterations are associated with poor disease-specific survival. Diamonds indicate the pooled HRs with their corresponding 95% CIs.

6. Meta-analysis on the association between FADD alterations and disease-free survival

Figure S13. Forest plot graphically representing the stratified analysis of the association between FADD alterations and disease-free survival in patients with HNSCC.

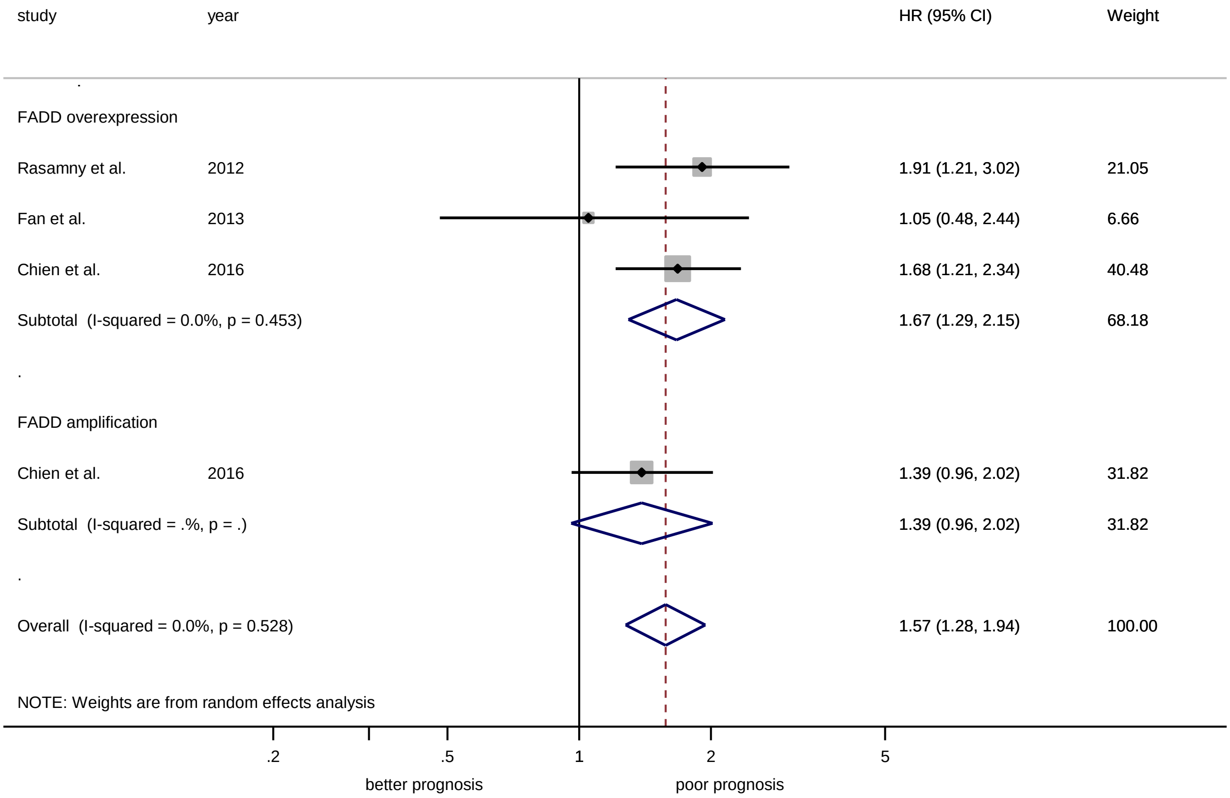

HNSCC, head and neck squamous cell carcinoma; HR, hazard ratio; CI, confidence intervals. Random-effects model, inverse-variance weighting (based on the DerSimonian and Laird method). A HR > 1 suggests that FADD alterations are associated with poor disease-free survival. Diamonds indicate the pooled HRs with their corresponding 95% CIs.

7. Meta-analysis on the association between FADD alterations and T status

Figure S14. Forest plot graphically representing the stratified analysis of the association between FADD alterations and T status (T3/T4 vs. T1/T2) in patients with HNSCC.

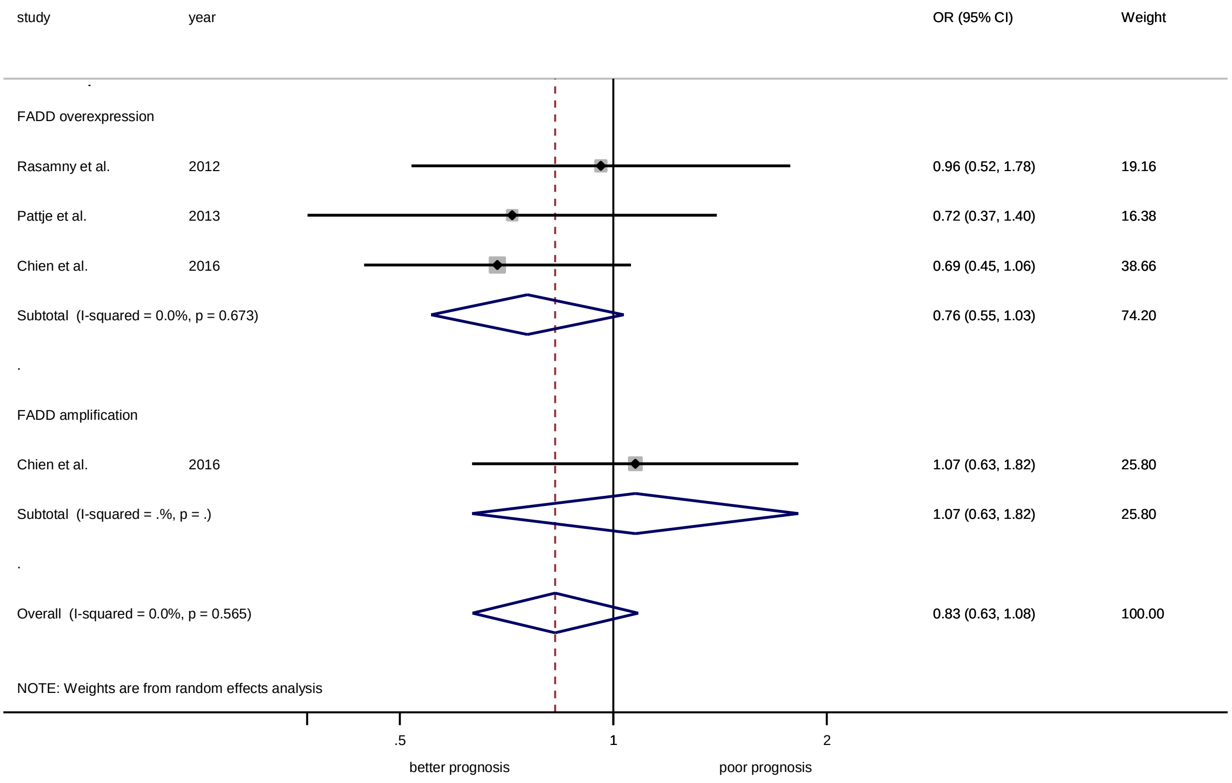

HNSCC, head and neck squamous cell carcinoma; OR, odds ratio; CI, confidence intervals. Random-effects model, inverse-variance weighting (based on the DerSimonian and Laird method). A OR > 1 suggests that FADD alterations are associated with a higher T status. Diamonds indicate the pooled ORs with their corresponding 95% CIs.

8. Meta-analysis on the association between FADD alterations and N status

8.1 Galbraith plot of the association between FADD alterations and N status

Figure S15. Galbraith plot of the association between FADD alterations and N status in HNSCC, constructed to examine the contributions of individual studies to the heterogeneity metrics and identify outliers. It contains additional information, allowing the identification of studies (data not showed in Figure 4 due to graphic purposes).

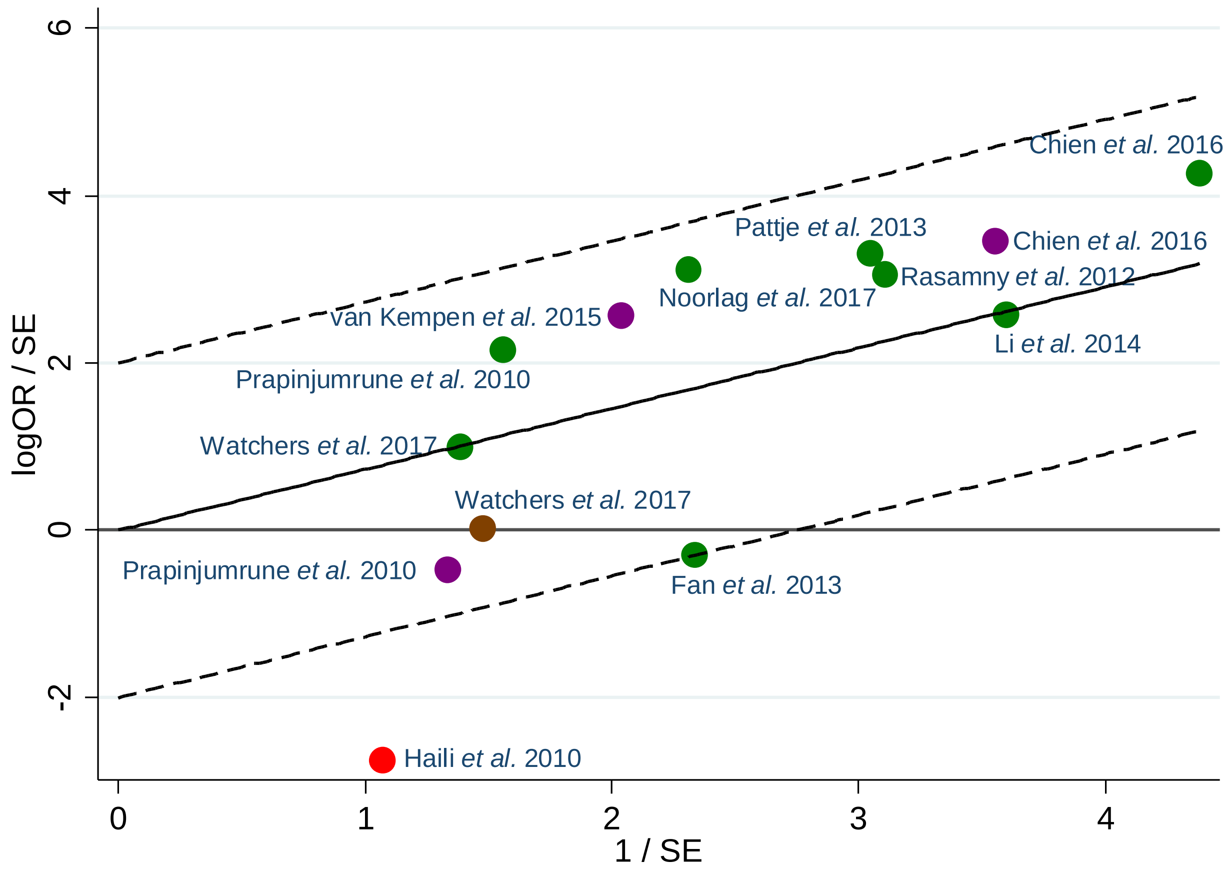

OR, odds ratio; SE, standard error. The vertical axis represents the observed effect sizes standardized by their corresponding standard errors (y=logOR/SE[logOR]) against precision on the horizontal axis (x=1/SE[logOR]). The regression diagonal line is projected from the origin (0,0), and the approximate 95% confidence intervals run between the two intermittent parallel lines at ±2 units above and below the regression line. The studies inside this 95% confidence region were represented as green (FADD overexpression), brown (pFADD overexpression) and purple (FADD amplification) circles. The study below the confidence limits was identified as a significant outlier (Haili et al. 2010, depicted as a red circle), contributing disproportionately to the observed heterogeneity.

8.2 Sensitivity analysis with the omission of the identified outlier.

Figure S16. Forest plot graphically representing the stratified analysis of the association between FADD alterations and N status (positive vs. negative) in patients with HNSCC, with the omission of the outlier (Haili et al. 2010) identified in the previous figure (S14).

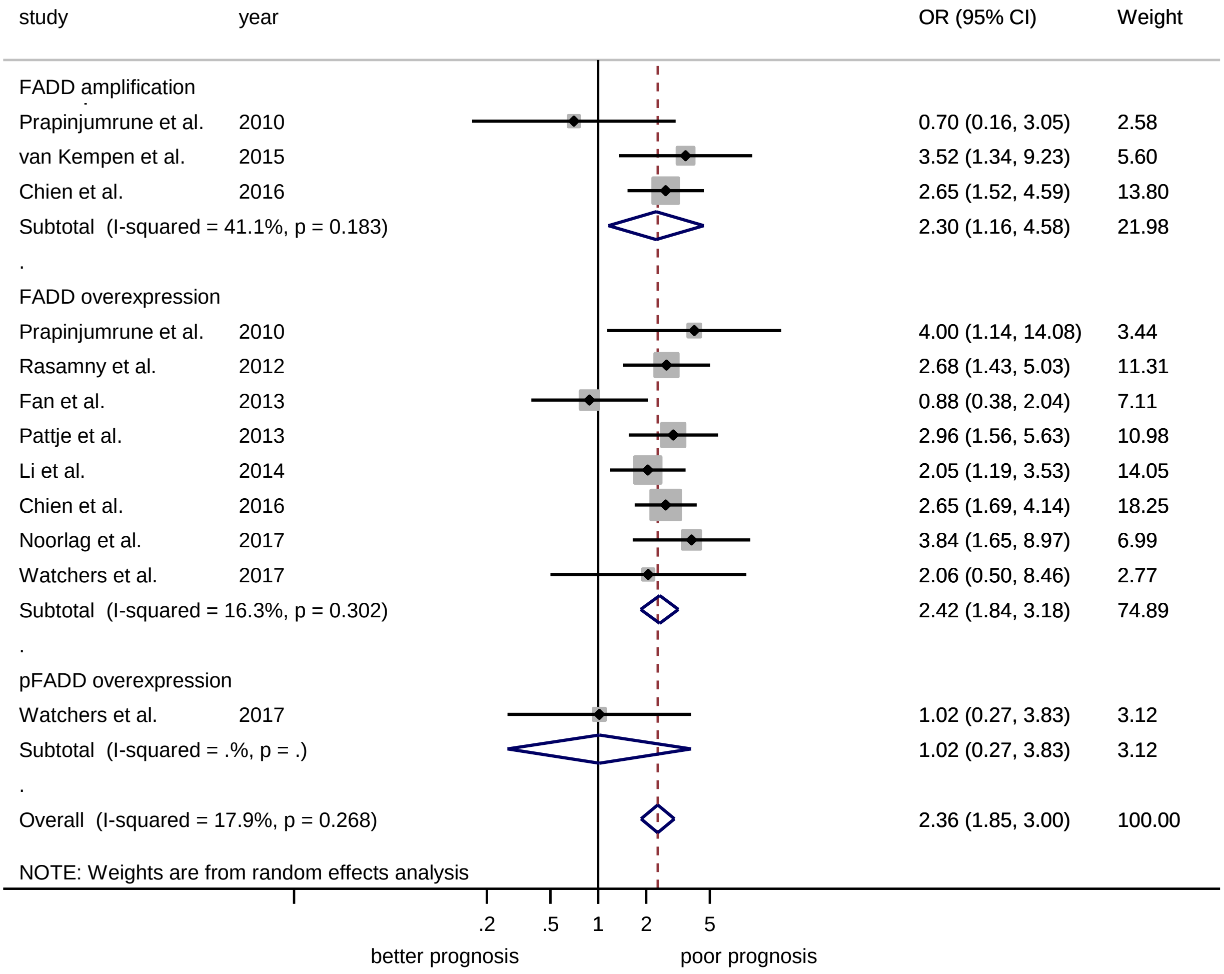

HNSCC, head and neck squamous cell carcinoma; OR, odds ratio; CI, confidence intervals. Random-effects model, inverse-variance weighting (based on the DerSimonian and Laird method). A OR > 1 suggests that FADD alterations are associated with a higher T status. Diamonds indicate the pooled ORs with their corresponding 95% CIs.

9. Meta-analysis on the association between FADD alterations and clinical stage

Figure S17. Forest plot graphically representing the stratified analysis of the association between FADD alterations and clinical stage (III/IV vs. II) in patients with HNSCC.

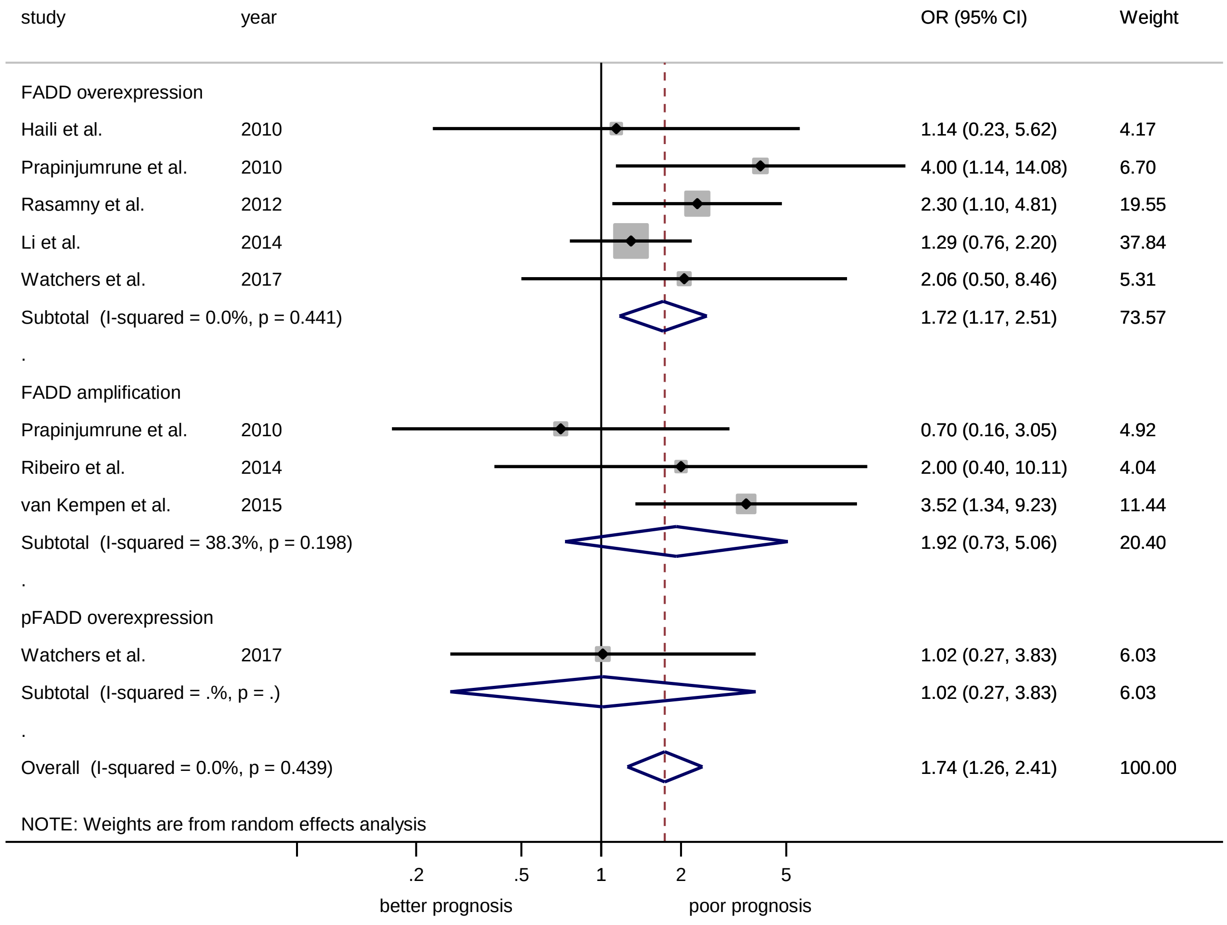

HNSCC, head and neck squamous cell carcinoma; OR, odds ratio; CI, confidence intervals. Random-effects model, inverse-variance weighting (based on the DerSimonian and Laird method). A OR > 1 suggests that FADD alterations are associated with a higher T status. Diamonds indicate the pooled ORs with their corresponding 95% CIs.

10. Analysis of small-study effects

10.1 FADD overexpression and Overall Survival in HNSCC

Figure S18. A funnel plot of estimated logHR against its standard error, graphically representing the analysis of small-study effects on Overall Survival in HNSCC.

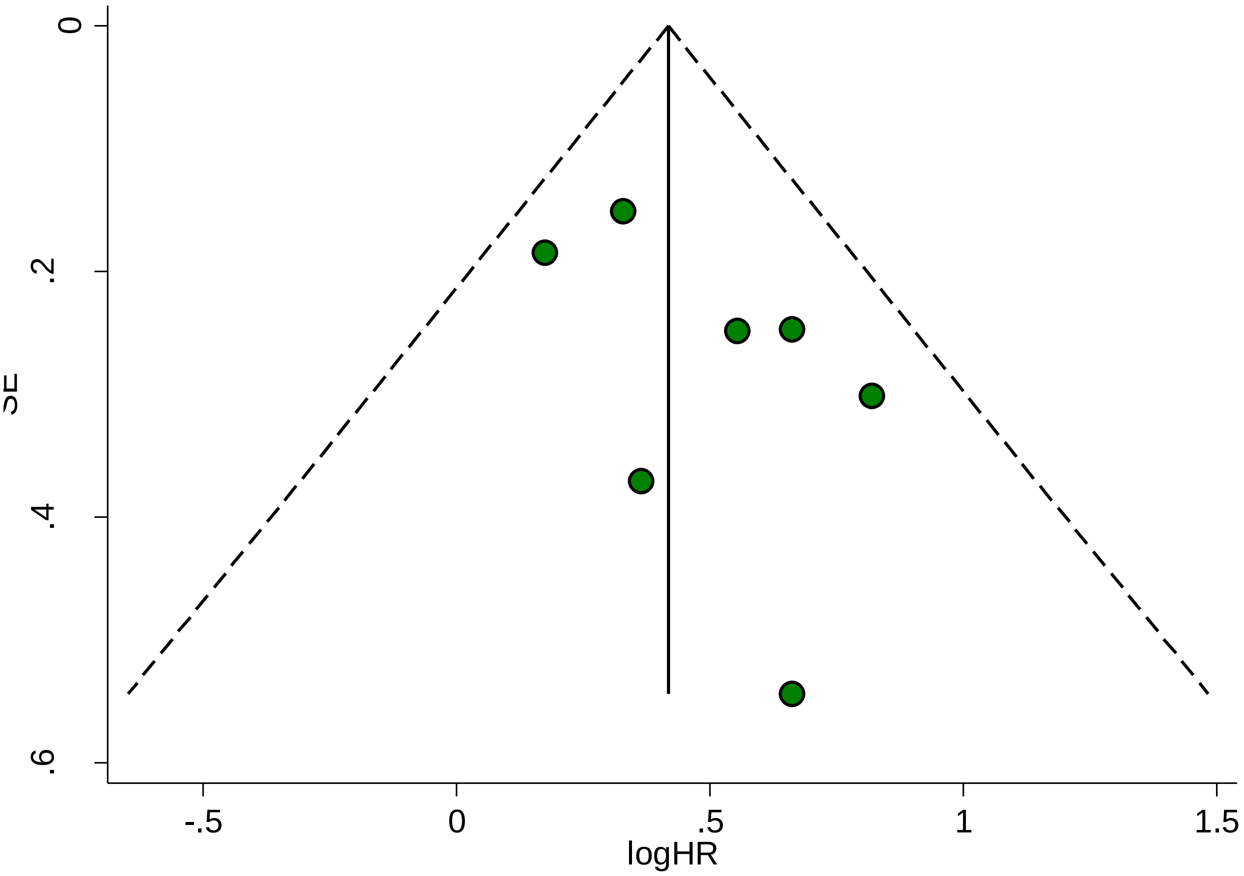

SE, standard error; HR, hazard ratio. The black vertical line corresponds to the pooled estimated prevalence. The two diagonal intermittent lines represent the pseudo-95% confidence interval. The green circles represent the published studies reporting the association between FADD overexpression and Overall Survival.

10.2 pFADD overexpression and Overall Survival in HNSCC

Figure S19. A funnel plot of estimated logHR against its standard error, graphically representing the analysis of small-study effects on Overall Survival in HNSCC.

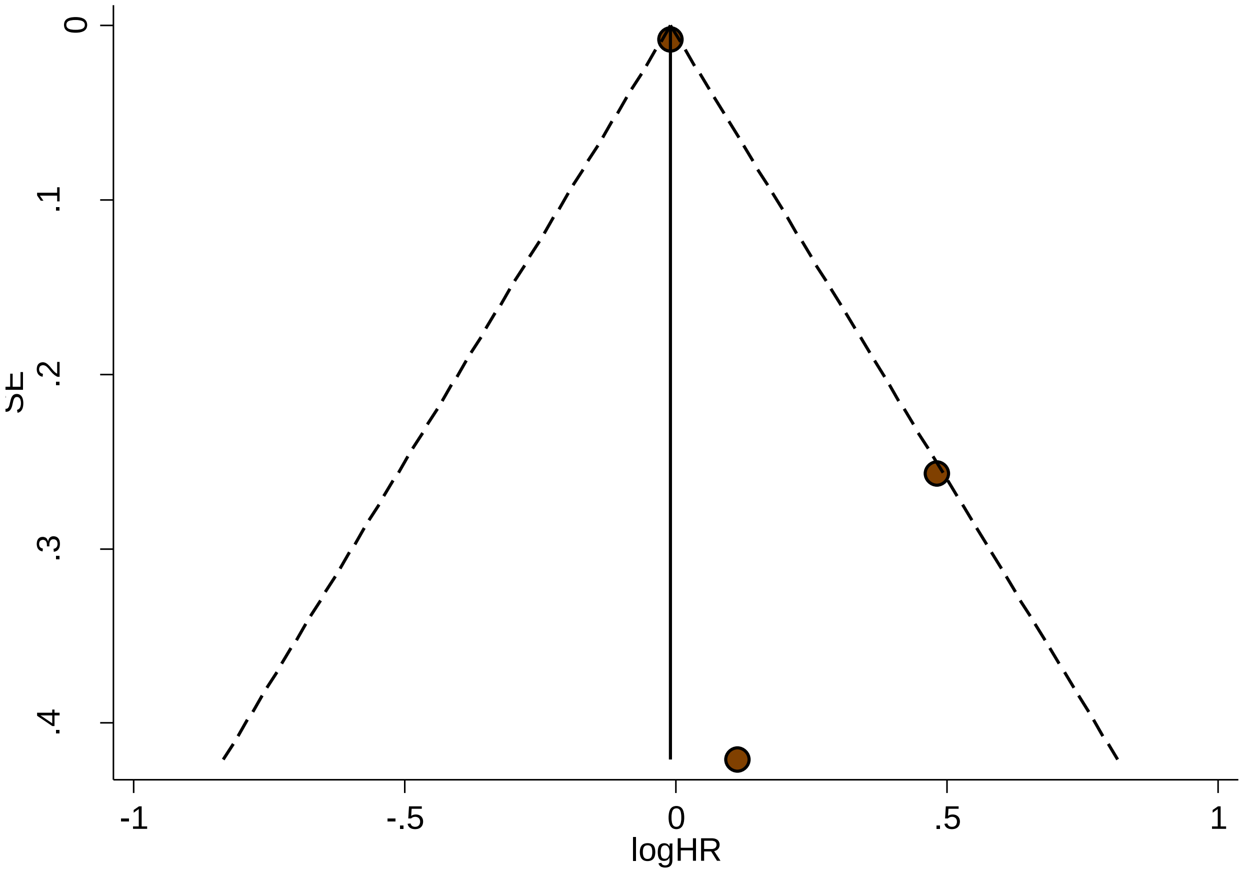

SE, standard error; HR, hazard ratio. The black vertical line corresponds to the pooled estimated prevalence. The two diagonal intermittent lines represent the pseudo-95% confidence interval. The brown circles represent the published studies reporting the association between pFADD overexpression and Overall survival.

10.3 FADD overexpression and Disease-Specific Survival in HNSCC

Figure S20. A funnel plot of estimated logHR against its standard error, graphically representing the analysis of small-study effects on Disease-Specific Survival in HNSCC.

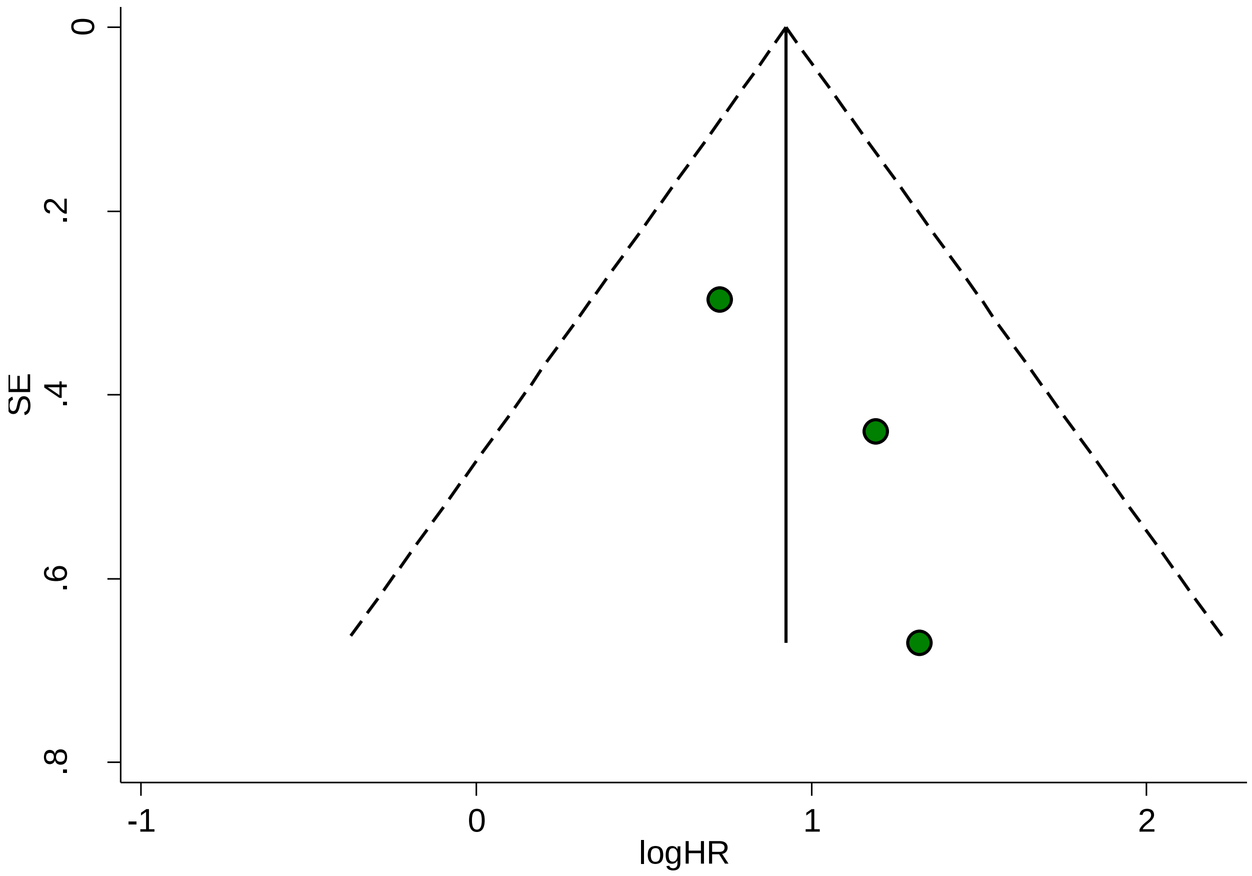

SE, standard error; HR, hazard ratio. The black vertical line corresponds to the pooled estimated prevalence. The two diagonal intermittent lines represent the pseudo-95% confidence interval. The green circles represent the published studies reporting the association between FADD overexpression and Disease-Specific survival.

10.4 FADD overexpression and Disease-Free Survival in HNSCC

Figure S21. A funnel plot of estimated logHR against its standard error, graphically representing the analysis of small-study effects on Disease-Free Survival in HNSCC.

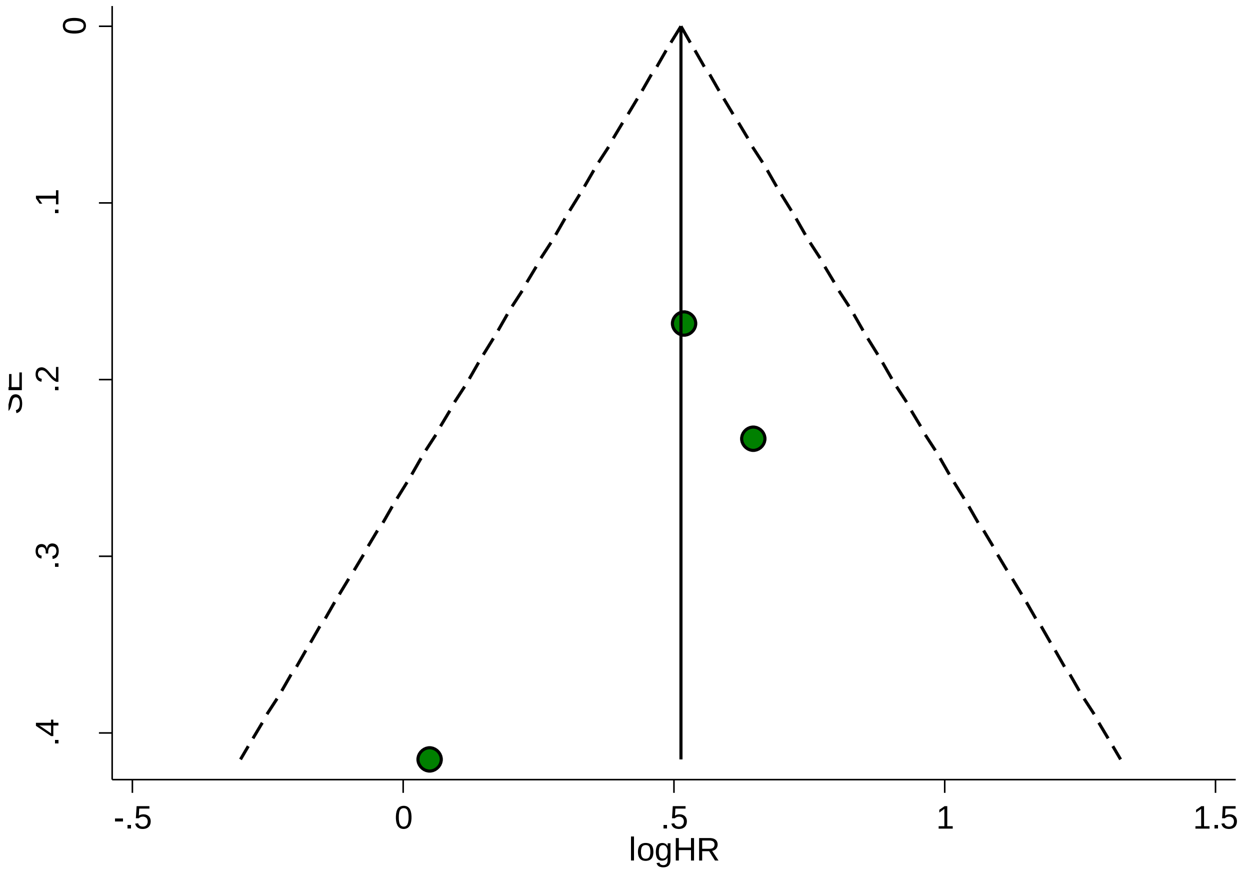

SE, standard error; HR, hazard ratio. The black vertical line corresponds to the pooled estimated prevalence. The two diagonal intermittent lines represent the pseudo-95% confidence interval. The green circles represent the published studies reporting the association between FADD overexpression and Disease-Free survival.

10.5 FADD overexpression and T status in HNSCC

Figure S22. A funnel plot of estimated logOR against its standard error, graphically representing the analysis of small-study effects on T status in HNSCC.

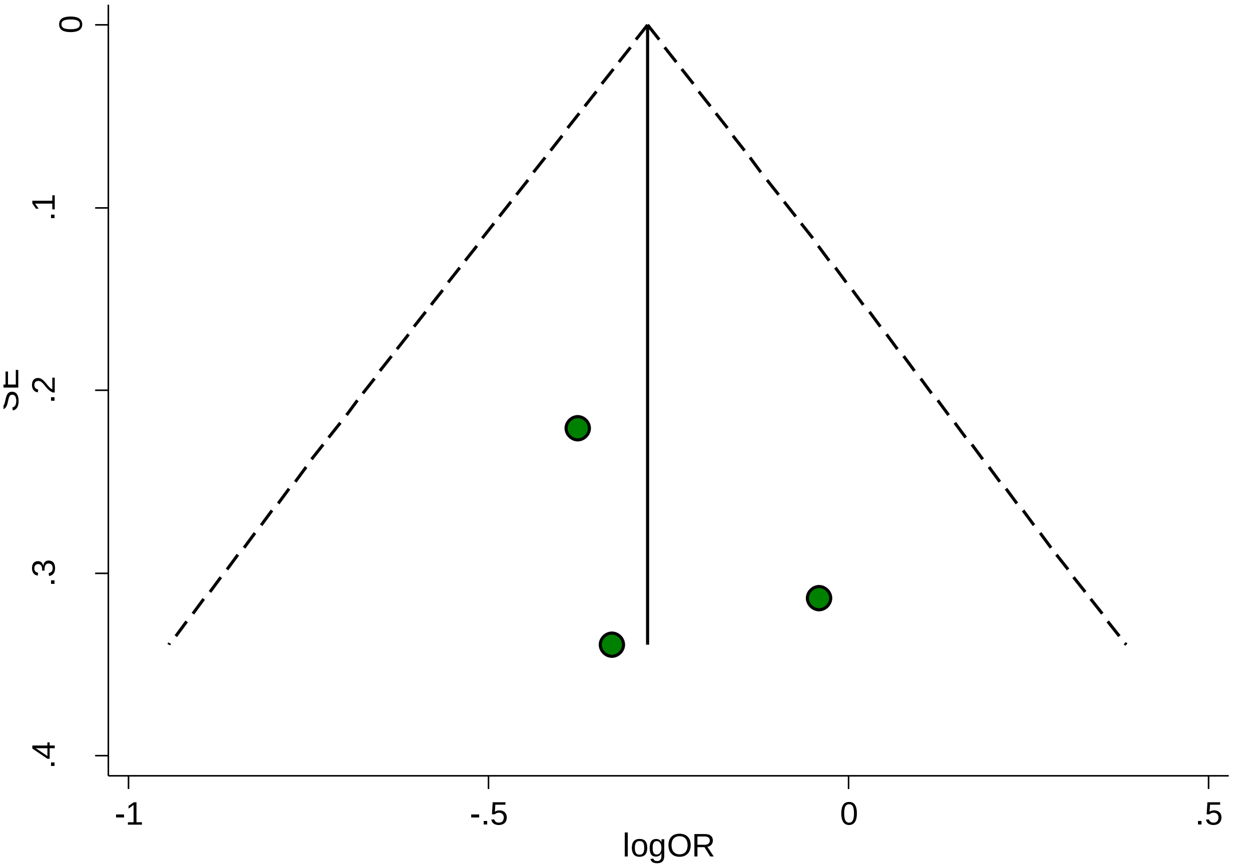

SE, standard error; OR, odds ratio. The black vertical line corresponds to the pooled estimated prevalence. The two diagonal intermittent lines represent the pseudo-95% confidence interval. The green circles represent the published studies reporting the association between FADD overexpression and T status.

10.6 FADD overexpression and N status in HNSCC

Figure S23. A funnel plot of estimated logOR against its standard error, graphically representing the analysis of small-study effects on N status in HNSCC.

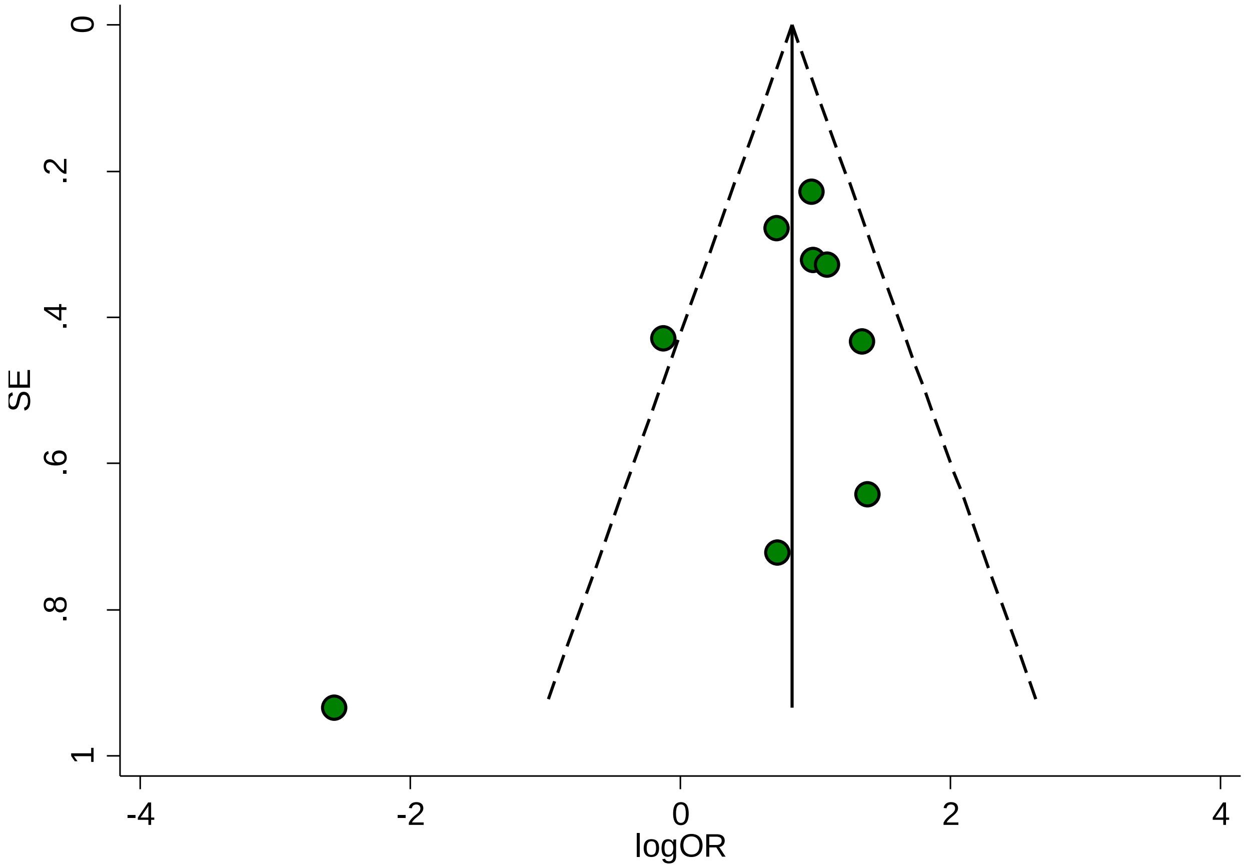

SE, standard error; OR, odds ratio. The black vertical line corresponds to the pooled estimated prevalence. The two diagonal intermittent lines represent the pseudo-95% confidence interval. The green circles represent the published studies reporting the association between FADD overexpression and N status.

10.7 FADD amplification and N status in HNSCC

Figure S24. A funnel plot of estimated logOR against its standard error, graphically representing the analysis of small-study effects on N status in HNSCC.

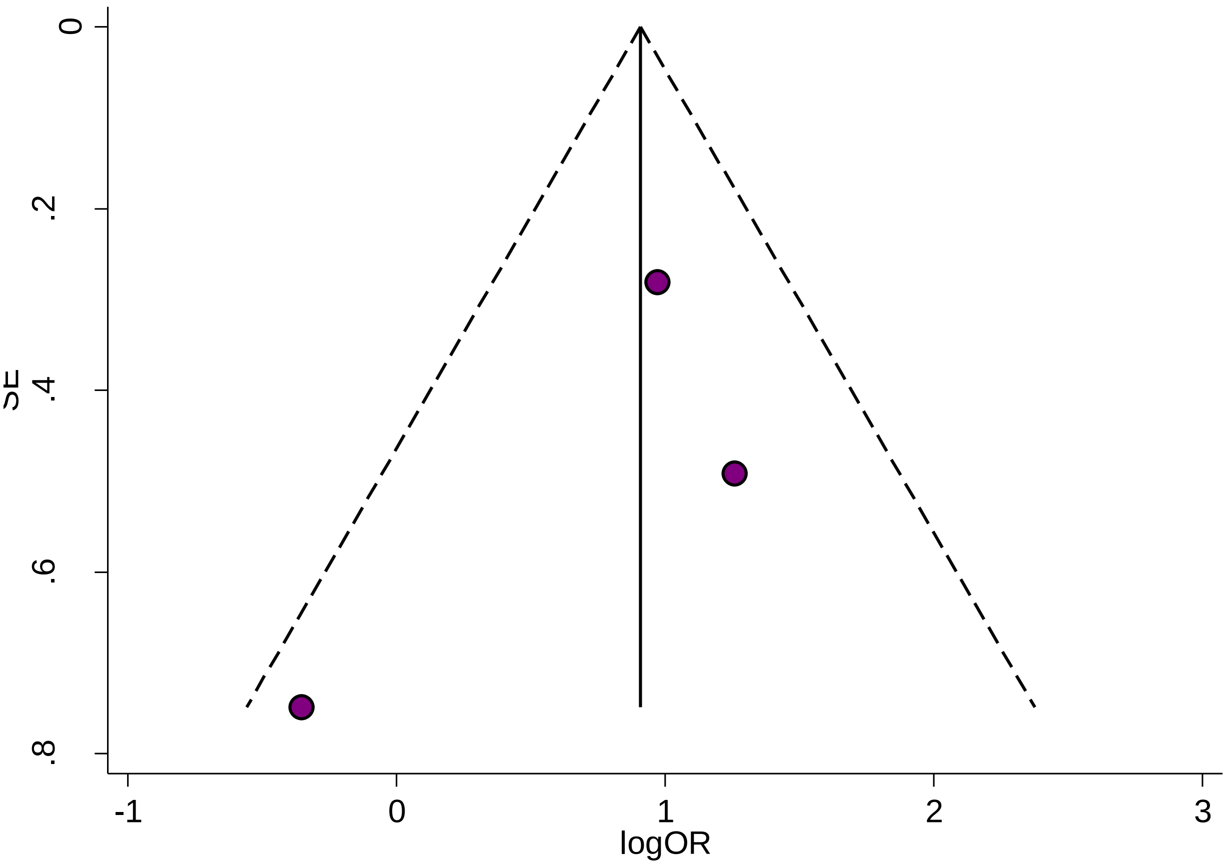

SE, standard error; OR, odds ratio. The black vertical line corresponds to the pooled estimated prevalence. The two diagonal intermittent lines represent the pseudo-95% confidence interval. The purple circles represent the published studies reporting the association between *FADD* amplification and N status.

10.8 FADD overexpression and Clinical Stage in HNSCC

Figure S25. A funnel plot of estimated logOR against its standard error, graphically representing the analysis of small-study effects on Clinical Stage in HNSCC.

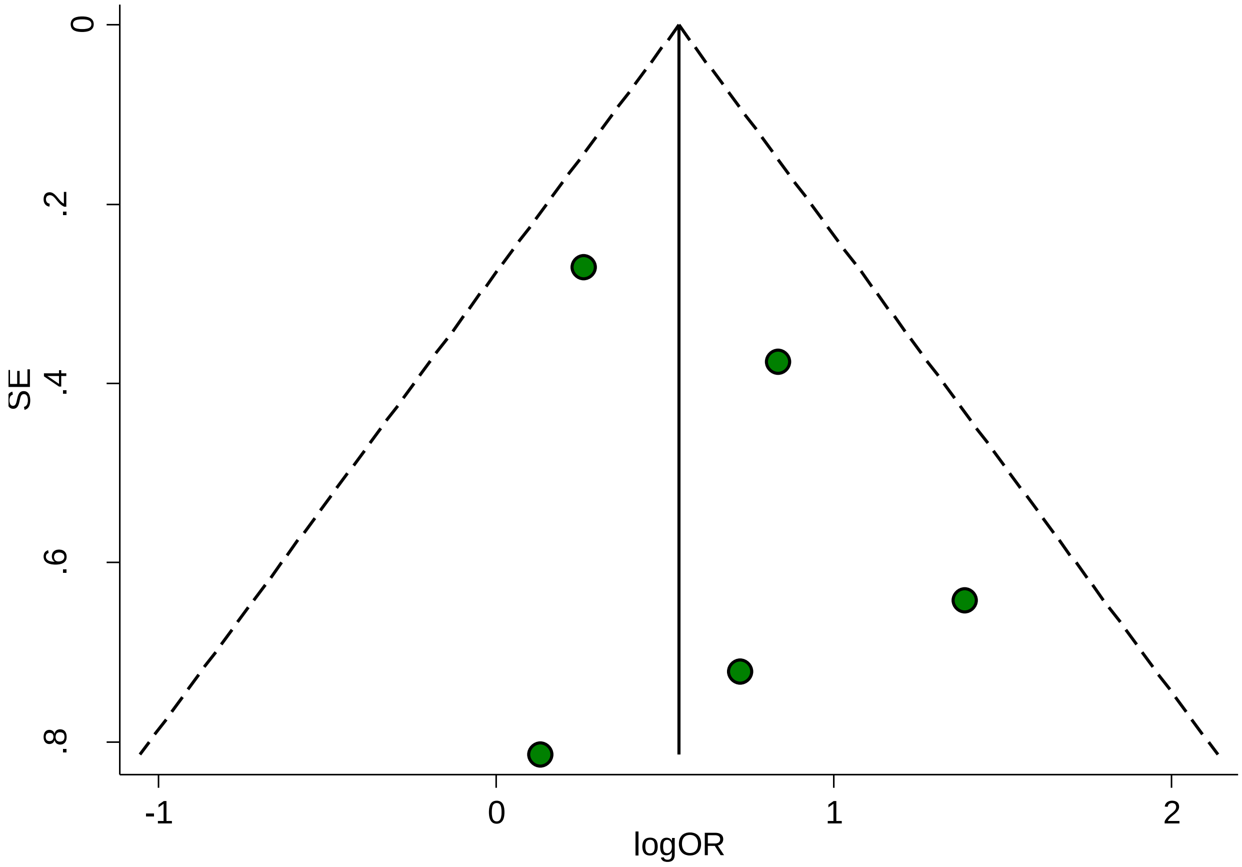

SE, standard error; OR, odds ratio. The black vertical line corresponds to the pooled estimated prevalence. The two diagonal intermittent lines represent the pseudo-95% confidence interval. The green circles represent the published studies reporting the association between FADD overexpression and Clinical Stage.

10.9 FADD amplification and Clinical Stage in HNSCC

Figure S26. A funnel plot of estimated logOR against its standard error, graphically representing the analysis of small-study effects on Clinical Stage in HNSCC.

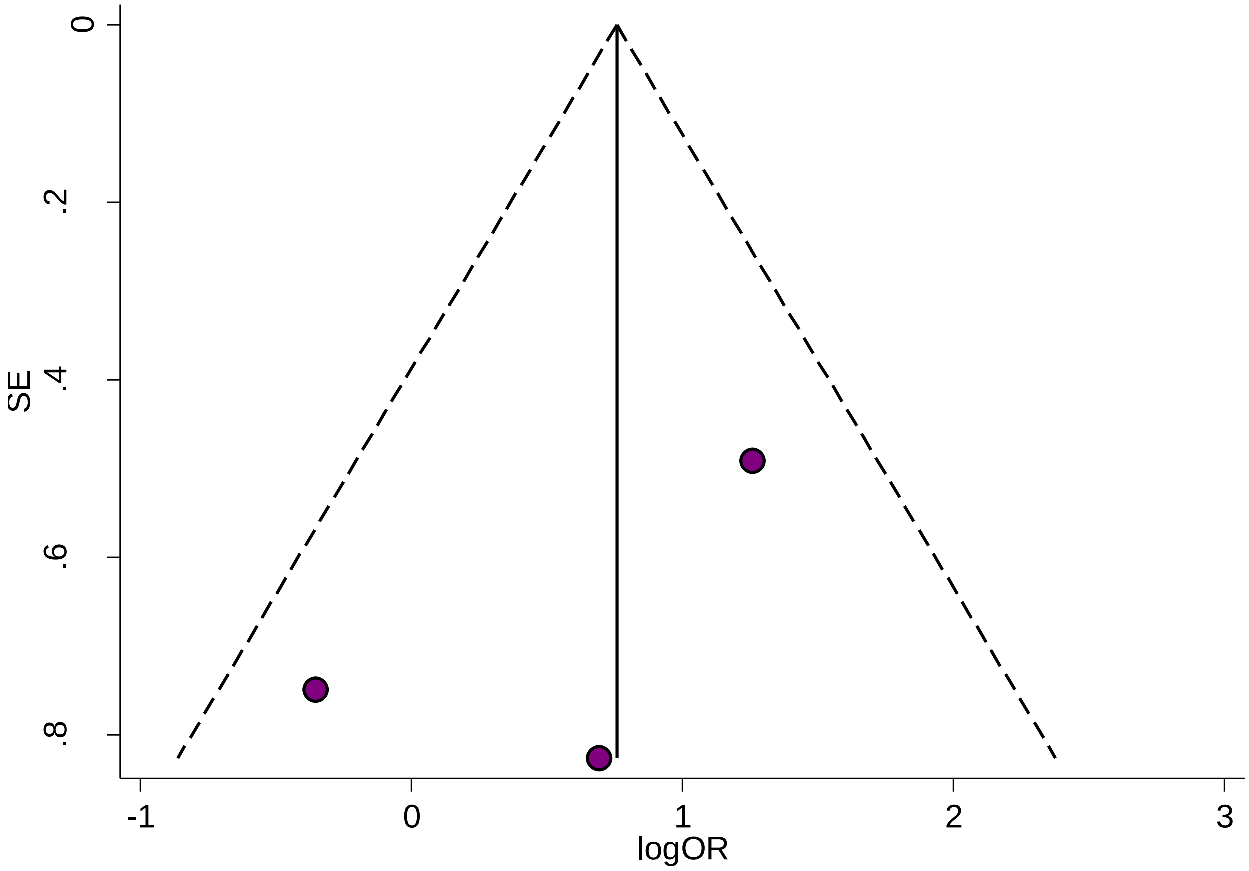

SE, standard error; OR, odds ratio. The black vertical line corresponds to the pooled estimated prevalence. The two diagonal intermittent lines represent the pseudo-95% confidence interval. The purple circles represent the published studies reporting the association between *FADD* amplification and Clinical Stage.

11. Sensitivity analysis (leave-one-out method).

11.1 FADD overexpression and Overall Survival in HNSCC

Table S3. Sensitivity analysis of the studies pooled in the meta-analysis on the association between FADD overexpression and overall survival in HNSCC.

**Study omitted**	**Estimate**	**[95% Conf. Interval]**	
Gibcus et al. (2007)	1.4906355	1.2364639	1.7970554
Schrijvers et al. (2012)	1.513523	1.2639613	1.8123593
Rasamny et al. (2012)	1.4653386	1.2169495	1.764426
Fan et al. (2013)	1.5351375	1.271728	1.8531061
Li et al. (2014)	1.462427	1.2197764	1.7533484
Chien et al. (2016)	1.5915769	1.2847401	1.9716961
Watchers et al. (2017)	1.634128	1.3410536	1.9912508
**Combined**	1.5190571	1.2772069	1.8067038

Figure S27. Interval plot graphically representing the sensitivity analysis from Table S3.

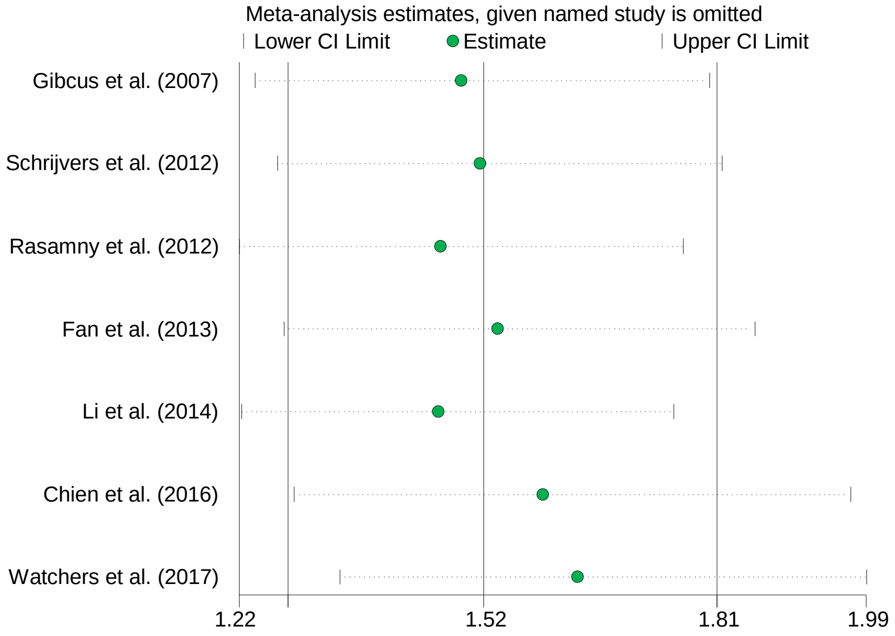

Sensitivity analysis ("leave-one-out" method) of the meta-analysis results, sequentially omitting one study at a time.

11.2 pFADD overexpression and Overall Survival in HNSCC

Table S4. Sensitivity analysis of the studies pooled in the meta-analysis on the association between pFADD overexpression and overall survival in HNSCC.

**Study omitted**	**Estimate**	**[95% Conf. Interval]**	
Gibcus et al. (2007)	.99004078	.97522885	1.0050777
Schrijvers et al. (2012)	1.1845671	.7444275	1.8849374
Watchers et al. (2017)	1.4657264	.95400482	2.2519319
**Combined**	1.1351864	.82373875	1.5643893

Figure S28. Interval plot graphically representing the sensitivity analysis from Table S4.

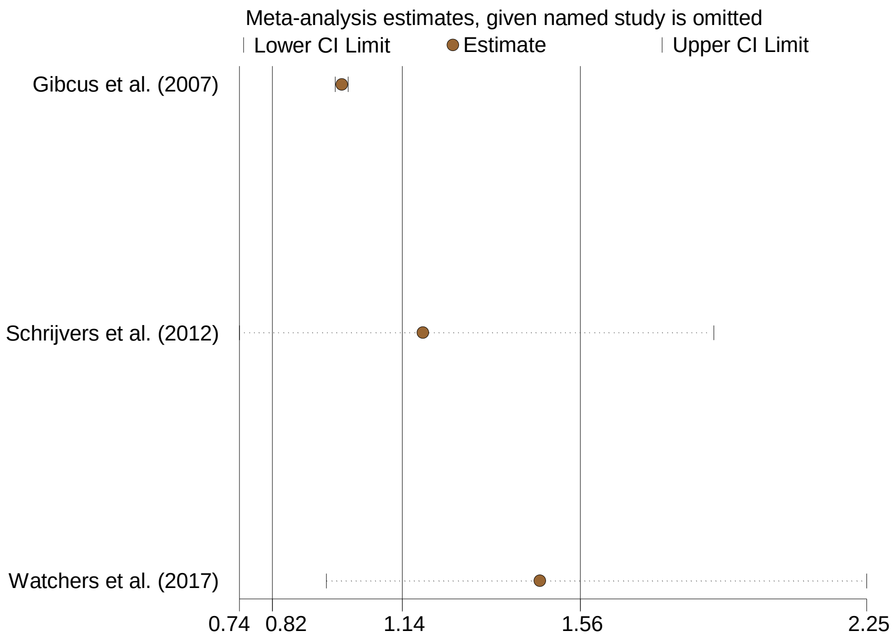

Sensitivity analysis (“leave-one-out” method) of the meta-analysis results, sequentially omitting one study at a time.

11.3 FADD overexpression and Disease-Specific Survival in HNSCC

Table S5. Sensitivity analysis of the studies pooled in the meta-analysis on the association between FADD overexpression and disease-specific survival in HNSCC.

Study omitted	Estimate	[95% Conf. Interval]	
Gibcus et al. (2007)	2.4707458	1.5727993	3.8813503
Gibcus et al. (2007)	2.5222166	1.6052625	3.9629509
Prapinjumrune et al. (2010)	2.53386	1.6646988	3.856822
Rasamny et al. (2012)	3.2637589	1.8779233	5.6722884
**Combined**	**2.6278329**	**1.7612923**	**3.9207041**

Figure S29. Interval plot graphically representing the sensitivity analysis from Table S5.

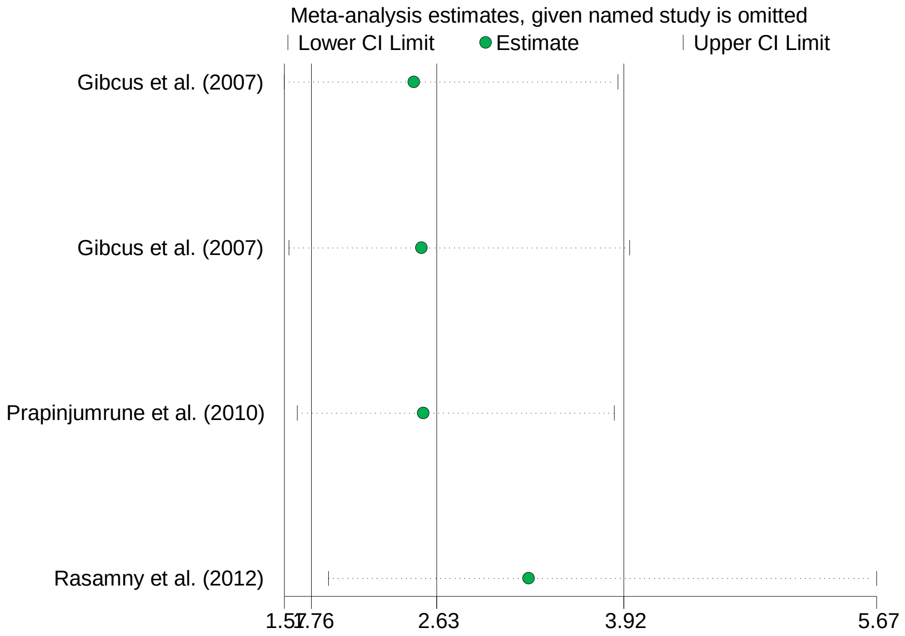

Sensitivity analysis ("leave-one-out" method) of the meta-analysis results, sequentially omitting one study at a time.

11.4 FADD overexpression and Disease-Free Survival in HNSCC

Table S6. Sensitivity analysis of the studies pooled in the meta-analysis on the association between FADD overexpression and disease-free survival in HNSCC.

Study omitted	Estimate	[95% Conf. Interval]	
Rasamny et al. (2012)	1.5476116	1.0908701	2.1955884
Fan et al. (2013)	1.7553831	1.343407	2.2936978
Chien et al. (2016)	1.5624157	.89802778	2.718338
**Combined**	1.669448	1.2948737	2.1523771

Figure S30. Interval plot graphically representing the sensitivity analysis from Table S6.

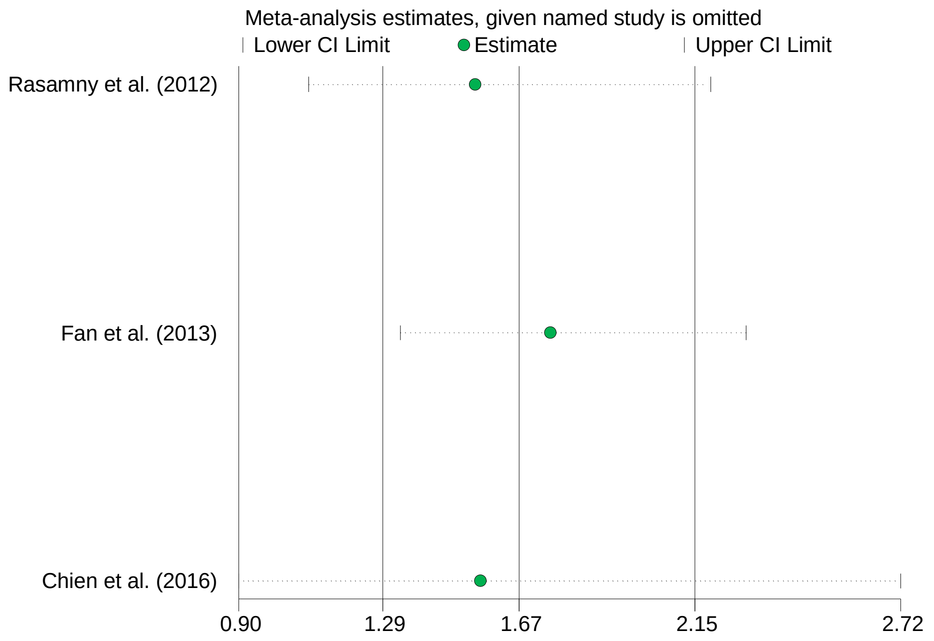

Sensitivity analysis ("leave-one-out" method) of the meta-analysis results, sequentially omitting one study at a time.

11.5 FADD overexpression and T status in HNSCC

Table S7. Sensitivity analysis of the studies pooled in the meta-analysis on the association between FADD overexpression and T status in HNSCC.

**Study omitted**	**Estimate**	**[95% Conf. Interval]**	
Rasamny et al. (2012)	.69625008	.48438099	1.0007912
Pattje et al. (2013)	.76719201	.53848147	1.0930433
Chien et al. (2016)	.84092093	.53537071	1.3208567
**Combined**	.75651804	.553485	1.034029

Figure S31. Interval plot graphically representing the sensitivity analysis from Table S7.

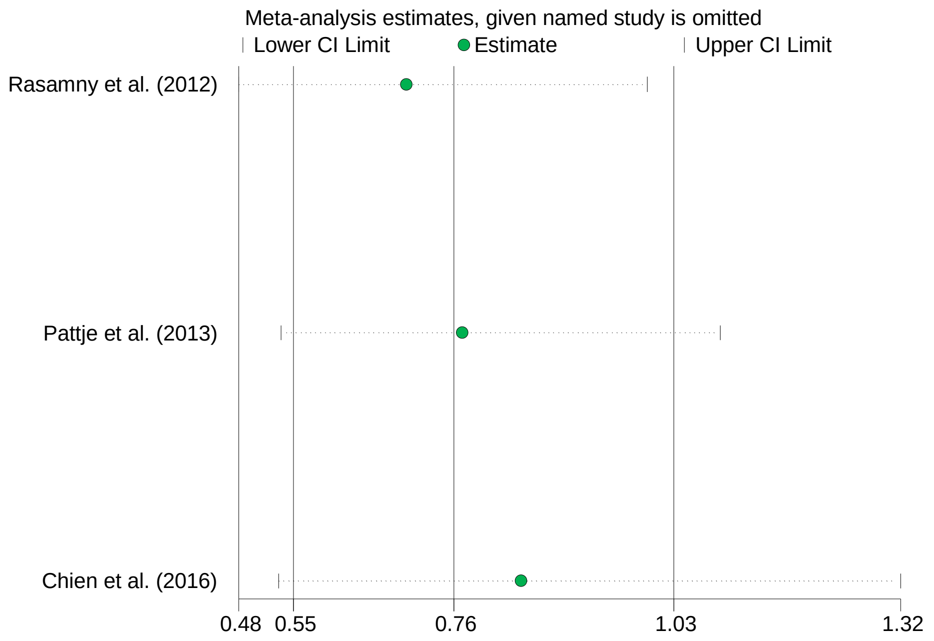

Sensitivity analysis ("leave-one-out" method) of the meta-analysis results, sequentially omitting one study at a time.

11.6 FADD overexpression and N status in HNSCC

Table S8. Sensitivity analysis of the studies pooled in the meta-analysis on the association between FADD overexpression and N status in HNSCC.

Study omitted	Estimate	[95% Conf. Interval]	
Haili et al. (2010)	2.4207382	1.8429391	3.1796894
Prapinjumrune et al. (2010)	1.9542508	1.2323474	3.0990419
Rasamny et al. (2012)	1.9523166	1.1762527	3.2404091
Fan et al. (2013)	2.3477948	1.5364938	3.5874796
Pattje et al. (2013)	1.9280764	1.1692497	3.1793711
Li et al. (2014)	2.0272856	1.2048961	3.4109883
Chien et al. (2016)	1.9254345	1.1291019	3.2834041
Watchers et al. (2017)	2.0574157	1.2923254	3.2754593
Noorlag et al. (2017)	1.9049153	1.1889012	3.0521479
**Combined**	2.0714651	1.342135	3.1971207

Figure S32. Interval plot graphically representing the sensitivity analysis from Table S8.

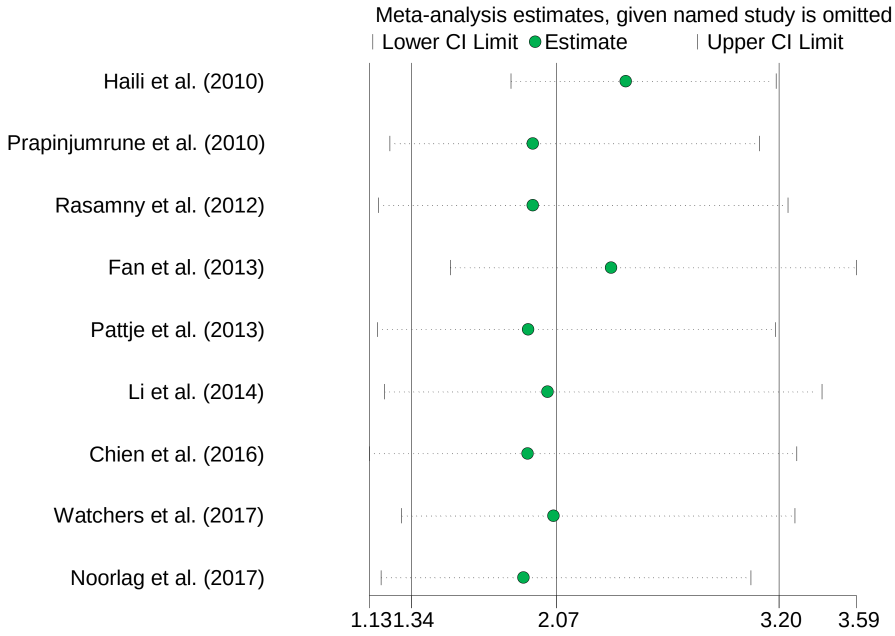

Sensitivity analysis ("leave-one-out" method) of the meta-analysis results, sequentially omitting one study at a time.

11.7 FADD amplification and N status in HNSCC

Table S9. Sensitivity analysis of the studies pooled in the meta-analysis on the association between FADD amplification and N status in HNSCC.

Study omitted	Estimate	[95% Conf.	Interval]
Prapinjumrune et al. (2010)	2.8390503	1.7593453	4.5813675
van Kempen et al. (2015)	1.6357899	.46936208	5.7009473
Chien et al. (2016)	1.7380167	.36254889	8.3318481
**Combined**	2.3030481	1.1580079	4.5803063

Figure S33. Interval plot graphically representing the sensitivity analysis from Table S9.

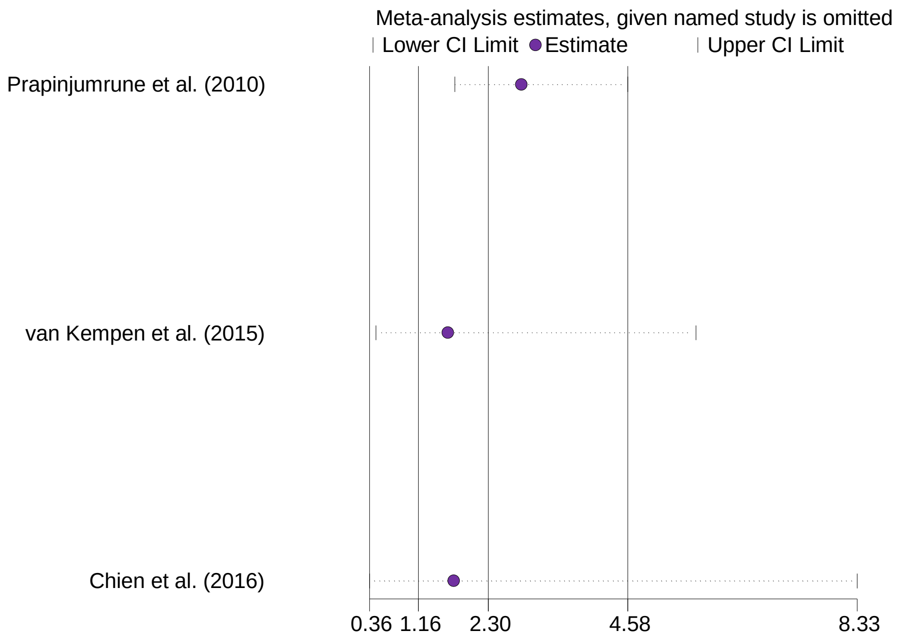

Sensitivity analysis ("leave-one-out" method) of the meta-analysis results, sequentially omitting one study at a time.

11.8 FADD overexpression and Clinical Stage in HNSCC

Table S10. Sensitivity analysis of the studies pooled in the meta-analysis on the association between FADD overexpression and clinical stage in HNSCC.

Study omitted	Estimate	[95% Conf. Interval]	
Haili et al. (2010)	1.8295856	1.1725422	2.8548083
Prapinjumrune et al. (2010)	1.5770947	1.0588968	2.3488858
Rasamny et al. (2012)	1.543282	.99072087	2.4040267
Li et al. (2014)	2.3139443	1.341679	3.9907749
Watchers et al. (2017)	1.7638493	1.101193	2.8252671
**Combined**	1.7165302	1.1741113	2.5095372

Figure S34. Interval plot graphically representing the sensitivity analysis from Table S10.

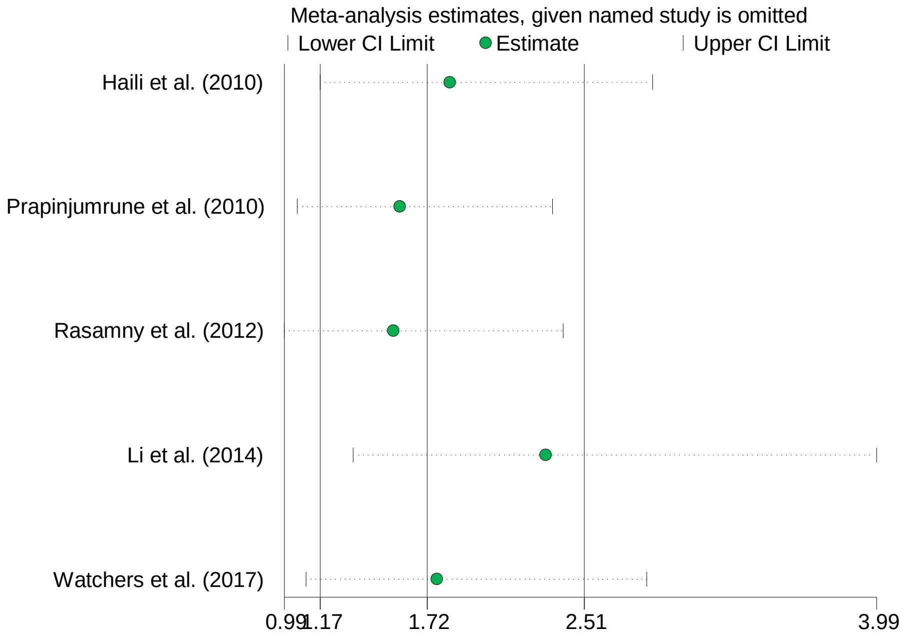

Sensitivity analysis ("leave-one-out" method) of the meta-analysis results, sequentially omitting one study at a time.

11.9 FADD amplification and Clinical Stage in HNSCC

Table S11. Sensitivity analysis of the studies pooled in the meta-analysis on the association between FADD amplification and clinical stage in HNSCC.

Study omitted	Estimate	[95% Conf. Interval]	
Prapinjumrune et al. (2010)	3.0390019	1.3279233	6.9548697
Ribeiro et al. (2014)	1.7380167	.36254889	8.3318481
van Kempen et al. (2015)	1.1265144	.37953743	3.3436351
**Combined**	1.923229	.73110068	5.0592345

Figure S35. Interval plot graphically representing the sensitivity analysis from Table S11.

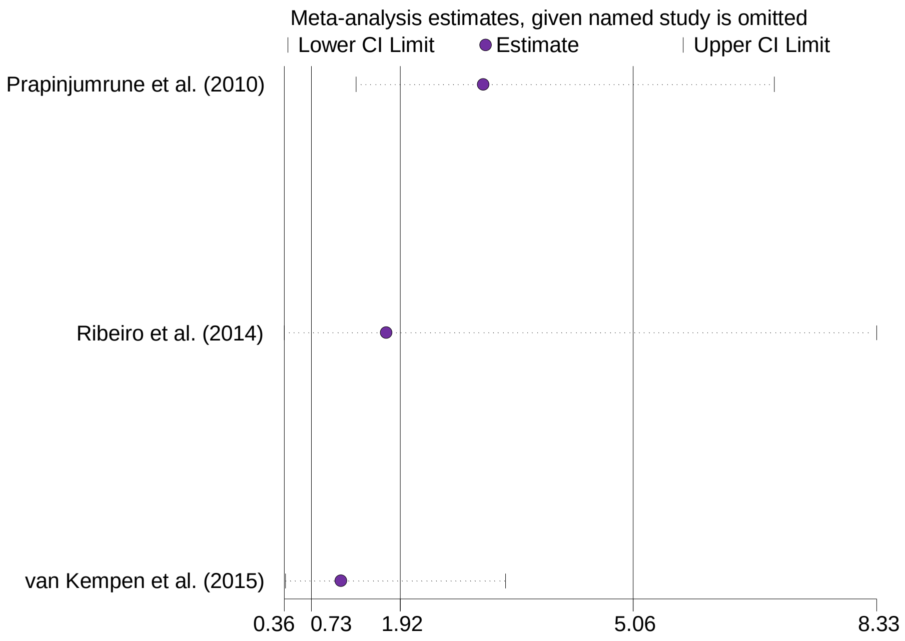

Sensitivity analysis ("leave-one-out" method) of the meta-analysis results, sequentially omitting one study at a time.

12. Sensitivity analysis (by study subsets)

12.1 FADD overexpression and Overall Survival in HNSCC by Quality

Table S12. Sensitivity analysis of the study subsets by overall quality , pooled in the meta-analysis on the association between FADD overexpression and overall survival in HNSCC.

Study subset omitted	Estimate	[95% Conf. Interval]	
Low Risk of Bias (n=6 individual study)	1.44	0.70	2.98
High Risk of Bias (n=1 individual studies)	1.54	1.27	1.85
**Combined**	1.52	1.28	1.81

Figure S36. Interval plot graphically representing the sensitivity analysis from Table S12.

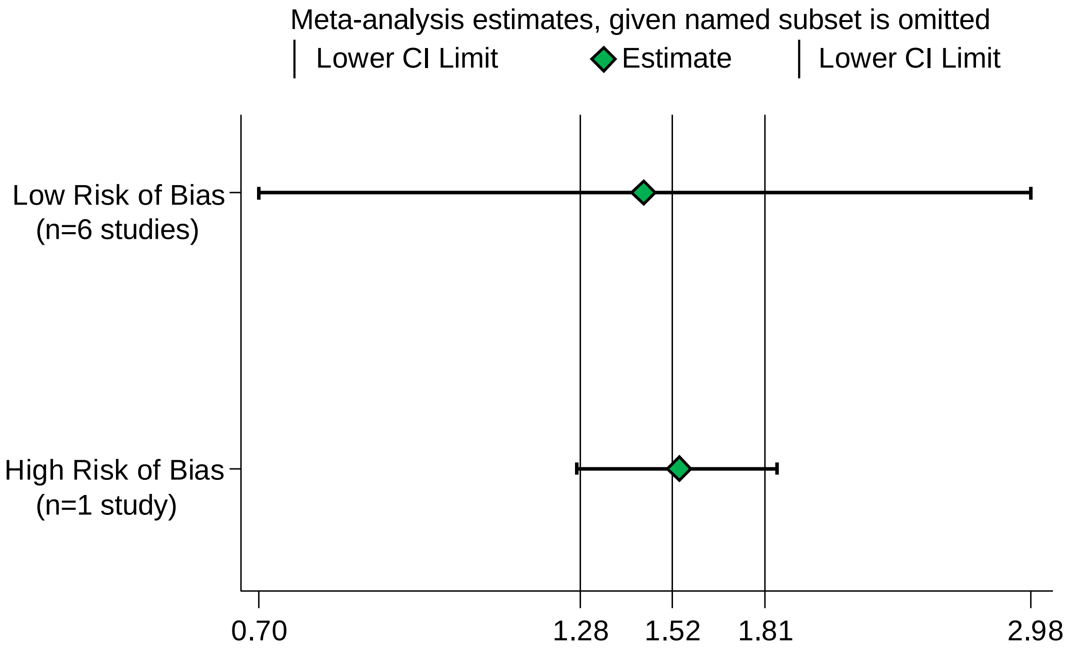

Sensitivity analysis of the meta-analysis results, sequentially omitting one study subset at a time.

12.2 FADD overexpression and Overall Survival in HNSCC by Source of Data

Table S13. Sensitivity analysis of the study subsets by source of data, pooled in the meta-analysis on the association between FADD overexpression and overall survival in HNSCC.

Study subset omitted	Estimate	[95% Conf. Interval]	
Univariable analysis (n=4)	1.92	1.37	2.67
Multivariable model (n=2)	1.40	1.15	1.70
Kaplan-Meier curves (n=1)	1.54	1.27	1.85
**Combined**	1.52	1.28	1.81

Figure S37. Interval plot graphically representing the sensitivity analysis from Table S13.

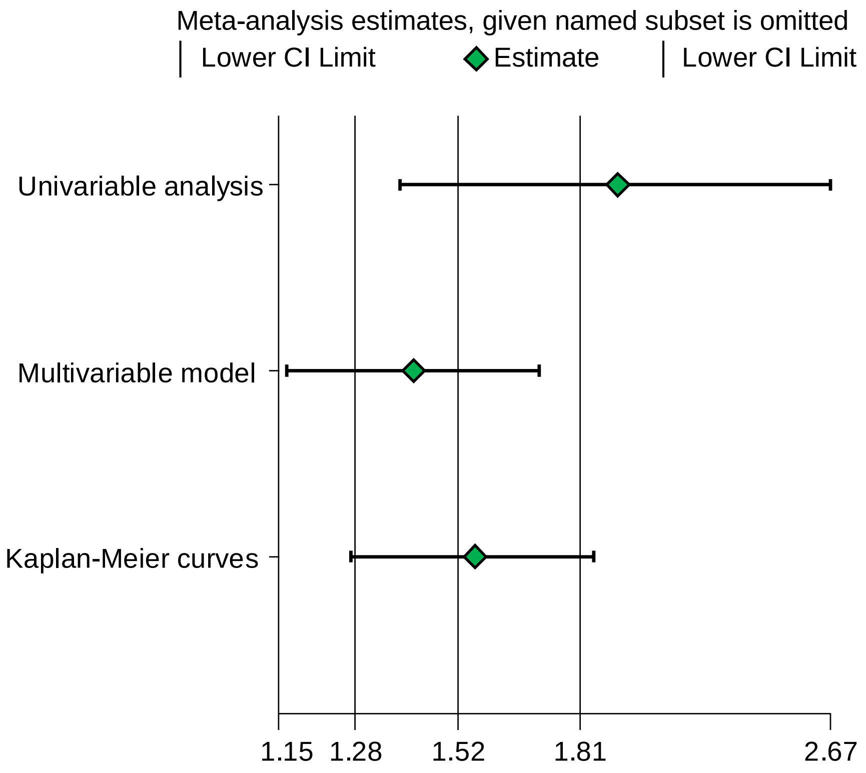

Sensitivity analysis of the meta-analysis results, sequentially omitting one study subset at a time.

12.3 FADD overexpression and N status in HNSCC by Quality

Table S14. Sensitivity analysis of the study subsets by overall quality , pooled in the meta-analysis on the association between FADD overexpression and N status in HNSCC.

**Study subset omitted**	**Estimate**	**[95% Conf. Interval]**	
Low Risk of Bias (n=6)	1.84	0.43	7.78
High Risk of Bias (n=2)	2.57	1.97	3.34
**Combined**	2.42	1.84	3.18

Figure S38. Interval plot graphically representing the sensitivity analysis from Table S14.

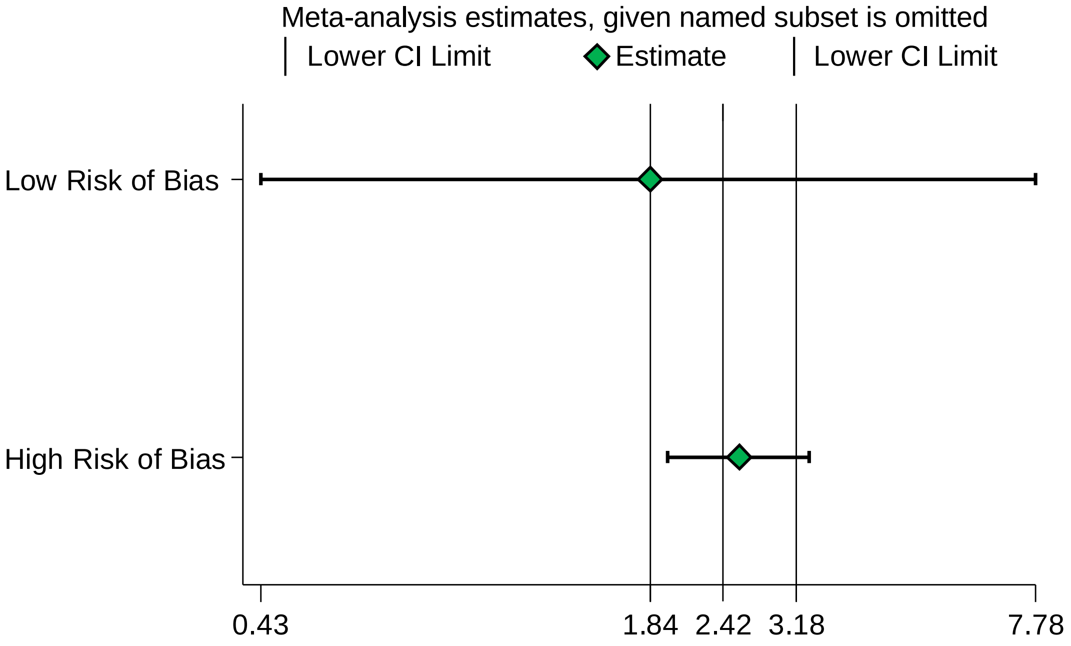

Sensitivity analysis of the meta-analysis results, sequentially omitting one study subset at a time. The outlier previously identified (Haili et al. 2010) was excluded for this analysis.

13. Evaluation of quality of evidence. Table S15. Grading of Recommendations Assessment, Development and Evaluation (GRADE) system

Outcome	Alteration	No of studies	No of patients	Risk of bias	Inconsistency	Indirectness	Imprecision	Publication bias	Other considerations	Overall quality of evidence
Overall survival	*FADD* amplification	1	339	Not serious	Not serious	Not serious	Very serious	Not applied	None	⊕○○○ VERY LOW
Overall survival	pFADD overexpression	3	285	Not serious	Not serious	Not serious	Serious	Undetected	None	⊕○○○ VERY LOW
Overall survival	FADD overexpression	7	1,196	Not serious	Not serious	Not serious	Not serious	Undetected	None	⊕⊕○○ LOW
Disease-specific survival	pFADD overexpression	1	133	Serious	Not serious	Not serious	Very serious	Not applied	Large magnitude of effect	⊕○○○ VERY LOW
Disease-specific survival	FADD overexpression	3	422	Not serious	Not serious	Not serious	Serious	Undetected	Large magnitude of effect	⊕⊕○○ LOW
Disease-free survival	*FADD* amplification	1	339	Not serious	Not serious	Not serious	Very serious	Not applied	None	⊕○○○ VERY LOW
Disease-free survival	FADD overexpression	3	658	Not serious	Not serious	Not serious	Serious	Undetected	None	⊕○○○ VERY LOW
Local recurrence	pFADD overexpression	2	152	Not serious	Serious	Not serious	Very serious	Not applied	None	⊕○○○ VERY LOW
Local recurrence	FADD overexpression	2	150	Not serious	Serious	Not serious	Very serious	Not applied	None	⊕○○○ VERY LOW
T status	*FADD* amplification	1	339	Not serious	Not serious	Not serious	Very serious	Not applied	None	⊕○○○ VERY LOW
T status	FADD overexpression	3	727	Not serious	Not serious	Not serious	Serious	Undetected	None	⊕○○○ VERY LOW
N status	*FADD* amplification	3	533	Not serious	Not serious	Not serious	Serious	Undetected	Large magnitude of effect	⊕⊕○○ LOW
N status	pFADD overexpression	1	59	Not serious	Not serious	Not serious	Very serious	Not applied	None	⊕○○○ VERY LOW
N status	FADD overexpression	9	1,483	Not serious	Not serious	Not serious	Not serious	Undetected	Large magnitude of effect	⊕⊕⊕○ MODERATE
Clinical stage	*FADD* amplification	3	224	Not serious	Not serious	Not serious	Very serious	Undetected	None	⊕○○○ VERY LOW
Clinical stage	pFADD overexpression	1	59	Not serious	Not serious	Not serious	Very serious	Not applied	None	⊕○○○ VERY LOW
Clinical stage	FADD overexpression	5	616	Not serious	Not serious	Not serious	Not serious	Undetected	None	⊕⊕○○ LOW
Histological grade	*FADD* amplification	2	369	Not serious	Very serious	Not serious	Very serious	Not applied	Large magnitude of effect	⊕○○○ VERY LOW
Histological grade	FADD overexpression	3	439	Not serious	Not serious	Not serious	Serious	Not applied	None	⊕○○○ VERY LOW
Bone invasion	*FADD* amplification	1	339	Not serious	Not serious	Not serious	Very serious	Not applied	None	⊕○○○ VERY LOW
Bone invasion	FADD overexpression	1	339	Not serious	Not serious	Not serious	Very serious	Not applied	None	⊕○○○ VERY LOW
Skin invasion	*FADD* amplification	1	339	Not serious	Not serious	Not serious	Very serious	Not applied	None	⊕○○○ VERY LOW
Skin invasion	FADD overexpression	1	339	Not serious	Not serious	Not serious	Very serious	Not applied	None	⊕○○○ VERY LOW
Lymphatic invasion	*FADD* amplification	1	339	Not serious	Not serious	Not serious	Very serious	Not applied	None	⊕○○○ VERY LOW
Lymphatic invasion	FADD overexpression	1	339	Not serious	Not serious	Not serious	Very serious	Not applied	None	⊕○○○ VERY LOW
Vascular invasion	*FADD* amplification	1	339	Not serious	Not serious	Not serious	Very serious	Not applied	None	⊕○○○ VERY LOW
Vascular invasion	FADD overexpression	1	339	Not serious	Not serious	Not serious	Very serious	Not applied	None	⊕○○○ VERY LOW
Perineural invasion	*FADD* amplification	1	339	Not serious	Not serious	Not serious	Very serious	Not applied	None	⊕○○○ VERY LOW
Perineural invasion	FADD overexpression	1	339	Not serious	Not serious	Not serious	Very serious	Not applied	None	⊕○○○ VERY LOW
Margins	FADD overexpression	1	177	Not serious	Not serious	Not serious	Very serious	Not applied	None	⊕○○○ VERY LOW
Tumor thickness	*FADD* amplification	1	339	Not serious	Not serious	Not serious	Very serious	Not applied	None	⊕○○○ VERY LOW
Tumor thickness	FADD overexpression	1	339	Not serious	Not serious	Not serious	Very serious	Not applied	None	⊕○○○ VERY LOW
Extracapsular spread	*FADD* amplification	1	157	Not serious	Not serious	Not serious	Very serious	Not applied	None	⊕○○○ VERY LOW
Extracapsular spread	FADD overexpression	2	264	Not serious	Not serious	Not serious	Very serious	Not applied	None	⊕○○○ VERY LOW

Explanations:

Scoring system: As recommended for observational studies (i.e, all the studies included in this systematic review), an initial baseline overall quality of "LOW QUALITY" of evidence was assigned to each outcome. Then, this rating was "downgraded" based on the following domains: risk of bias, inconsistency, indirectness, imprecision and publication bias; or "upgraded" according to magnitude of effect size. The quality of evidence was classified in one of four levels: very low, low, moderate or high. A "serious" score downgrades one level of evidence, "very serious" two levels. A "large effect size" upgrades one level of evidence.

Risk of bias: Quality was assessed using QUIPS tool. If the overall results from quantitative evaluation were seriously influenced by studies with a higher risk of bias, a "serious" rating was assigned.

Inconsistency: Heterogeneity was assessed through Q test and I^2 statistic, visual inspection analyses of forest and funnel plots, a Galbraith plot, and with subgroups and meta-regression analyses. If the sources of heterogeneity were identified or true potentially subpopulations were found, the outcomes were scored with a "not serious" rating.

Indirectness: according to our judgment and strict eligibility criteria applied, all outcomes were considered as sources of direct evidence (i.e., a research that directly compares the exposures which we are interested in, target subpopulations and outcomes of interest).

Imprecision: Wide confidence intervals, number of studies, small sample size and low event rates were considered for downgrading.

Publication bias: The variables that entered in meta-analysis (if ≥3 studies) were assessed by funnel plots and tests. Note that according to Sterne et al. 2011 and GRADE recommendations, these methods lack statistical power when the number of primary studies is fewer than ten.

Other considerations: An odds ratio>2 was considered as a "large effect size".

GRADE certainty ratings. Very low: The true effect is probably markedly different from the estimated effect; Low: The true effect might be markedly different from the estimated effect; Moderate: The authors believe that the true effect is probably close to the estimated effect; High: The authors have a lot of confidence that the true effect is similar to the estimated effect.

14. Validation of methodological quality

14.1 List S2. AMSTAR2 checklist

AMSTAR 2: a critical appraisal tool for systematic reviews that include randomised or non-randomised studies of healthcare interventions, or both

1. Did the research questions and inclusion criteria for the review include the components of PICO?

For Yes:
- ☑ Population
- ☑ Intervention
- ☑ Comparator group
- ☑ Outcome

Optional (recommended)
- ☑ Timeframe for follow-up

- ☑ Yes
- ☐ No

2. Did the report of the review contain an explicit statement that the review methods were established prior to the conduct of the review and did the report justify any significant deviations from the protocol?

For Partial Yes:
The authors state that they had a written protocol or guide that included ALL the following:
- ☑ review question(s)
- ☑ a search strategy
- ☑ inclusion/exclusion criteria
- ☑ a risk of bias assessment

For Yes:
As for partial yes, plus the protocol should be registered and should also have specified:
- ☑ a meta-analysis/synthesis plan, if appropriate, *and*
- ☑ a plan for investigating causes of heterogeneity
- ☑ justification for any deviations from the protocol

- ☑ Yes
- ☐ Partial Yes
- ☐ No

3. Did the review authors explain their selection of the study designs for inclusion in the review?

For Yes, the review should satisfy ONE of the following:
- ☐ *Explanation for* including only RCTs
- ☑ OR *Explanation for* including only NRSI
- ☐ OR *Explanation for* including both RCTs and NRSI

- ☑ Yes
- ☐ No

4. Did the review authors use a comprehensive literature search strategy?

For Partial Yes (all the following):
- ☑ searched at least 2 databases (relevant to research question)
- ☑ provided key word and/or search strategy
- ☑ justified publication restrictions (e.g. language)

For Yes, should also have (all the following):
- ☑ searched the reference lists / bibliographies of included studies
- ☐ searched trial/study registries — Not relevant for observational studies
- ☑ included/consulted content experts in the field
- ☐ where relevant, searched for grey literature — Considered not relevant and very controversial.
- ☑ conducted search within 24 months of completion of the review

- ☑ Yes
- ☐ Partial Yes
- ☐ No

5. Did the review authors perform study selection in duplicate?

For Yes, either ONE of the following:
- ☑ at least two reviewers independently agreed on selection of eligible studies and achieved consensus on which studies to include
- ☐ OR two reviewers selected a sample of eligible studies and achieved good agreement (at least 80 percent), with the remainder selected by one reviewer.

- ☑ Yes
- ☐ No

AMSTAR 2: a critical appraisal tool for systematic reviews that include randomised or non-randomised studies of healthcare interventions, or both

6. Did the review authors perform data extraction in duplicate?

For Yes, either ONE of the following:

- ☑ at least two reviewers achieved consensus on which data to extract from included studies
- ☐ OR two reviewers extracted data from a sample of eligible studies and achieved good agreement (at least 80 percent), with the remainder extracted by one reviewer.

☑ Yes
☐ No

7. Did the review authors provide a list of excluded studies and justify the exclusions?

For Partial Yes:	For Yes, must also have:	
☑ provided a list of all potentially relevant studies that were read in full-text form but excluded from the review	☑ Justified the exclusion from the review of each potentially relevant study	☑ Yes<br>☐ Partial Yes<br>☐ No

8. Did the review authors describe the included studies in adequate detail?

For Partial Yes (ALL the following):	For Yes, should also have ALL the following:	
☑ described populations<br>☑ described interventions<br>☑ described comparators<br>☑ described outcomes<br>☑ described research designs	☑ described population in detail<br>☑ described intervention in detail (including doses where relevant)<br>☑ described comparator in detail (including doses where relevant)<br>☑ described study's setting<br>☑ timeframe for follow-up	☑ Yes<br>☐ Partial Yes<br>☐ No

9. Did the review authors use a satisfactory technique for assessing the risk of bias (RoB) in individual studies that were included in the review?

**RCTs**<br>For Partial Yes, must have assessed RoB from	For Yes, must also have assessed RoB from:	
☐ unconcealed allocation, *and*<br>☐ lack of blinding of patients and assessors when assessing outcomes (unnecessary for objective outcomes such as all-cause mortality)	☐ allocation sequence that was not truly random, *and*<br>☐ selection of the reported result from among multiple measurements or analyses of a specified outcome	☐ Yes<br>☐ Partial Yes<br>☐ No<br>☑ Includes only NRSI
**NRSI**<br>For Partial Yes, must have assessed RoB:	For Yes, must also have assessed RoB:	
☑ from confounding, *and*<br>☑ from selection bias	☑ methods used to ascertain exposures and outcomes, *and*<br>☑ selection of the reported result from among multiple measurements or analyses of a specified outcome	☑ Yes<br>☐ Partial Yes<br>☐ No<br>☐ Includes only RCTs

10. Did the review authors report on the sources of funding for the studies included in the review?

For Yes

- ☑ Must have reported on the sources of funding for individual studies included in the review. Note: Reporting that the reviewers looked for this information but it was not reported by study authors also qualifies

☑ Yes
☐ No

AMSTAR 2: a critical appraisal tool for systematic reviews that include randomised or non-randomised studies of healthcare interventions, or both

11. If meta-analysis was performed did the review authors use appropriate methods for statistical combination of results?

RCTs
For Yes:
- ☐ The authors justified combining the data in a meta-analysis
  - ☐ AND they used an appropriate weighted technique to combine study results and adjusted for heterogeneity if present.
  - ☐ AND investigated the causes of any heterogeneity

☐ Yes
☐ No
☐ No meta-analysis conducted

For NRSI
For Yes:
- ☑ The authors justified combining the data in a meta-analysis
  - ☑ AND they used an appropriate weighted technique to combine study results, adjusting for heterogeneity if present
  - ☑ AND they statistically combined effect estimates from NRSI that were adjusted for confounding, rather than combining raw data, or justified combining raw data when adjusted effect estimates were not available
  - ☑ AND they reported separate summary estimates for RCTs and NRSI separately when both were included in the review

☑ Yes
☐ No
☐ No meta-analysis conducted

12. If meta-analysis was performed, did the review authors assess the potential impact of RoB in individual studies on the results of the meta-analysis or other evidence synthesis?

For Yes:
- ☐ included only low risk of bias RCTs
- ☑ OR, if the pooled estimate was based on RCTs and/or NRSI at variable RoB, the authors performed analyses to investigate possible impact of RoB on summary estimates of effect.

☑ Yes
☐ No
☐ No meta-analysis conducted

13. Did the review authors account for RoB in individual studies when interpreting/ discussing the results of the review?

For Yes:
- ☐ included only low risk of bias RCTs
- ☑ OR, if RCTs with moderate or high RoB, or NRSI were included the review provided a discussion of the likely impact of RoB on the results

☑ Yes
☐ No

14. Did the review authors provide a satisfactory explanation for, and discussion of, any heterogeneity observed in the results of the review?

For Yes:
- ☐ There was no significant heterogeneity in the results
- ☑ OR if heterogeneity was present the authors performed an investigation of sources of any heterogeneity in the results and discussed the impact of this on the results of the review

☑ Yes
☐ No

15. If they performed quantitative synthesis did the review authors carry out an adequate investigation of publication bias (small study bias) and discuss its likely impact on the results of the review?

For Yes:
- ☑ performed graphical or statistical tests for publication bias and discussed the likelihood and magnitude of impact of publication bias

☑ Yes
☐ No
☐ No meta-analysis conducted

AMSTAR 2: a critical appraisal tool for systematic reviews that include randomised or non-randomised studies of healthcare interventions, or both

16. Did the review authors report any potential sources of conflict of interest, including any funding they received for conducting the review?

For Yes:<br>☑ The authors reported no competing interests OR<br>☐ The authors described their funding sources and how they managed potential conflicts of interest	☑ Yes<br>☐ No

To cite this tool: Shea BJ, Reeves BC, Wells G, Thuku M, Hamel C, Moran J, Moher D, Tugwell P, Welch V, Kristjansson E, Henry DA. AMSTAR 2: a critical appraisal tool for systematic reviews that include randomised or non-randomised studies of healthcare interventions, or both. BMJ. 2017 Sep 21;358:j4008.

14.2 Validation of methodological quality

Table S16. AMSTAR2 scoring system

Tool	Study design	Items																Overall rating	Score
		1	2	3	4	5	6	7	8	9	10	11	12	13	14	15	16		
AMSTAR2	Systematic review and meta-analysis																	HIGH	16

Explanation:

The methodological quality of this systematic review followed the *Assesing the Methodological Quality of Systematic Reviews-2* (AMSTAR2) recommendations and was validated using this tool. AMSTAR2 was designed to develop, evaluate and validate high quality systematic reviews through 16 items. An overall rating is obtained based on weaknesses(*) in the following critical and non-critical items (the checklist was also included in the precedent appendix page):

1. Did the research questions and inclusion criteria for the review include the components of PICO?
2. Did the report of the review contain an explicit statement that the review methods were established prior to the conduct of their review, and did the report justify any significant deviations from the protocol?*
3. Did the review authors explain their selection of the study designs for inclusion in the review?
4. Did the review authors use a comprehensive literature search strategy?*
5. Did the review authors perform study selection in duplicate?
6. Did the review authors perform data extraction in duplicate?
7. Did the review authors provide a list of excluded studies and justify the exclusions?*
8. Did the review authors describe the included studies in adequate detail?
9. Did the review authors use a satisfactory technique for assessing the risk of bias (RoB) in individual studies that were included in the review?*
10. Did the review authors report on the sources of funding for the studies included in the review?
11. If meta-analysis was performed, did the review authors use appropriate methods for statistical combination of results?*
12. If meta-analysis was performed, did the review authors assess the potential impact of RoB in individual studies on the results of the metaanalysis or other evidence synthesis?
13. Did the review authors account for RoB in individual studies when interpreting/discussing the results of the review?*
14. Did the review authors provide a satisfactory explanation for, and discussion of, any heterogeneity observed in the results of the review?
15. If they performed quantitative synthesis, did the review authors carry out an adequate investigation of publication bias (small study bias) and discuss its likely impact on the results of the review?*
16. Did the review authors report any potential sources of conflict of interest, including any funding they received for conducting the review?

High overall rating: No or one non-critical weakness. The systematic review provides an accurate and comprehensive summary of the results of the available studies that address the question of interest.

15. List of excluded studies with reasons

15.1 List S3. Records screened and excluded according to titles and abstracts

- In vitro/animal research (n=87)

Ahmed, S., Sulaiman, S. A., & Othman, N. H. (2017). Oral administration of Tualang and Manuka honeys modulates breast cancer progression in Sprague-Dawley rats model. Evidence-Based Complementary and Alternative Medicine, 2017.

An, Y., Sun, L., Derakhshan, A., Carlson, S., Chen, Z., & Waes, C. Van. (2018). Combination of birinapant and TRAILR2 agonist antibody enhances cell death in HPV-positive head and neck squamous cell carcinomas. Cancer Research, 78(13).

Bionda, C., Athias, A., Poncet, D., Alphonse, G., Guezguez, A., Gambert, P., … Ardail, D. (2008). Differential regulation of cell death in head and neck cell carcinoma through alteration of cholesterol levels in lipid rafts microdomains. Biochemical Pharmacology, 75(3), 761–772.

Boudjlida, A., Kaci, S., Karaki, S., Benayad, T., Rocchi, P., Smati, D., & Aouichat, S. B. (2019). Berberis hispanica alkaloids extract induced cell death and apoptosis in human laryngeal cancer cells Hep-2. South African Journal of Botany, 125, 134–141.

Bowman, B. M., Sebolt, K. A., Hoff, B. A., Boes, J. L., Daniels, D. L., Heist, K. A., … Galban, S. (2015). Phosphorylation of FADD by the kinase CK1alpha promotes KRASG12D-induced lung cancer. Science Signaling, 8(361), ra9–ra9.

Carlson, S., Eytan, D., Snow, G., Schiltz, S., Mohan, S., Saleh, A., … Chen, Z. (2015). Novel SMAC-mimetic birinapant demonstrates antitumor activity in human head and neck cancer models exhibiting alterations in cell death pathways. Cancer Research, 75(15).

Chen, H.-M., Liu, C.-M., Yang, H., Chou, H.-Y., Chiang, C.-P., & Kuo, M. Y.-P. (2011). 5-aminolevulinic acid induce apoptosis via NF-kappaB/JNK pathway in human oral cancer Ca9-22 cells. Journal of Oral Pathology & Medicine : Official Publication of the International Association of Oral Pathologists and the American Academy of Oral Pathology, 40(6), 483–489.

Chen, M., Zheng, Y., Song, Y., Xue, J., Liang, Z., Yan, X., & Luo, D. (2016). Pretreatment with low-dose gadolinium chloride attenuates myocardial ischemia/reperfusion injury in rats. Acta Pharmacologica Sinica, 37(4), 453–462.

Chen, Y.-P., Sivalingam, K., Shibu, M. A., Peramaiyan, R., Day, C. H., Shen, C.-Y., … Huang, C.-Y. (2019). Protective effect of Fisetin against angiotensin II-induced apoptosis by activation of IGF-IR-PI3K-Akt signaling in H9c2 cells and spontaneous hypertension rats. Phytomedicine, 57, 1–8.

Cheng, H, Yang, X., Si, H., Saleh, A., Coupar, J., Ferris, R. L., … Chen, Z. (2014). Genomic exome DNA sequencing identifies top driver genetic alterations in head and neck cancer cell lines of different HPV status. Cancer Research, 74(19).

Cheng, H, Yang, X., Si, H., Saleh, A., Coupar, J., Ferris, R. L., … Chen, Z. (2015). High throughput whole exome DNA and transcriptome RNA sequencing to identify genetic drivers and alterations in HPV-negative and HPV-positive HNSCC cell lines. Cancer Research, 75(15).

Cheng, Hui, Yang, X., Saleh, A., Cornelius, S., Waes, C. Van, Chen, Z., … Nussinov, R. (2016). RNA-seq, exome-seq, functional RNAi screening and bioinformatics analyses identify molecules promoting aberrant activation of classical and alternative NF-kB pathways in head and neck cell lines. Cancer Research, 76(14).

Cheng, Hui, Yang, X., Si, H., Saleh, A. D., Xiao, W., Coupar, J., … Chen, Z. (2018). Genomic and Transcriptomic Characterization Links Cell Lines with Aggressive Head and Neck Cancers. Cell Reports, 25(5), 1332-1345.e5.

Chun, K.-H., Pfahl, M., & Lotan, R. (2005). Induction of apoptosis by the synthetic retinoid MX3350-1 through extrinsic and intrinsic pathways in head and neck squamous carcinoma cells. Oncogene, 24(22), 3669–3677.

Derakhshan, A, Eytan, D., Snow, G., Carlson, S., Saleh, A., Cheng, H., … Waes, C. Van. (2016). Targeted therapy for head and neck squamous cell carcinoma using the novel SMAC-mimetic birinapant. Cancer Research, 76(14).

Derakhshan, Adeeb, Chen, Z., & Van Waes, C. (2017). Therapeutic Small Molecules Target Inhibitor of Apoptosis Proteins in Cancers with Deregulation of Extrinsic and Intrinsic Cell Death Pathways. Clinical Cancer Research : An Official Journal of the American Association for Cancer Research, 23(6), 1379–1387.

Ding, H., Han, C., Guo, D., Chin, Y.-W., Ding, Y., Kinghorn, A. D., & D'Ambrosio, S. M. (2009). Selective induction of apoptosis of human oral cancer cell lines by avocado extracts via a ROS-mediated mechanism. Nutrition and Cancer, 61(3), 348–356.

Du, X., Xiang, L., Mackall, C., & Pastan, I. (2011). Killing of Resistant Cancer Cells with Low Bak by a Combination of an Antimesothelin Immunotoxin and a TRAIL Receptor 2 Agonist Antibody. Clinical Cancer Research, 17(18), 5926–5934.

Eytan, D F, Snow, G. E., Carlson, S. G., Schiltz, S., Chen, Z., Waes, C. Van, & Van Waes, C. (2015). Combination effects of SMAC mimetic birinapant with TNFα, TRAIL, and docetaxel in preclinical models of HNSCC. Laryngoscope, 125(3), E118–E124.

Eytan, D F, Snow, G. E., Chen, Z., & Waes, C. Van. (2014). Novel SMAC-mimetic birinapant demonstrates preclinical antitumor activity in human xenograft head and neck cancer models exhibiting alterations in cell death pathway components FADD, BIRC2, or CASP8. Cancer Research, 74(19).

Eytan, Danielle F, Snow, G. E., Carlson, S., Derakhshan, A., Saleh, A., Schiltz, S., … Van Waes, C. (2016). SMAC Mimetic Birinapant plus Radiation Eradicates Human Head and Neck Cancers with Genomic Amplifications of Cell Death Genes FADD and BIRC2. Cancer Research, 76(18), 5442–5454.

Gajate, C., & Mollinedo, F. (2011). Lipid Rafts and Fas/CD95 Signaling in Cancer Chemotherapy. Recent Patents on Anti-Cancer Drug Discovery, 6(3), 274–283.

Guven-Maiorov, E., Keskin, O., Gursoy, A., VanWaes, C., Chen, Z., Tsai, C.-J., & Nussinov, R. (2015). The Architecture of the TIR Domain Signalosome in the Toll-like Receptor-4 Signaling Pathway. Scientific Reports, 5, 13128.

Han, B., Yao, W., Oh, Y.-T., Tong, J.-S., Li, S., Deng, J., … Sun, S.-Y. (2015). The novel proteasome inhibitor carfilzomib activates and enhances extrinsic apoptosis involving stabilization of death receptor 5. Oncotarget, 6(19), 17532–17542.

He, W., Men, J., Wang, J., Li, X., & Li, X. (2018). Xiongshao decoction ameliorates CCl4-induced liver fibrosis via inhibiting fas associated death domain (FADD)-caspase pathways. International Journal of Clinical and Experimental Medicine, 11(12), 13074–13082.

Hou, Y., Ding, X., Hou, W., Wang, T., Wang, F., Li, J., & Zeng, Y. (2014). The evaluation for anticancer activity on Hep-2 and HepG-2 cells of ribosomal protein L36 (RPL36) from the Escherichia coli BL21. Journal of Pure and Applied Microbiology, 8(2), 875–882.

Ikner, A., & Ashkenazi, A. (2011). TWEAK Induces Apoptosis through a Death-signaling Complex Comprising Receptor-interacting Protein 1 (RIP1), Fas-associated Death Domain (FADD), and Caspase-8. Journal of Biological Chemistry, 286(24), 21546–21554.

Isobe, Y., Masuda, A., Sugimoto, K., & Komatsu, N. (2016). The long form of cellular FLIP renders NK-cell lymphoma cells resistant to Fas-mediated apoptosis. Blood, 128(22).

Iulia Irimie, A., Braicu, C., Zanoaga, O., Pileczki, V., Soritau, O., Berindan-Neagoe, I., & Septimiu Campian, R. (2015). Inhibition of tumor necrosis factor alpha using RNA interference in oral squamous cell carcinoma. Journal of B.U.ON. : Official Journal of the Balkan Union of Oncology, 20(4), 1107–1114.

Iwase, M., Takaoka, S., Uchida, M., Yoshiba, S., Kondo, G., Watanabe, H., … Nagumo, M. (2008). Epidermal growth factor receptor inhibitors enhance susceptibility to Fas-mediated apoptosis in oral squamous cell carcinoma cells. Oral Oncology, 44(4), 361–368.

Iwase, M., Watanabe, H., Kondo, G., Ohashi, M., & Nagumo, M. (2003). Enhanced susceptibility of oral squamous cell carcinoma cell lines to FAS-mediated apoptosis by cisplatin and 5-fluorouracil. International Journal of Cancer, 106(4), 619–625.

Jiang, L., Wang, P., Chen, L., & Chen, H. (2014). Down-regulation of FoxM1 by thiostrepton or small interfering RNA inhibits proliferation, transformation ability and angiogenesis, and induces apoptosis of nasopharyngeal carcinoma cells. International Journal of Clinical and Experimental Pathology, 7(9), 5450–5460.

Jiang, L., Wu, X., Wang, P., Wen, T., Yu, C., Wei, L., & Chen, H. (2014). Targeting FoxM1 by thiostrepton inhibits growth and induces apoptosis of laryngeal squamous cell carcinoma. Journal of Cancer Research and Clinical Oncology, 141(6), 971–981.

Kang, N, Cao, S.-J., Zhou, Y., He, H., Tashiro, S.-I., Onodera, S., … Ikejima, T. (2015). Inhibition of caspase-9 by oridonin, a diterpenoid isolated from Rabdosia rubescens, augments apoptosis in human laryngeal cancer cells. International Journal of Oncology, 47(6), 2045–2056.

Kang, Ning, Zhang, J.-H., Qiu, F., Tashiro, S.-I., Onodera, S., & Ikejima, T. (2010). Inhibition of EGFR signaling augments oridonin-induced apoptosis in human laryngeal cancer cells via enhancing oxidative stress coincident with activation of both the intrinsic and extrinsic apoptotic pathways. Cancer Letters, 294(2), 147–158.

Katoh, M., & Katoh, M. (2003). FLJ10261 gene, located within the CCND1-EMS1 locus on human chromosome 11q13, encodes the eight-transmembrane protein homologous to C12orf3, C11orf25 and FLJ34272 gene products. International Journal of Oncology, 22(6), 1375–1381.

Katoh, M., & Katoh, M. (2005). Comparative genomics on mammalian Fgf3-Fgf4 locus. International Journal of Oncology, 27(1), 281–285.

Khan, A. P., Schinske, K. A., Nyati, S., Bhojani, M. S., Ross, B. D., & Rehemtulla, A. (2010). High-Throughput Molecular Imaging for the Identification of FADD Kinase Inhibitors. Journal of Biomolecular Screening, 15(9), 1063–1070.

Kim, J. H., Ajaz, M., Lokshin, A., & Lee, Y. J. (2003). Role of antiapoptotic proteins in tumor necrosis factor-related apoptosis-inducing ligand and cisplatin-augmented apoptosis. Clinical Cancer Research : An Official Journal of the American Association for Cancer Research, 9(8), 3134–3141.

Kim, Y.-H., & Lee, Y. J. (2006). Time sequence of tumor necrosis factor-related apoptosis-inducing ligand (TRAIL) and cisplatin treatment is responsible for a complex pattern of synergistic cytotoxicity. Journal of Cellular Biochemistry, 98(5), 1284–1295.

Kondo, G., Iwase, M., Watanabe, H., Uchida, M., Takaoka, S., Ohashi, M., & Nagumo, M. (2006). Enhancement of susceptibility to Fas-mediated apoptosis in oral squamous cell carcinoma cells by phosphatidylinositol 3-kinase inhibitor. Oral Oncology, 42(7), 745–752.

Li, B., Ren, H., Yue, P., Chen, M., Khuri, F. R., & Sun, S.-Y. (2012). The Novel Akt Inhibitor API-1 Induces c-FLIP Degradation and Synergizes with TRAIL to Augment Apoptosis Independent of Akt Inhibition. Cancer Prevention Research, 5(4), 612–620.

Li, C., Egloff, A. M., Sen, M., Grandis, J. R., & Johnson, D. E. (2014). Caspase-8 mutations in head and neck cancer confer resistance to death receptor-mediated apoptosis and enhance migration, invasion, and tumor growth. Molecular Oncology, 8(7), 1220–1230.

Li, P., Li, S., Yin, D., Li, J., Wang, L., Huang, C., & Yang, X. (2017). EGCG sensitizes human nasopharyngeal carcinoma cells to TRAIL-mediated apoptosis by activation NF-kappaB. Neoplasma, 64(1), 74–80.

Li, S.-S., Yang, S., Wang, S., Yang, X.-M., Tang, Q.-L., & Wang, S.-H. (2011). Latent membrane protein 1 mediates the resistance of nasopharyngeal carcinoma cells to TRAIL-induced apoptosis by activation of the PI3K/Akt signaling pathway. Oncology Reports, 26(6), 1573–1579.

Li, X.-T. (2016). Laryngeal squamous cell carcinoma progression is associated with the NFκB signaling pathway regulated by IκB kinaseβ. European Review for Medical and Pharmacological Sciences, 20(3), 467–475.

Li, X., Huang, Q., Ong, C.-N., Yang, X.-F., & Shen, H.-M. (2010). Chrysin sensitizes tumor necrosis factor-alpha-induced apoptosis in human tumor cells via suppression of nuclear factor-kappaB. Cancer Letters, 293(1), 109–116.

Lin, H.-F., Hsieh, M.-J., Hsi, Y.-T., Lo, Y.-S., Chuang, Y.-C., Chen, M.-K., & Chien, S. Y. (2017). Celastrol-induced apoptosis in human nasopharyngeal carcinoma is associated with the activation of the death receptor and the mitochondrial pathway. Oncology Letters, 14(2), 1683–1690.

Lin, X.-Y., He, C.-D., Xiao, T., Jin, X., Chen, J., Wang, Y.-K., … Chen, H.-D. (2009). Acitretin induces apoptosis through CD95 signalling pathway in human cutaneous squamous cell carcinoma cell line SCL-1. Journal of Cellular and Molecular Medicine, 13(9), 2888–2898.

Liu, D.-Q., Situ, Z.-Q., Cao, Y.-X., Yu, C.-J., Wang, C.-J., & Yang, A.-G. (2004). Identification of construction and biological characters of tongue carcinoma cell line T-TFL. Chinese Journal of Clinical Rehabilitation, 8(11), 2174–2175.

Liu, P.-Y.-Y., Chang, D.-C.-C., Lo, Y.-S.-S., Hsi, Y.-T.-T., Lin, C.-C.-C., Chuang, Y.-C.-C., … Chen, M.-K.-K. (2018). Osthole induces human nasopharyngeal cancer cells apoptosis through Fasâ€"Fas ligand and mitochondrial pathway. Environmental Toxicology, 33(4), 446–453.

Lokeshwar, V. B., Lopez, L. E., Munoz, D., Chi, A., Shirodkar, S. P., Lokeshwar, S. D., … Altman, N. (2010). Antitumor activity of hyaluronic acid synthesis inhibitor 4-methylumbelliferone in prostate cancer cells. Cancer Research, 70(7), 2613–2623.

Ma, Y.-S., Yao, C.-N., Liu, H.-C., Yu, F.-S., Lin, J.-J., Lu, K.-W., … Chung, J.-G. (2018). Quercetin induced apoptosis of human oral cancer SAS cells through mitochondria and endoplasmic reticulum mediated signaling pathways. Oncology Letters, 15(6), 9663–9672.

McDonald III, E. R., Chui, P. C., Martelli, P. F., Dicker, D. T., El-Deiry, W. S., McDonald, E. R. 3rd, … El-Deiry, W. S. (2001). Death Domain Mutagenesis of KILLER/DR5 Reveals Residues Critical for Apoptotic Signaling. Journal of Biological Chemistry, 276(18), 14939–14945.

Min, S.-K., Jung, S. Y., Kang, H. K., Park, S.-A., Lee, J. H., Kim, M.-J., & Min, B.-M. (2017). Functional diversity of MIR-146a-5p and TRAF6 in normal and oral cancer cells. International Journal of Oncology, 51(5), 1541–1552.

Mishima, K., Nariai, Y., & Yoshimura, Y. (2003). Carboplatin induces Fas (APO-1/CD95)-dependent apoptosis of human tongue carcinoma cells: sensitization for apoptosis by upregulation of FADD expression. International Journal of Cancer, 105(5), 593–600.

Mishima, K., Nariai, Y., & Yoshimura, Y. (2005). Etodolac, a selective cyclo-oxygenase-2 inhibitor, enhances carboplatin-induced apoptosis of human tongue carcinoma cells by down-regulation of FAP-1 expression. Oral Oncology, 41(1), 77–81.

Ndour, A., Brocqueville, G., Ouk, T., Goormachtig, G., Mougel, A., Feuillard, J., … Adriaenssens, E. (2009). Development of inhibiting peptides derived from the latent membrane protein1 (LMP1) encoded by the epstein - Barr virus (EBV) presenting anti-proliferative and pro-apoptotic properties. European Journal of Immunology, 39, S195–S195.

O'Brian, C. A., Stewart, J. R., & Chu, F. (2005). Cancer: Chemoprotective activity of stilbenes: Resveratrol. Nutrition and Cancer Prevention, 369–384.

Oz, H. S., & Ebersole, J. L. (2010). Green tea polyphenols mediated apoptosis in intestinal epithelial cells by a fadd-dependent pathway. Journal of Cancer Therapy, 1(3), 105–113.

Pan, S.-T., Qin, Y., Zhou, Z.-W., He, Z.-X., Zhang, X., Yang, T., … Qiu, J.-X. (2015). Plumbagin suppresses epithelial to mesenchymal transition and stemness via inhibiting Nrf2-mediated signaling pathway in human tongue squamous cell carcinoma cells. Drug Design, Development and Therapy, 9, 5511–5551.

Park, M A, Zhang, G., Martin, A. P., Hamed, H., Mitchell, C., Hylemon, P. B., … Dent, P. (2008). Vorinostat and sorafenib increase ER stress, autophagy and apoptosis via ceramide-dependent CD95 and PERK activation. Cancer Biology and Therapy, 7(10), 1648–1662.

Park, Margaret A, Zhang, G., Mitchell, C., Rahmani, M., Hamed, H., Hagan, M. P., … Dent, P. (2008). Mitogen-activated protein kinase kinase 1/2 inhibitors and 17-allylamino-17-demethoxygeldanamycin synergize to kill human gastrointestinal tumor cells in vitro via suppression of c-FLIP-s levels and activation of CD95. Molecular Cancer Therapeutics, 7(9), 2633–2648.

Philion, C., Ma, D., Ruvinov, I., Mansour, F., Pignanelli, C., Noel, M., … Pandey, S. (2017). Cymbopogon citratus and Camellia sinensis extracts selectively induce apoptosis in cancer cells and reduce growth of lymphoma xenografts in vivo. Oncotarget, 8(67), 110756–110773.

Ramaswamy, M., Efimova, E. V, Martinez, O., Mulherkar, N. U., Singh, S. P., & Prabhakar, B. S. (2004). IG20 (MADD splice variant-5), a proapoptotic protein, interacts with DR4/DR5 and enhances TRAIL-induced apoptosis by increasing recruitment of FADD and caspase-8 to the DISC. Oncogene, 23(36), 6083–6094.

Selimovic, D., Wahl, R. U., Ruiz, E., Aslam, R., Flanagan, T. W., Hassan, S.-Y., … Hassan, M. (2019). Tumor necrosis factor-alpha triggers opposing signals in head and neck squamous cell carcinoma and induces apoptosis via mitochondrial- and non-mitochondrial-dependent pathways. International Journal of Oncology, 55(6), 1324–1338.

Seo, S. U., Cho, H. K., Min, K.-J., Woo, S. M., Kim, S., Park, J.-W., … Kwon, T. K. (2017). Thioridazine enhances sensitivity to carboplatin in human head and neck cancer cells through downregulation of c-FLIP and Mcl-1 expression. Cell Death & Disease, 8(2), e2599–e2599.

Su, E.-Y., Chu, Y.-L., Chueh, F.-S., Ma, Y.-S., Peng, S.-F., Huang, W.-W., … Chung, J.-G. (2019). Bufalin Induces Apoptotic Cell Death in Human Nasopharyngeal Carcinoma Cells through Mitochondrial ROS and TRAIL Pathways. The American Journal of Chinese Medicine, 47(1), 237–257.

Sulkshane, P., & Teni, T. (2017). BH3 mimetic Obatoclax (GX15-070) mediates mitochondrial stress predominantly via MCL-1 inhibition and induces autophagy-dependent necroptosis in human oral cancer cells. Oncotarget, 8(36), 60060–60079.

Takashina, T., & Nakayama, M. (2007). Modifications enhance the apoptosis-inducing activity of FADD. Molecular Cancer Therapeutics, 6(6), 1793–1803.

Wang, G. D., Huang, J. T., Liu, Y., Wu, Y., Chen, X. Q., Zhao, Y. F., & Wang, D. L. (2019). Deletion of C9orf53 promotes the growth of head and neck squamous cell carcinoma and is associated with poor prognosis of patients with head and neck squamous cell carcinoma. Oncology Letters, 17(1), 1223–1228.

Wang, J., Huang, H., Wang, C., Liu, X., Hu, F., & Liu, M. (2013). MicroRNA-375 sensitizes tumour necrosis factor-alpha (TNF-alpha)-induced apoptosis in head and neck squamous cell carcinoma in vitro. International Journal of Oral and Maxillofacial Surgery, 42(8), 949–955.

Wieckowski, E., Atarashi, Y., Stanson, J., Sato, T.-A., & Whiteside, T. L. (2007). FAP-1-mediated activation of NF-kappaB induces resistance of head and neck cancer to Fas-induced apoptosis. Journal of Cellular Biochemistry, 100(1), 16–28.

Wu, S.-Y., Wu, A. T. H., & Liu, S.-H. (2016). MicroRNA-17-5p regulated apoptosis-related protein expression and radiosensitivity in oral squamous cell carcinoma caused by betel nut chewing. Oncotarget, 7(32), 51482–51493.

Xiao, R, Allen, C., Tran, L., Park, S.-J., Chen, Z., Waes, C. Van, & Schmitt, N. (2017). Dual cIAP1/XIAP inhibitor ASTX660 synergizes with radiation therapy and PD-1 blockade to enhance anti-tumor immunity. Journal for ImmunoTherapy of Cancer, 5.

Xiao, R, An, Y., Derakhshan, A., Chen, Z., Schmitt, N. C., & Waes, C. Van. (2017). Novel dual cIAP1/XIAP antagonist ASTX660 activity in preclinical models of human papillomavirus(+) and (-) head and neck squamous cell carcinoma. Cancer Research, 77(13).

Xiao, Roy, Allen, C. T., Tran, L., Patel, P., Park, S.-J., Chen, Z., … Schmitt, N. C. (2018). Antagonist of cIAP1/2 and XIAP enhances anti-tumor immunity when combined with radiation and PD-1 blockade in a syngeneic model of head and neck cancer. Oncoimmunology, 7(9), e1471440–e1471440.

Xiao, Y., Volk, A., Zhang, S., Wei, W., Breslin, P., & Zhang, J. (2011). TNF-Î±/Fas-RIP-1-induced cell death signaling separates hematopoietic stem cells/progenitors into two distinct populations. Blood, 118(21).

Yang, L., Kumar, B., Shen, C., Zhao, S., Blakaj, D., Li, T., … Williams, T. M. (2019). LCL161, a SMAC-mimetic, preferentially radiosensitizes human papillomavirus-negative head and neck squamous cell carcinoma. Molecular Cancer Therapeutics, 18(6), 1025–1035.

Yang, X., Cheng, H., Saleh, A., Cornelius, S., Guven-Maiorov, E., Nussinov, R., … Gursoy, A. (2015). Altered inflammatory and death pathways in head and neck cell lines model genomic and expression signatures identified in The Cancer Genome Atlas. Cancer Research, 75(15).

Yang, X., Cheng, H., Si, H., Saleh, A., Maiorov, E. G., Coupar, J., … Chen, Z. (2014). Genome-wide RNA and DNA high throughput sequencing reveals proinflammatory and death gene signatures in head and neck squamous cell carcinoma lines with different HPV status. Cancer Research, 74(19).

Yasuda, M., Kato, S., Yamanaka, N., Iimori, M., Utsumi, D., Kitahara, Y., … Takeuchi, K. (2012). Potential role of the NADPH oxidase NOX1 in the pathogenesis of 5-fluorouracil-induced intestinal mucositis in mice. American Journal of Physiology-Gastrointestinal and Liver Physiology, 302(10), G1133–G1142.

Yuan, C.-H., Filippova, M., Tungteakkhun, S. S., Duerksen-Hughes, P. J., & Krstenansky, J. L. (2012). Small molecule inhibitors of the HPV16-E6 interaction with caspase 8. Bioorganic & Medicinal Chemistry Letters, 22(5), 2125–2129.

Zhang, H., Li, X., Zhang, Y., & Luan, X. (2014). Luteolin induces apoptosis by activating Fas signaling pathway at the receptor level in laryngeal squamous cell line Hep-2 cells. European Archives of Oto-Rhino-Laryngology : Official Journal of the European Federation of Oto-Rhino-Laryngological Societies (EUFOS) : Affiliated with the German Society for Oto-Rhino-Laryngology - Head and Neck Surgery, 271(6), 1653–1659.

Zhang, L., Ren, X., Alt, E., Bai, X., Huang, S., Xu, Z., … Wu, X. (2010). Chemoprevention of colorectal cancer by targeting APC-deficient cells for apoptosis. Nature, 464(7291), 1058–1061.

Zhang, Z., Yu, H.-J., Huang, H., Wang, H.-H., Wu, S., Liu, H.-Y., & Zhang, H.-T. (2019). The photodynamic activity and toxicity evaluation of 5,10,15-tris(ethoxylcarbonyl)corrole phosphorus(V) in vivo and in vitro. European Journal of Medicinal Chemistry, 163, 779–786.

Zhao, L., Yue, P., Lonial, S., Khuri, F. R., & Sun, S.-Y. (2011). The NEDD8-activating enzyme inhibitor, MLN4924, cooperates with TRAIL to augment apoptosis through facilitating c-FLIP degradation in head and neck cancer cells. Molecular Cancer Therapeutics, 10(12), 2415–2425.

- No HNSCC (n=56)

Abedini, M. R., Wang, P.-W., Huang, Y.-F., Cao, M., Chou, C.-Y., Shieh, D.-B., & Tsang, B. K. (2014). Cell fate regulation by gelsolin in human gynecologic cancers. Proceedings of the National Academy of Sciences of the United States of America, 111(40), 14442–14447.

Anees, M., Horak, P., Schiefer, A.-I., VaÅˆhara, P., El-Gazzar, A., Perco, P., … Krainer, M. (2015). The potential evasion of immune surveillance in mucosa associated lymphoid tissue lymphoma by DcR2-mediated up-regulation of nuclear factor-ÎºB. Leukemia and Lymphoma, 56(5), 1440–1449.

Basu, A., Castle, V. P., Bouziane, M., Bhalla, K., & Haldar, S. (2006). Crosstalk between extrinsic and intrinsic cell death pathways in pancreatic cancer: Synergistic action of estrogen metabolite and ligands of death receptor family. Cancer Research, 66(8), 4309–4318.

Bhattacharyya, S., Mandal, D., Sen, G. S., Pal, S., Banerjee, S., Lahiry, L., … Sa, G. (2007). Tumor-induced oxidative stress perturbs nuclear factor-ÎºB activity-augmenting tumor necrosis factor-Î±-mediated T-cell death: Protection by curcumin. Cancer Research, 67(1), 362–370.

Chang, M. S., Lee, H. S., Lee, B. L., Kim, Y. T., Lee, J. S., & Kim, W. H. (2005). Differential protein expression between esophageal squamous cell carcinoma and dysplasia, and prognostic significance of protein markers. Pathology Research and Practice, 201(6), 417–425.

Chatterjee, S., Patra, D., Chakraborti, U., Sengupta, D., Ghosh, P., Basu, A., … Chowdhury, K. D. (2019). Association of p38MAPK-p53-Fas aggregation in S-allyl cysteine mediated regulation of hepatocarcinoma. Environmental Toxicology, 34(8), 928–940.

Chen, K.-F., Tai, W.-T., Liu, T.-H., Huang, H.-P., Lin, Y.-C., Shiau, C.-W., … Cheng, A.-L. (2010). Sorafenib overcomes TRAIL resistance of hepatocellular carcinoma cells through the inhibition of STAT3. Clinical Cancer Research, 16(21), 5189–5199.

Chen, Y.-J., Chi, C.-W., Su, W.-C., & Huang, H.-L. (2014). Lapatinib induces autophagic cell death and inhibits growth of human hepatocellular carcinoma. Oncotarget, 5(13), 4845–4854.

Choi, E. J., Yun, J. A., Jabeen, S., Jeon, E. K., Won, H. S., Ko, Y. H., & Kim, S. Y. (2014). Prognostic significance of TMEM16A, PPFIA1, and FADD expression in invasive ductal carcinoma of the breast. World Journal of Surgical Oncology, 12, 137.

Chou, T.-H., & Liang, C.-H. (2009). The molecular effects of aloe-emodin (AE)/liposome-AE on human nonmelanoma skin cancer cells and skin permeation. Chemical Research in Toxicology, 22(12), 2017–2028.

Das, S., Tripathi, N., Siddharth, S., Nayak, A., Nayak, D., Sethy, C., … Kundu, C. N. (2017). Etoposide and doxorubicin enhance the sensitivity of triple negative breast cancers through modulation of TRAIL-DR5 axis. Apoptosis, 22(10), 1205–1224.

Fatehchand, K., Santhanam, R., Shen, B., Erickson, E. L., Gautam, S., Elavazhagan, S., … Butchar, J. P. (2017). Active hexose-correlated compound enhances extrinsic-pathway-mediated apoptosis of Acute Myeloid Leukemic cells. PLoS ONE, 12(7).

Ferreira, C. G., Span, S. W., Peters, G. J., Kruyt, F. A. E., & Giaccone, G. (2000). Chemotherapy triggers apoptosis in a caspase-8-dependent and mitochondria-controlled manner in the non-small cell lung cancer cell line NCI-H460. Cancer Research, 60(24), 7133–7141.

Fulda, S. (2011). Targeting apoptosis signaling in pancreatic cancer. Cancers, 3(1), 241–251

Ganapathy, M., Ghosh, R., Jianping, X., Zhang, X., Bedolla, R., Schoolfield, J., … Kumar, A. P. (2009). Involvement of FLIP in 2-methoxyestradiol-induced tumor regression in transgenic adenocarcinoma of mouse prostate model. Clinical Cancer Research, 15(5), 1601–1611.

Garcia-Roig, M., Yates, T., Benitez, A., & Lokeshwar, V. (2012). 4-methylumbeliferone: A potential anti-metastatic oral agent for bladder cancer. Journal of Urology, 187(4), e364–e364.

Giannopoulos, K, Giannopoulos, K., Dmoszynska, A., Kowal, M., Wasik-Szczepanek, E. W. K., Wlasiuk, P., … Bullinger, L. (2008). Combined Thalidomide and Fludarabine Therapy in Chronic Lymphocytic Leukemia Results in Distinct Molecular and Antileukemic Effects. Haematologica-the Hematology Journal, 93, 33–34.

Giannopoulos, Krzysztof, Dmoszynska, A., Kowal, M., Wasik-Szczepanek, E., Wlasiuk, P., Bojarska-Junak, A., … Bullinger, L. (2007). Thalidomide alone and in combination with fludarabine exerts distinct molecular and antileukemic effects in B-cell chronic lymphocytic leukemia. Blood, 110(11), 919A-919A.

Gravina, G. L., Marampon, F., Staso, M. D., Bonfili, P., Vitturini, A., Jannini, E. A., … Festuccia, C. (2010). 5-Azacitidine restores and amplifies the bicalutamide response on preclinical models of androgen receptor expressing or deficient prostate tumors. Prostate, 70(11), 1166–1178.

Gregory-Evans, C. Y., Moosajee, M., Hodges, M. D., Mackay, D. S., Game, L., Vargesson, N., … Gregory-Evans, K. (2007). SNP genome scanning localizes oto-dental syndrome to chromosome 11q13 and microdeletions at this locus implicate FGF3 in dental and inner-ear disease and FADD in ocular coloboma. Human Molecular Genetics, 16(20), 2482–2493.

He, K., Chen, D., Ruan, H., Li, X., Tong, J., Xu, X., … Yu, J. (2016). BRAFV600E-dependent Mcl-1 stabilization leads to everolimus resistance in colon cancer cells. Oncotarget, 7(30), 47699–47710.

He, K., Zheng, X., Li, M., Zhang, L., & Yu, J. (2016). mTOR inhibitors induce apoptosis in colon cancer cells via CHOP-dependent DR5 induction on 4E-BP1 dephosphorylation. Oncogene, 35(2), 148–157.

Huang, S., Ren, X., Wang, L., Zhang, L., & Wu, X. (2011). Lung-cancer chemoprevention by induction of synthetic lethality in mutant KRAS premalignant cells in vitro and in vivo. Cancer Prevention Research (Philadelphia, Pa.), 4(5), 666–673.

Jedema, I., Dreunen, L. Van, Hagedoorn, R. S., & Falkenburg, J. H. F. (2010). Multi-dimensional resistance phenotype allows subpopulation of quiescent chronic myeloid leukemia stem cells to universally escape from therapeutic attack. Blood, 116(21).

Jeng, Y. M., & Hsu, H. C. (2002). Mutation of the DR5/TRAIL receptor 2 gene is infrequent in hepatocellular carcinoma. Cancer Letters, 181(2), 205–208.

Jeon, Y. K., Kim, H., Park, S. O., Choi, H. Y., Kim, Y. A., Park, S.-S., … Kim, C.-W. (2005). Resistance to Fas-mediated apoptosis is restored by cycloheximide through the downregulation of cellular FLIPL in NK/T-cell lymphoma. Laboratory Investigation; a Journal of Technical Methods and Pathology, 85(7), 874–884.

Jesse, P., Riepl, H., Wieder, T., Henze, G., & Prokop, A. (2008). A Synthetic Analogue of the Indigo Jeans Dye Tryptanthrin, NT1, Induces Apoptosis in Vitro, Ex Vivo and in Vivo in CD95-Dependent Manner in Pediatric Leukemia and Lymphoma Cells Sensitizing Malignant Cells to Cytostatic Drugs Via Downregulation of XIAP. Blood, 112(11), 910.

Jiao, C., Chen, W., Tan, X., Liang, H., Li, J., Yun, H., … Yang, B. B. (2020). Ganoderma lucidum spore oil induces apoptosis of breast cancer cells in vitro and in vivo by activating caspase-3 and caspase-9. Journal of Ethnopharmacology, 247.

Jin, F., Liu, X., Zhou, Z., Yue, P., Lotan, R., Khuri, F. R., … Sun, S.-Y. (2005). Activation of nuclear factor-ÎoB contributes to induction of death receptors and apoptosis by the synthetic retinoid CD437 in DU145 human prostate cancer cells. Cancer Research, 65(14), 6354–6363.

Jin, G., Liu, Y., Xu, P., & Jin, G. (2019). Induction of necroptosis in human breast cancer drug-resistant cells by SMAC analog LCL161 after caspase inhibition requires RIP3. Pharmazie, 74(6), 363–368.

Kappers, R., Yahalom, J., & Josting, A. (2006). Advances in biology, diagnostics, and treatment of Hodgkin's disease. Biology of Blood and Marrow Transplantation, 12, 66–76.

Koshkina, N. V, Rao-Bindal, K., & Kleinerman, E. S. (2011). Effect of the histone deacetylase inhibitor SNDX-275 on Fas signaling in osteosarcoma cells and the feasibility of its topical application for the treatment of osteosarcoma lung metastases. Cancer, 117(15), 3457–3467.

Liang, C.-H., Wang, G.-H., Chou, T.-H., Wang, S.-H., Lin, R.-J., Chan, L.-P., … Sheu, J.-H. (2012). 5-epi-Sinuleptolide induces cell cycle arrest and apoptosis through tumor necrosis factor/mitochondria-mediated caspase signaling pathway in human skin cancer cells. Biochimica et Biophysica Acta - General Subjects, 1820(7), 1149–1157.

Masuda, A., Isobe, Y., Sugimoto, K., Yoshimori, M., Arai, A., & Komatsu, N. (2020). Efficient recruitment of c-FLIPL to the death-inducing signaling complex leads to Fas resistance in natural killer-cell lymphoma. Cancer Science, 111(3), 807–816.

Pan, P.-J., Liu, Y.-C., & Hsu, F.-T. (2019). Protein Kinase B and Extracellular Signal-Regulated Kinase Inactivation is Associated with Regorafenib-Induced Inhibition of Osteosarcoma Progression In Vitro and In Vivo. Journal of Clinical Medicine, 8(6).

Paoluzzi, L., & O'Connor, O. A. (2010). Targeting survival pathways in lymphoma. Advances in Experimental Medicine and Biology, 687, 79–96.

Patel, S., Murphy, D., Haralambieva, E., Abdulla, Z. A., Wong, K. K., Chen, H., … Pulford, K. (2014). Increased expression of phosphorylated FADD in anaplastic large cell and other t-cell lymphomas. Biomarker Insights, 9, 77–84.

Rao-Bindal, K, & Kleinerman, E. S. (2011). The histone deacetylase inhibitor MS-275 sensitizes osteosarcoma cells and osteosarcoma lung metastases to FasL-induced cell death by the downregulation c-FLIP. Cancer Research, 71(8).

Rao-Bindal, Krithi, Koshkina, N. V, Stewart, J., & Kleinerman, E. S. (2013). The histone deacetylase inhibitor, MS-275 (entinostat), downregulates c-FLIP, sensitizes osteosarcoma cells to FasL, and induces the regression of osteosarcoma lung metastases. Current Cancer Drug Targets, 13(4), 411–422.

Rho, J., Seo, C.-S., Park, H.-S., Wijerathne, C. U. B., Jeong, H.-Y., Moon, O.-S., … Kwun, H.-J. (2019). Ulmus macrocarpa Hance improves benign prostatic hyperplasia by regulating prostatic cell apoptosis. Journal of Ethnopharmacology, 233, 115–122.

Riches, J. C., Sangaralingam, A., Kiaii, S., Chaplin, T., Cekdemir, D., Iqbal, S., … Gribben, J. G. (2011). Impact of lenalidomide on gene expression profiles of malignant and immune cells in patients with chronic lymphocytic leukemia. Blood, 118(21).

Roy, K., Kanwar, R. K., & Kanwar, J. R. (2015). LNA aptamer based multi-modal, Fe3O4-saturated lactoferrin (Fe3O4-bLf) nanocarriers for triple positive (EpCAM, CD133, CD44) colon tumor targeting and NIR, MRI and CT imaging. Biomaterials, 71, 84–99.

Shamaladevi, N., Araki, S., Lyn, D. A., Ayyathurai, R., Gao, J., Lokeshwar, V. B., … Lokeshwar, B. L. (2016). The andean anticancer herbal product BIRM causes destabilization of androgen receptor and induces caspase-8 mediated-apoptosis in prostate cancer. Oncotarget, 7(51), 84201–84213.

Shankar, S., Chen, Q., Ganapathy, S., Singh, K. P., & Srivastava, R. K. (2008). Diallyl trisulfide increases the effectiveness of TRAIL and inhibits prostate cancer growth in an orthotopic model: molecular mechanisms. Molecular Cancer Therapeutics, 7(8), 2328–2338.

Shi, B., Wang, L.-F., Meng, W.-S., Chen, L., & Meng, Z.-L. (2017). Carnosic acid and fisetin combination therapy enhances inhibition of lung cancer through apoptosis induction. International Journal of Oncology, 50(6), 2123–2135.

Tavallai, S., Hamed, H. A., Grant, S., Poklepovic, A., & Dent, P. (2014). Pazopanib and HDAC inhibitors interact to kill sarcoma cells. Cancer Biology and Therapy, 15(5), 578–585.

Tungteakkhun, S. S., Filippova, M., Fodor, N., & Duerksen-Hughes, P. J. (2010). The full-length isoform of human papillomavirus 16 E6 and its splice variant E6* bind to different sites on the procaspase 8 death effector domain. Journal of Virology, 84(3), 1453–1463.

Wang, X., Xue, Q., Wu, L., Wang, B., & Liang, H. (2018). Dasatinib promotes TRAIL-mediated apoptosis by upregulating CHOP-dependent death receptor 5 in gastric cancer. FEBS Open Bio, 8(5), 732–742.

Won, K.-J., Chung, K.-S., Lee, Y. S., Alia, M. S., Pervez, M. K., Fatima, S., … Lee, K.-T. (2010). Haplophytin-A induces caspase-8-mediated apoptosis via the formation of death-inducing signaling complex in human promyelocytic leukemia HL-60 cells. Chemico-Biological Interactions, 188(3), 505–511.

Yang, S.-J., Lee, S. A., Park, M.-G., Kim, J.-S., Yu, S.-K., Kim, C. S., … Kim, D. K. (2014). Induction of apoptosis by diphenyldifluoroketone in osteogenic sarcoma cells is associated with activation of caspases. Oncology Reports, 31(5), 2286–2292.

Yu, X., Ling, J., Liu, X., Guo, S., Lin, Y., Liu, X., & Su, L. (2017). Cordycepin induces autophagy-mediated c-FLIPL degradation and leads to apoptosis in human non-small cell lung cancer cells. Oncotarget, 8(4), 6691–6699.

Zakaria, Z., Tivnan, A., Flanagan, L., Murray, D. W., Salvucci, M., Stringer, B. W., … Prehn, J. H. M. (2016). Patient-derived glioblastoma cells show significant heterogeneity in treatment responses to the inhibitor-of-apoptosis-protein antagonist birinapant. British Journal of Cancer, 114(2), 188–198.

Zeise, E., Weichenthal, M., Schwarz, T., & Kulms, D. (2004). Resistance of human melanoma cells against the death ligand TRAIL is reversed by ultraviolet-B radiation via downregulation of FLIP. Journal of Investigative Dermatology, 123(4), 746–754.

Zhang, H., Chen, G., Wang, Y., Zhuang, N., Wang, S., & Shi, L. (2015). Hepatitis B virus x protein sensitizes trail induced hepatocyte apoptosis by inhibiting E3 ubiquitin ligase A20. Hepatology International, 9(1), S202–S202.

Zhang, M., Su, L., Xiao, Z., Liu, X., & Liu, X. (2016). Methyl jasmonate induces apoptosis and pro-apoptotic autophagy via the ROS pathway in human non-small cell lung cancer. American Journal of Cancer Research, 6(2), 187–199.

Zhang, T., Wang, X., He, D., Jin, X., & Guo, P. (2014). Metformin sensitizes human bladder cancer cells to TRAIL-induced apoptosis through mTOR/S6K1-mediated downregulation of c-FLIP. Anti-Cancer Drugs, 25(8), 887–897.

- Off topic (n=36)

Alkurdi, L., Virard, F., Vanbervliet, B., Weber, K., Toscano, F., Bonnin, M., … Estornes, Y. (2018). Release of c-FLIP brake selectively sensitizes human cancer cells to TLR3-mediated apoptosis. Cell Death & Disease, 9, 874.

Ando, M., Kawazu, M., Ueno, T., Fukumura, K., Yamato, A., Soda, M., … Mano, H. (2013). Cancer-associated missense mutations of caspase-8 activate nuclear factor-kappa B signaling. Cancer Science, 104(8), 1002–1008.

Belz, K., Schoeneberger, H., Wehner, S., Weigert, A., Basnig, H., Klingebiel, T., … Fulda, S. (2014). Smac mimetic and glucocorticoids synergize to induce apoptosis in childhood ALL by promoting ripoptosome assembly. Blood, 124(2), 240–250.

Bernal-Mizrachi, L., Cole, C. E., Heffner, L., Nooka, A. K., Vij, R., Kelkar, N., … Kaufman, J. L. (2018). Phase II Trial of Ixazomib and Dexamethasone Versus Ixazomib, Dexamethasone and Lenalidomide, Randomized with NFKB2 Rearrangement. (Proteasome Inhibitor NFKB2 Rearrangement Driven Trial, PINR). Blood, 132.

Booth, L., Roberts, J. L., Cruickshanks, N., Tavallai, S., Webb, T., Samuel, P., … Dent, P. (2015). PDE5 inhibitors enhance celecoxib killing in multiple tumor types. Journal of Cellular Physiology, 230(5), 1115–1127.

Cen, X., Liu, S., & Cheng, K. (2018). The role of toll-like receptor in inflammation and tumor immunity. Frontiers in Pharmacology, 9.

Chen, L.-C., Chung, I.-C., Hsueh, C., Tsang, N.-M., Chi, L.-M., Liang, Y., … Chang, Y.-S. (2010). The antiapoptotic protein, FLIP, is regulated by heterogeneous nuclear ribonucleoprotein K and correlates with poor overall survival of nasopharyngeal carcinoma patients. Cell Death and Differentiation, 17(9), 1463–1473.

Chen, S., Rehman, S. K., Zhang, W., Wen, A., Yao, L., & Zhang, J. (2010). Autophagy is a therapeutic target in anticancer drug resistance. Biochimica et Biophysica Acta - Reviews on Cancer, 1806(2), 220–229.

Chhabra, R., Adlakha, Y. K., Hariharan, M., Scaria, V., & Saini, N. (2009). Upregulation of miR-23a similar to 27a similar to 24-2 Cluster Induces Caspase-Dependent and -Independent Apoptosis in Human Embryonic Kidney Cells. Plos One, 4(6), e5848–e5848.

Dkhil, M. A., Moneim, A. E. A., Hafez, T. A., Mubaraki, M. A., Mohamed, W. F., Thagfan, F. A., & Al-Quraishy, S. (2019). Myristica fragrans Kernels Prevent Paracetamol-Induced Hepatotoxicity by Inducing Anti-Apoptotic Genes and Nrf2/HO-1 Pathway. International Journal of Molecular Sciences, 20(4), 993.

Endo, T., Kusakabe, M., Sunadome, K., Yamamoto, T., & Nishida, E. (2011). The Kinase SGK1 in the Endoderm and Mesoderm Promotes Ectodermal Survival by Down-Regulating Components of the Death-Inducing Signaling Complex. Science Signaling, 4(156), ra2–ra2.

Filippova, M., Filippov, V. A., Kagoda, M., Garnett, T., Fodor, N., & Duerksen-Hughes, P. J. (2009). Complexes of Human Papillomavirus Type 16 E6 Proteins Form Pseudo-Death-Inducing Signaling Complex Structures during Tumor Necrosis Factor-Mediated Apoptosis. Journal of Virology, 83(1), 210–227.

Ghareeb, M., Naser, H., El-Gammal, M., Zeeneldin, A., Bahnacy, A., & Khaled, H. (2016). Metformin and response to neoadjuvant chemotherapy in patients with breast cancer. Annals of Oncology, 27.

Gomez, B., Carter, L., Trustrum, N. A., Page, M. J., & Orpin, A. R. (2013). Coherent rainfall response to middle- and late-Holocene climate variability across the mid-latitude South Pacific. Holocene, 23(7), 1002–1007.

Horai, Y., Nakamura, H., Nakashima, Y., Hayashi, T., & Kawakami, A. (2016). Analysis of the downstream mediators of toll-like receptor 3-induced apoptosis in labial salivary glands in patients with Sjogren's syndrome. Modern Rheumatology, 26(1), 99–104.

Kiesslich, T., Krammer, B., & Plaetzer, K. (2006). Cellular mechanisms and prospective applications of hypericin in photodynamic therapy. Current Medicinal Chemistry, 13(18), 2189–2204.

Kim, N.-S., & Kang, S. (2014). Meeting report: genome engineering, plant genetics and young scientist forum. Genes & Genomics, 36(6), 685–689.

Lecerf, C., Le Bourhis, X., & Adriaenssens, E. (2019). The long non-coding RNA H19: an active player with multiple facets to sustain the hallmarks of cancer. Cellular and Molecular Life Sciences, 76(23), 4673–4687.

Li-Weber, M., Palfi, K., Giaisi, M., & Krammer, P. H. (2005). Dual role of the anti-inflammatory sesquiterpene lactone: regulation of life and death by parthenolide. Cell Death and Differentiation, 12(4), 408–409.

Li, X., Pan, X., Zhang, H., Lei, D., Liu, D., Xu, F., & Luan, X. (2008). Overexpression of cFLIP in head and neck squamous cell carcinoma and its clinicopathologic correlations. Journal of Cancer Research and Clinical Oncology, 134(5), 609–615.

Molloy, N. H., Read, D. E., & Gorman, A. M. (2011). Nerve growth factor in cancer cell death and survival. Cancers, 3(1), 510–530. Retrieved from http://www.mdpi.com/2072-6694/3/1/510/pdf

Nathan, A. J., & Scobell, A. (2012). How China sees America. Foreign Affairs, 91(5), 1689–1699.

Probst, L., Daechert, J., Schenk, B., & Fulda, S. (2017). Lipoxygenase inhibitors protect acute lymphoblastic leukemia cells from ferroptotic cell death. Biochemical Pharmacology, 140, 41–52.

Rushworth, S. A., & Micheau, O. (2009). Molecular crosstalk between trail and natural antioxidants in the treatment of cancer. British Journal of Pharmacology, 157(7), 1186–1188.

Sahin, F., Pamukcu, C., & Yalvac, E. (2013). Effect of mesenchymal cell delivered TRAIL and/or Dkk-1 on cancer cell viability. Febs Journal, 280, 453.

Seo, S. U., Kim, T. H., Kim, D. E., Min, K.-J., & Kwon, T. K. (2017). NOX4-mediated ROS production induces apoptotic cell death via down-regulation of c-FLIP and Mcl-1 expression in combined treatment with thioridazine and curcumin. Redox Biology, 13, 608–622.

Shahshahan, M. A., Beckley, M. N., & Jazirehi, A. R. (2011). Potential usage of proteasome inhibitor bortezomib (Velcade, PS-341) in the treatment of metastatic melanoma: Basic and clinical aspects. American Journal of Cancer Research, 1(7), 913–924. Retrieved from http://www.ajcr.us/files/ajcr0000082.pdf

Tosi, P., Pallotti, M. C., Pantani, L., & Petrucci, A. (2008). Novel proteasome inhibitors. Haematologica Meeting Reports, 2(5), 143–145.

Uckun, F. M. (2008). Clinical potential of targeting Bruton's tyrosine kinase. International Reviews of Immunology, 27(1), 43–69.

Wei, Z., Jiang, X., & Zhou, X. (2007). [Clinical significance and expression of FLIP and PTEN protein in laryngeal squamous cell carcinoma]. Lin Chuang Er Bi Yan Hou Tou Jing Wai Ke Za Zhi = Journal of Clinical Otorhinolaryngology, Head, and Neck Surgery, 21(3), 103–106.

Wu, C.-S., Chang, K.-P., Chen, L.-C., Chen, C.-C., Liang, Y., Hseuh, C., & Chang, Y.-S. (2012). Heterogeneous ribonucleoprotein K and thymidine phosphorylase are independent prognostic and therapeutic markers for oral squamous cell carcinoma. Oral Oncology, 48(6), 516–522.

Xiang, Z., Qu, F., Qi, L., Zhang, Y., Tong, Y., & Yu, Z. (2013). Cloning, characterization and expression analysis of a caspase-8 like gene from the Hong Kong oyster, Crassostrea hongkongensis. Fish & Shellfish Immunology, 35(6), 1797–1803.

Yao, W., Yue, P., Khuri, F. R., & Sun, S.-Y. (2015). The BET bromodomain inhibitor, JQ1, facilitates c-FLIP degradation and enhances TRAIL-induced apoptosis independent of BRD4 and c-Myc inhibition. Oncotarget, 6(33), 34669–34679.

Zhan, S., Wang, T., Ge, W., & Li, J. (2018). Multiple roles of Ring 1 and YY1 binding protein in physiology and disease. Journal of Cellular and Molecular Medicine, 22(4), 2046–2054.

Zhang, L., Zhang, W., Wang, Y.-F., Liu, B., Zhang, W.-F., Zhao, Y.-F., … Sun, Z.-J. (2015). Dual induction of apoptotic and autophagic cell death by targeting survivin in head neck squamous cell carcinoma. Cell Death and Disease, 6(5).

Zheng, Z., Cheng, S., Wu, W., Wang, L., Zhao, Y., Shen, Y., … Zhao, W.-L. (2014). c-FLIP is involved in tumor progression of peripheral T-cell lymphoma and targeted by histone deacetylase inhibitors. Journal of Hematology & Oncology, 7, 88.

- Meeting abstract (n=22)

Bowman, B. M., Galban, S., Hoff, B. A., Heist, K. A., Boes, J. L., Galban, C. J., … Rehemtulla, A. (2014). A requirement for FADD and its phosphorylation for KRAS-driven oncogenesis. Cancer Research, 74(19).

Burrows, F. J., Kessler, L., Wu, T., Gao, X., Chen, J., Hansen, R., … Liu, Y. (2018). 11q13 amplification selects for sensitivity to the ERK inhibitor KO-947 in squamous cell carcinomas. Cancer Research, 78(13).

Chang, K.-P., Chen, T.-W., Pickering, C. R., Myers, J. N., & Chang, Y.-S. (2017). Integrated omics analyses identify prognostic biomarkers of oral cavity squamous cell carcinoma in Taiwan. Clinical Cancer Research, 23(23).

David, E., Sinha, R., Kaufman, J. L., & Lonial, S. (2006). Perifosine induces DR4/DR5 expression leading to apoptosis that can be enhanced with exogenous TRAIL, and blocked with strategies directed at DR4/DR5 inhibition. Blood, 108(11), 984A-984A.

Ding, H., Han, C., Zhu, J., Chen, C.-S., & D'Ambrosio, S. (2004). Celecoxib derivatives induce apoptosis via the disruption of mitochondrial membrane potential. Proceedings of the American Association for Cancer Research Annual Meeting, 45, 168.

Gibcus, J. H., Mastik, M., Menkema, L., Bock, T. De, He, S., Takes, R. P., … Schuuring, E. (2006). FADD is amplified and overexpressed in laryngeal carcinomas, and overexpression is correlated with the presence of lymph node metastasis. Proceedings of the American Association for Cancer Research Annual Meeting, 47, 605–606.

Gibcus, J. H., Menkema, L., Mastik, M., Hermsen, M., Meijer, G., Kok, K., … Schuuring, E. (2005). Chromosomal structure of the amplified region 11q13. Proceedings of the American Association for Cancer Research Annual Meeting, 46, 209.

Jun, K. H., Pfahl, M., & Lotan, R. (2004). Induction of apoptosis by the synthetic retinoid MX350-1 through mitochondrial and death receptor mediated pathways in Head and Neck squamous carcinoma cells. Proceedings of the American Association for Cancer Research Annual Meeting, 45, 776.

Keck, M. K., Zuo, Z., Khattri, A., Braegelmann, J., Lingen, M., Rieke, D., … Seiwert, T. Y. (2012). Detection of copy number alterations in 124 head and neck squamous cell carcinomas using the Nanostring nCounter assay. Cancer Research, 72(8).

Kim, P. K., Huang, X., Billiar, T. R., & Gollin, S. M. (2003). Squamous cell carcinomas with chromosome 11q13 amplification express increased FADD, survivin, and XIAP proteins. Proceedings of the American Association for Cancer Research Annual Meeting, 44, 625.

Lepikhova, T., Pehkonen, H., Louhimo, R., Chen, P., Peitsaro, N., Nieminen, A., … Monni, O. M. (2011). An integrated approach for functional analysis of 11q13 amplification target genes in HNSCC. Cancer Research, 71(8).

Liao, C.-J., Chien, H.-T., Huang, S.-F., Chen, I.-H., Liao, C.-T., Wang, H.-M., & Hsieh, L.-L. (2015). Clinical implications of FADD copy number gain/amplification and high protein expression in areca-quid-associated oral cavity squamous cell carcinomas. Cancer Research, 75(15).

Micheau, O., Solary, E., Hmmann, A., & Dimanche-Boitrel, M. T. (1998). Increased Fas-dependent mediated apoptosis by cytotoxic drugs involves FADD and Caspase 3. Proceedings of the American Association for Cancer Research Annual Meeting, 39, 581–582.

Park, H., Zhang, Y., Xu, C., Chen, C., Wang, P., MÃ©ndez, E., & Mendez, E. (2019). Abstract LB-169: Comprehensive analysis of the expression of eight genes as a prognostic marker in head and neck cancer. Cancer Research, 79(13).

Rasamny, J. K. J., Allak, A., Frierson, H., Policarpio-Nicolas, M., Jo, V., & Jameson, M. J. (2011). Cyclin D1 and fadd as biomarkers in head and neck squamous cell carcinoma. Otolaryngology - Head and Neck Surgery, 145, 62–63.

Reddy, R. B., Hedne, N., Kuriakose, M. A., & Suresh, A. (2017). Role of ano1 in resistance/response to head and neck squamous cell carcinoma (HNSCC). Head and Neck, 39, E105–E105.

Richardson, P., Schlossman, R. L., Mitsiades, C. S., Munshi, N. C., Colson, K., Doss, D., … Anderson, K. C. (2004). Phase I clinical trial of oral administration of the histone deacetylase (HDAC) inhibitor suberoylanilide hydroxamic acid (SAHA) in patients with relapsed/refractory multiple myeloma. Blood, 104(11), 420A-420A.

Rushatamukayanunt, P., Morita, K.-I., Matsukawa, S., Tomioka, H., Harada, H., & Omura, K. (2013). Association between human papillom avirus and oral squamous cell carcinoma in young Japanese patients. Oral Oncology, 49, S130–S130.

Singh, N., Sahu, D. K., Tripathi, R. K., Mishra, A., Singh, S. K., Chowdhry, R., … Bhatt, M. L. (2018). Characterization of oral squamous cell carcinoma transcriptome through long read sequencing technology. Cancer Research, 78(13).

Sugahara, K., Michikawa, Y., Ishikawa, K., Otsuka, Y., Iwakawa, M., Shibahara, T., & Imai, T. (2007). Whole genome-wide screening of cervical lymph node metastasis-associated genetic alterations in oral squamous cell carcinoma of Japanese patients. Ejc Supplements, 5(4), 95.

Thariat, J., Grall, D., Etienne-Grimaldi, M., Penault-Llorca, F., Francoual, M., Milano, G. A., … Schilling, E. V.-O. (2010). Correlative analyses between EGFR pathways and stroma-tumor profiles in head and neck cancer patients (HNSCC) on postoperative radiotherapy plus cisplatin (PORTc) plus or minus gefitinib (GORTEC-GETTEC CARISSA trial): Implications for sensitivity to gefitinib. Journal of Clinical Oncology, 28(15).

Walter, V., Du, Y., Yin, X., Sun, W., Wilkerson, M. D., Hayward, M. C., … Hayes, D. N. (2015). Integrated genomic analysis of chromosome 11 in squamous tumors. Cancer Research, 75(15).

- Review (n=15)

Fuentes-Gonzalez, A. M., Contreras-Paredes, A., Manzo-Merino, J., Lizano, M., Fuentes-González, A. M., Contreras-Paredes, A., … Lizano, M. (2013). The modulation of apoptosis by oncogenic viruses. Virology Journal, 10.

Galluzzi, L., Kepp, O., Trojel-Hansen, C., & Kroemer, G. (2012). Non-apoptotic functions of apoptosis-regulatory proteins. EMBO Reports, 13(4), 322–330.

Jendrossek, V. (2013). Targeting apoptosis pathways by Celecoxib in cancer. Cancer Letters, 332(2), 313–324.

Leemans, C. R., Braakhuis, B. J. M., & Brakenhoff, R. H. (2011). The molecular biology of head and neck cancer. Nature Reviews Cancer, 11(1), 9–22.

Leonard, B. C., & Johnson, D. E. (2018). Signaling by cell surface death receptors: Alterations in head and neck cancer. Advances in Biological Regulation, 67, 170–178.

Orzedala-Koszel, U., Rahnama, M., Lobacz, M., & Koszel, H. (2014). Apoptotic factors in physiological and pathological processes of teeth and periodontal tissues - Literature review. Current Issues in Pharmacy and Medical Sciences, 27(4), 229–233.

Pandey, M., Shrivastava, M., Mishra, S., & Bathri, R. (2017). Role of X-linked inhibitor of apoptosis protein as cancer biomarker. Journal of Clinical and Diagnostic Research, 11(12), XE01–XE05.

Ramos-García, P., Ruiz-Ávila, I., Gil-Montoya, J. A., Ayén, Á., González-Ruiz, L., Navarro-Triviño, F. J., … GonzÃ¡lez-Moles, M. Ã. (2017). Relevance of chromosomal band 11q13 in oral carcinogenesis: An update of current knowledge. Oral Oncology, 72, 7–16.

Santarius, T., Shipley, J., Brewer, D., Stratton, M. R., & Cooper, C. S. (2010). A census of amplified and overexpressed human cancer genes. Nature Reviews Cancer, 10(1), 59–64.

Sepiashvili, L., Bruce, J. P., Huang, S. H., O'Sullivan, B., Liu, F.-F., & Kislinger, T. (2015). Novel insights into head and neck cancer using next-generation "Omic" technologies. Cancer Research, 75(3), 480–486.

Sharma, R., Kumar, R., Kodwani, R., Kapoor, S., Khare, A., Bansal, R., … Kumar, S. (2016). A review on mechanisms of anti tumor activity of chalcones. Anti-Cancer Agents in Medicinal Chemistry, 16(2), 200–211.

Sun, S.-Y. (2011). Understanding the Role of the Death Receptor 5/FADD/caspase-8 Death Signaling in Cancer Metastasis. Molecular and Cellular Pharmacology, 3(1), 31–34.

Tavassoli, M., Guelen, L., Luxon, B. A., & GÃ¤ken, J. (2005). Apoptin: Specific killer of tumor cells? Apoptosis, 10(4), 717–724.

Wilkerson, P. M., & Reis-Filho, J. S. (2013). the 11q13-q14 amplicon: Clinicopathological correlations and potential drivers. Genes Chromosomes and Cancer, 52(4), 333–355.

Wong, R. S. Y. (2011). Apoptosis in cancer: From pathogenesis to treatment. Journal of Experimental and Clinical Cancer Research, 30(1).

- Potentially malignant disorders (n=3)

Bergshoeff, V. E., der Heijden, S. J. A., Haesevoets, A., Litjens, S. G. H., Bot, F. J., Voogd, A. C., … Speel, E.-J. M. (2014). Chromosome instability predicts progression of premalignant lesions of the larynx. Pathology, 46(3), 216–224.

Wu, M.-H., Luo, J.-D., Wang, W.-C., Chang, T.-H., Hwang, W.-L., Lee, K.-H., … Chiang, W.-F. (2018). Risk analysis of malignant potential of oral verrucous hyperplasia: A follow-up study of 269 patients and copy number variation analysis. Head and Neck, 40(5), 1046–1056.

Yoshimoto, M. (2012). [Detection of FADD gene amplification in oral leukoplakia]. Kokubyo Gakkai Zasshi. The Journal of the Stomatological Society, Japan, 79(2), 71–81.

- Bioinformatics analysis of microarray datasets (n=3)

Reddy, R. B., Bhat, A. R., James, B. L., Govindan, S. V., Mathew, R., Ravindra, D. R., … Suresh, A. (2016). Meta-Analyses of Microarray Datasets Identifies ANO1 and FADD as Prognostic Markers of Head and Neck Cancer. PloS One, 11(1), e0147409–e0147409.

Pérez-Sayans, M. P., Petronacci, C. M. C., Pouso, A. I. L., Iruegas, E. P., Carrion, A. B., Penaranda, J. M. S., … Garcia Garcia, A. (2019). Comprehensive genomic review of TCGA head and neck squamous cell carcinomas (HNSCC). Journal of Clinical Medicine, 8(11), 1896.

Yang, B., & Bao, X. (2015). Identification of genes associated with laryngeal squamous cell carcinoma samples based on bioinformatic analysis. Molecular Medicine Reports, 12(3), 3386–3392.

- Polymorphism (n=1)

Eun, Y. G., Chung, D. H., Kim, S. W., Lee, Y. C., Kim, S. K., & Kwon, K. H. (2014). A Fas-associated via death domain promoter polymorphism (rs10898853, -16C/T) as a risk factor for papillary thyroid cancer. European Surgical Research. Europaische Chirurgische Forschung. Recherches Chirurgicales Europeennes, 52(1), 1–7.

- Case report (n=1)

Gualco, G., van den Berg, A., Koopmans, S., Bacchi, L. M., Carneiro, S. S., Jr., E. R., … Chan, J. K. C. (2008). Autoimmune lymphoproliferative syndrome in a patient with a new minimal deletion in the death domain of the FAS gene. Human Pathology, 39(1), 137–141.

- Book chapter (n=1)

Bisen, P., Khan, Z., & Bundela, S. (2013). Diagnostic and Therapeutic Potential of Apoptotic Marker. Biology of Oral Cancer, 163–172.

- Comment (n=1)

Dent, P. (2013). FADD the bad in head and neck cancer. Cancer Biology & Therapy, 14(9), 780–781.

15.2 List S4. Full-text articles excluded

- Lack of essential data (n=14)

Al-Hebshi, N. N., Li, S., Nasher, A. T., El-Setouhy, M., Alsanosi, R., Blancato, J., & Loffredo, C. (2016). Exome sequencing of oral squamous cell carcinoma in users of Arabian snuff reveals novel candidates for driver genes. International Journal of Cancer, 139(2), 363–372.

Chen, T.-W., Lee, C.-C., Liu, H., Wu, C.-S., Pickering, C. R., Huang, P.-J., … Chang, Y.-S. (2017). APOBEC3A is an oral cancer prognostic biomarker in Taiwanese carriers of an APOBEC deletion polymorphism. Nature Communications, 8(1).

Giri, U., Ashorn, C. L., Ramdas, L., Stivers, D. N., Coombes, K., El-Naggar, A. K., … Story, M. D. (2006). Molecular signatures associated with clinical outcome in patients with high-risk head-and-neck squamous cell carcinoma treated by surgery and radiation. International Journal of Radiation Oncology Biology Physics, 64(3), 670–677.

Hatakeyama, M., Reis-Rosa, L. A., Marchi, F. A., Spilborghs, G., Carvalho, A. L., Ribaldo, U. M., … Rogatto, S. R. (2013). Integrative analysis of DNA copy number and gene expression in oropharyngeal squamous cell carcinomas. Cancer Research, 73(8).

Jarmuz-Szymczak, M., Pelinska, K., Kostrzewska-Poczekaj, M., Bembnista, E., Giefing, M., Brauze, D., … Szyfter, K. (2013). Heterogeneity of 11q13 region rearrangements in laryngeal squamous cell carcinoma analyzed by microarray platforms and fluorescence in situ hybridization. Molecular Biology Reports, 40(7), 4161–4171.

Järvinen, A.-K., Autio, R., Haapa-Paananen, S., Wolf, M., Saarela, M., Grénman, R., … Monni, O. (2006). Identification of target genes in laryngeal squamous cell carcinoma by high-resolution copy number and gene expression microarray analyses. Oncogene, 25(52), 6997–7008.

Lawrence, M. S., Sougnez, C., Lichtenstein, L., Cibulskis, K., Lander, E., Gabriel, S. B., … Pham, M. (2015). Comprehensive genomic characterization of head and neck squamous cell carcinomas. Nature, 517(7536), 576–582.

Lo Muzio, L., Sartini, D., Santarelli, A., Rocchetti, R., Morganti, S., Pozzi, V., … Emanuelli, M. (2014). Expression and prognostic significance of apoptotic genes in oral squamous cell carcinoma. Molecular Carcinogenesis, 53(4), 264–271.

Saberi, E., Kordi-Tamandani, D.-M., Jamali, S., & Rigi-Ladiz, M.-A. (2014). Analysis of methylation and mRNA expression status of FADD and FAS genes in patients with oral squamous cell carcinoma. Medicina Oral, Patologia Oral y Cirugia Bucal, 19(6), e562-8.

Sugahara, K., Michikawa, Y., Ishikawa, K., Shoji, Y., Iwakawa, M., Shibahara, T., & Imai, T. (2011). Combination effects of distinct cores in 11q13 amplification region on cervical lymph node metastasis of oral squamous cell carcinoma. International Journal of Oncology, 39(4), 761–769.

Tomioka, H., Morita, K.-I., Hasegawa, S., & Omura, K. (2006). Gene expression analysis by cDNA microarray in oral squamous cell carcinoma. Journal of Oral Pathology & Medicine : Official Publication of the International Association of Oral Pathologists and the American Academy of Oral Pathology, 35(4), 206–211.

Upadhyay, P., Gardi, N., Desai, S., Chandrani, P., Joshi, A., Dharavath, B., … Dutt, A. (2017). Genomic characterization of tobacco/nut chewing HPV-negative early stage tongue tumors identify MMP10 asa candidate to predict metastases. Oral Oncology, 73, 56–64.

Urashima, M., Hama, T., Suda, T., Suzuki, Y., Ikegami, M., Sakanashi, C., … Kojima, H. (2013). Distinct effects of alcohol consumption and smoking on genetic alterations in head and neck carcinoma. PloS One, 8(11), e80828–e80828.

Xu, C., Liu, Y., Wang, P., Fan, W., Rue, T. C., Upton, M. P., … Mendez, E. (2010). Integrative analysis of DNA copy number and gene expression in metastatic oral squamous cell carcinoma identifies genes associated with poor survival. Molecular Cancer, 9.

- No clinicopathological or survival outcomes (n=3)

Lo Muzio, L., Santarelli, A., Emanuelli, M., Pierella, F., Sartini, D., Staibano, S., … Rosa, G. De. (2006). Genetic analysis of oral squamous cell carcinoma by cDNA microarrays focused apoptotic pathway. International Journal of Immunopathology and Pharmacology, 19(3), 675–682.

Jablonska, E., Garley, M., Jablonsski, J., (2009). The expressions of intrinsic and extrinsic apoptotic pathway proteins in neutrophils of oral cavity cancer patients: A preliminary study. Archivum Immunologiae et Therapiae Experimentalis, 57(3), 229–234.

Jãrvinen, A.-K., Autio, R., Kilpinen, S., Saarela, M., Leivo, I., GrÃ©nman, R., … Monni, O. (2008). High-resolution copy number and gene expression microarray analyses of head and neck squamous cell carcinoma cell lines of tongue and larynx. Genes Chromosomes and Cancer, 47(6), 500–509.

- Overlapping population (n=2)

Wachters, J. E., Kop, E., Slagter-Menkema, L., Mastik, M., van der Wal, J. E., van der Vegt, B., … Schuuring, E. (2020). Distinct Biomarker Profiles and Clinical Characteristics in T1-T2 Glottic and Supraglottic Carcinomas. The Laryngoscope.

Gibcus, J. H., Mastik, M. F., Menkema, L., de Bock, G. H., Kluin, P. M., Schuuring, E., & van der Wal, J. E. (2008). Cortactin expression predicts poor survival in laryngeal carcinoma. British Journal of Cancer, 98(5), 950–955

16. List of studies included in this systematic review and meta-analysis.

List S5

Chien, H.-T., Cheng, S.-D., Chuang, W.-Y., Liao, C.-T., Wang, H.-M., & Huang, S.-F. (2016). Clinical Implications of FADD Gene Amplification and Protein Overexpression in Taiwanese Oral Cavity Squamous Cell Carcinomas. PLOS ONE, 11(10), e0164870. https://doi.org/10.1371/journal.pone.0164870

Fan, S., Müller, S., Chen, Z., Lin, P., Tighiouart, M., Shin, D., … Sun, S.-Y. (2013). Prognostic impact of Fas-associated death domain, a key component in death receptor signaling, is dependent on the presence of lymph node metastasis in head and neck squamous cell carcinoma. Cancer Biology & Therapy, 14(4), 365–369. https://doi.org/10.4161/cbt.23636

Gibcus, J. H., Menkema, L., Mastik, M. F., Hermsen, M. A., de Bock, G. H., van Velthuysen, M.-L. F., … Schuuring, E. (2007). Amplicon mapping and expression profiling identify the Fas-associated death domain gene as a new driver in the 11q13.3 amplicon in laryngeal/pharyngeal cancer. Clinical Cancer Research : An Official Journal of the American Association for Cancer Research, 13(21), 6257–6266. https://doi.org/10.1158/1078-0432.CCR-07-1247

Haili, S., Huam, G., Ting, L., & Shuifang, X. (2010). Expression and significance of Fas, FasL and Fas-related death domain proteins in laryngeal carcinoma. Chinese Otolaryngology Head and Neck Surgery, 17(7), 343–345.

Li, J., Wen, Q., Xu, L., Wang, W., Luo, J., Chu, S., … Fan, S. (2014). Fatty acid synthase–associated protein with death domain: a prognostic factor for survival in patients with nasopharyngeal carcinoma. Human Pathology, 45(12), 2447–2452. https://doi.org/10.1016/j.humpath.2014.08.010

Noorlag, R., Boeve, K., Witjes, M. J. H., Koole, R., Peeters, T. L. M., Schuuring, E., … van Es, R. J. J. (2017). Amplification and protein overexpression of cyclin D1: Predictor of occult nodal metastasis in early oral cancer. Head & Neck, 39(2), 326–333. https://doi.org/10.1002/hed.24584

Pattje, W. J., Melchers, L. J., Slagter-Menkema, L., Mastik, M. F., Schrijvers, M. L., Gibcus, J. H., … Langendijk, J. A. (2013). FADD expression is associated with regional and distant metastasis in squamous cell carcinoma of the head and neck. Histopathology, 63(2), 263–270. https://doi.org/10.1111/his.12174

Prapinjumrune, C., Morita, K., Kuribayashi, Y., Hanabata, Y., Shi, Q., Nakajima, Y., … Omura, K. (2009). DNA amplification and expression of FADD in oral squamous cell carcinoma. Journal of Oral Pathology & Medicine. https://doi.org/10.1111/j.1600-0714.2009.00847.x

Rasamny, J. J., Allak, A., Krook, K. A., Jo, V. Y., Policarpio-Nicolas, M. L., Sumner, H. M., … Jameson, M. J. (2012). Cyclin D1 and FADD as Biomarkers in Head and Neck Squamous Cell Carcinoma. Otolaryngology–Head and Neck Surgery, 146(6), 923–931. https://doi.org/10.1177/0194599811435052

Ribeiro, I. P., Marques, F., Caramelo, F., Pereira, J., Patrício, M., Prazeres, H., … Carreira, I. M. (2014). Genetic gains and losses in oral squamous cell carcinoma: impact on clinical management. Cellular Oncology (Dordrecht), 37(1), 29–39. https://doi.org/10.1007/s13402-013-0161-5

Schrijvers, M. L., Pattje, W. J., Slagter-Menkema, L., Mastik, M. F., Gibcus, J. H., Langendijk, J. A., … Schuuring, E. (2012). FADD Expression as a Prognosticator in Early-Stage Glottic Squamous Cell Carcinoma of the Larynx Treated Primarily With Radiotherapy. International Journal of Radiation Oncology*Biology*Physics, 83(4), 1220–1226. https://doi.org/10.1016/j.ijrobp.2011.09.060

van Kempen, P. M. W., Noorlag, R., Braunius, W. W., Moelans, C. B., Rifi, W., Savola, S., … Willems, S. M. (2015). Clinical relevance of copy number profiling in oral and oropharyngeal squamous cell carcinoma. Cancer Medicine, 4(10), 1525–1535. https://doi.org/10.1002/cam4.499

Wachters, J. E., Schrijvers, M. L., Slagter-Menkema, L., Mastik, M., Langendijk, J. A., de Bock, G. H., … Schuuring, E. (2017). Phosphorylated FADD is not prognostic for local control in T1-T2 supraglottic laryngeal carcinoma treated with radiotherapy. The Laryngoscope, 127(9), E301–E307. https://doi.org/10.1002/lary.26563

17. Protocol

Review title

Prognostic and clinicopathological significance of FADD alterations in head and neck squamous cell carcinoma: a systematic review and meta-analysis protocol.

Anticipated or actual start date.

Oct, 2019

Anticipated completion date.

Sept, 2020

Stage of review at time of protocol submission (Feb 2020)

Review stage	Started	Completed
Preliminary searches	Yes	Yes
Piloting of the study selection process	Yes	Yes
Formal screening of search results against eligibility criteria	Yes	Yes
Data extraction	No	No
Risk of bias (quality) assessment	No	No
Data analysis	No	No

Review team members and their organisational affiliations.

Pablo Ramos-Garcia - School of Dentistry, University of Granada, Granada, Spain

Miguel Ángel González-Moles - School of Dentistry, University of Granada, Granada, Spain

Ángela Ayén - Dermatology Service, San Cecilio Hospital Complex, Granada, Spain.

Teresa de Porras Carrique - School of Dentistry, University of Granada., Granada, Spain

Isabel González-Ruiz - School of Dentistry, University of Granada., Granada, Spain

Lucia González-Ruiz - Dermatology Service, Ciudad Real General University Hospital, Ciudad Real, Spain.

Isabel Ruiz-Ávila - Pathology Service, San Cecilio Hospital Complex, Granada, Spain

Funding sources/sponsors.

None

Conflicts of interest.

None

Review question.

What is the clinicopathological and prognostic significance of FADD alterations in patients with head and neck squamous cell carcinoma?

Searches.

Studies published in PubMed, Embase, Web of Science and Scopus (upper limit=February 2020), using both database thesaurus terms (*i.e.* MeSH or Emtree terms) and free text words:

PubMed - ("Fas-Associated Death Domain Protein"[MH] OR (“fas”[ALL] N4 “associated”[ALL] N4 “death”[ALL] N4 “domain”[ALL]) OR "FADD"[ALL]) AND (“head”[MH] OR “head”[ALL] OR “neck”[MH] OR “neck”[ALL] OR "mouth"[MH] OR "mouth"[ALL] OR "oral"[ALL] OR "pharynx”[MH] OR pharyn*[ALL] OR oropharyn*[ALL] OR nasopharyn*[ALL] OR hypopharyn*[ALL] OR "larynx”[MH] OR laryn*[ALL] OR "nose”[MH] OR “nose”[ALL] OR “nasal”[ALL]) AND ("carcinoma, squamous cell"[MH] OR "carcinoma"[ALL] OR "Head and Neck Neoplasms"[MH] OR neoplas*[ALL] OR “cancer”[ALL])

Embase - ('Fas associated death domain protein'/exp OR ('fass' N4 'associated' N4 'death' N4 'domain') OR 'FADD') AND ('head'/exp OR 'head' OR 'neck'/exp OR 'neck' OR 'mouth'/exp OR 'mouth' OR 'oral' OR 'pharynx'/exp OR 'pharyn*' OR 'oropharyn*' OR 'nasopharyn*' OR 'hypopharyn*' OR 'larynx'/exp OR 'laryn*' OR 'nose'/exp OR 'nose' OR 'nasal') AND ('squamous cell carcinoma'/exp OR 'carcinoma' OR 'head and neck cancer'/exp OR 'cancer' OR 'neoplas*')

Web of Science - TS=(FADD OR “Fas associated death domain protein”) AND TS=(head OR neck OR mouth OR oral OR pharyn* OR oropharyn* OR nasopharyn*OR hypopharyn* OR laryn* OR nose OR nasal) AND TS=(“squamous cell carcinoma” OR neoplas* or cancer)

Scopus - TITLE-ABS-KEY((“FADD” OR “Fas associated death domain protein”) AND ("head" OR "neck" OR "mouth" OR "oral" OR "pharyn*" OR "oropharyn*" OR "nasopharyn*" OR "hypopharyn*" OR "laryn*" OR "nose" OR "nasal") AND (“squamous cell carcinoma” OR “neoplas*” or “cancer”))

An additional screening will also be performed handsearching the reference lists of retrieved included studies and using Google Scholar.

Condition or domain being studied.

Head and neck cancer is the sixth most common type of cancer, accounting for approximately 600,000 new cases and 300,000 cancer deaths worldwide annually. Around 90% of these neoplasms are histologically squamous cell carcinomas. The overall 5-year survival rate of head and neck squamous cell carcinoma (HNSCC) - which is approximately of 50%- has not changed significantly in the past decades. In clinical practice, prognostic evaluation of patients with HNSCC is mainly based on traditional TNM classification. Therefore, the future identification and validation of prognostic molecular markers is needed to identify high risk patients, and predict treatment response.

Participants/population.

Patients diagnosed with head and neck squamous cell carcinoma. Studies researching patients with distinct anatomical location or histopathological type will be excluded.

Intervention(s), exposure(s).

We will evaluate studies in which the FADD alterations (*FADD* amplification or pFADD/FADD overexpression) were evaluated in tumor biopsies from patients with HNSCC.

Differences in the amplification levels of *FADD* will be categorized as positive for the exposition group, and negative for the control group, based on the cut-off value chosen by the authors. Differences in the expression of FADD and/or pFADD will be categorized as high expression or overexpression for the exposition group, and low expression for the control group, based on the cut-off value chosen by the authors.

Comparator(s)/control.

Control group will be represented by the group of patients with HNSCC and negative *FADD* amplification or low pFADD or FADD expression.

Primary outcome(s).

Prognostic variables: overall survival, diseases-specific survival, diseases-free survival, local recurrence.

Clinicopathological variables: T and N status, clinical stage and histological grade.

Secondary outcome(s).

- Although survival variables are logically the most relevant parameters, all will be considered as primary outcomes.

Types of study to be included.

Inclusion criteria will be:

- Original research articles published in all languages, without time or study design restrictions.

- FADD alterations evaluated in human HNSCCs.

- Analysis of the outcomes of interest (please, see below) and their relationships with FADD alterations.

Exclusion criteria will be:

-Retractions, case reports, editorials, letters, personal opinions or comments, meeting abstracts, books, reviews or meta-analyses.

- in vitro or animal research.

- No HNSCC.

- Other FADD alterations (e.g., polymorphisms) and the combined assessment of the amplification of the set of genes of 11q13 chromosomal band (without specific data for *FADD*).

- Lack essential data for OR/HR (with 95%CI) estimations.

Data extraction

Data will be gathered on the first author, publication year, country, publication language, sample size, FADD alteration under study, methodology, and the frequency of alterations, tumor location, sex and age of patients, tobacco and alcohol consumption, recruitment period, funding and potential conflict of interest, treatment modality, follow-up period and study design. In immunohistochemical studies, information will be also recorded on the anti-FADD antibody, intracellular immunostaining (nuclear/cytoplasmic/mixed), cutoff point and scoring system.

Risk of bias (quality) assessment.

The risk of bias in individual studies will be assessed using the Quality in Prognostic Studies (QUIPS) tool, developed by Cochrane prognosis methods group. Specifically, it contains 6 domains: study participation, study attrition, prognostic factor measurement, outcome measurement, study confounding and statistical analysis and reporting. Each domain will be rated as low, moderate or high risk of bias for each study. An overall rating will be also assigned to individual studies for statistical purposes (i.e., to explore the potential influence of quality/risk of bias on pooled estimates).

Strategy for data synthesis.

Odds ratios (OR) and 95% confidence intervals (CI) will be used as the measure of association to determine the correlations between FADD alterations and clinicopathological features (T status [T3/4 vs, T1/2], N status [N+ vs, N-], clinical stage [III/IV vs, I/II] and histological grade [II/III vs, I]).

Hazard ratios (HR) and 95% CI will be used as the measure of association to estimate the impact of FADD alterations on time-to-event parameters (OS, DSS, DFS and LR). If HR with 95%CI are not explicitly reported by the authors, they will be calculated by us using Parmar and Tierney methods. If only Kaplan-Meier curves are reported, HR data will be extracted using En Engauge Digitizer 4.1 software. In both meta-analyses, if data are not reported as OR or HR, different ratio metrics will be extracted and pooled as an approximation of these measures if appropriate (rare outcomes under study (<5%) and an effect size not too high or low). If these measures derived both from univariable and multivariable models, data will be extracted from multivariable, reflecting a greater adjustment for potentially confounding variables.

In meta-analysis, OR and HR will be pooled where appropriate (taking into account heterogeneity degree with a low number of studies, making it impossible to assess their potential sources) using random effects models, which accounts for the possibility that are different underlying results among study subpopulations (i.e., HNSCC subsites, geographical differences, or based on different experimental methods). Forest plots will be constructed to examine the overall effect. Heterogeneity between studies will be checked using the χ^2 based Cochran's Q test ($p<0.10$) and Higgins I^2 statistic. Subgroups analyses, meta-regression and sensitivity analyses will also be performed (please, see next above).

Finally, funnel plots will be constructed where appropriate, to assess small-study effects such as publication bias. Egger's and Peters's tests ($p<0.10$) will also be used to statistically assess funnel plots asymmetry. Stata v.14.1 will be employed for all tests, using commands written by the user.

Analysis of subgroups or subsets.

Preplanned subgroup (geographical area, HNSCC subsite, anti-FADD antibody, and immunohistochemical pattern) and meta-regression (sex, age, clinical stage and follow up period) analyses will be performed to explore the relations between the precedent outcomes in these subgroups. If a low number of studies are included in meta-regression analyses, bootstrap methods will be implemented to improve the precision of estimations.

Finally, sensitivity analyses will be performed to explore the influence of individual and subsets of studies (by quality and source of data, i.e., derived from curves, univariable or multivariable models) on the estimation of the overall effect, to test the reliability of the overall pooled results.
